# Transcriptional activation of PINK1 by MyoD1 mediates mitochondrial homeostasis to induce renal calcification in pediatric nephrolithiasis

**DOI:** 10.1038/s41420-024-02117-w

**Published:** 2024-09-06

**Authors:** Kaiping Zhang, Xiang Fang, Ye Zhang, Yin Zhang, Min Chao

**Affiliations:** Department of Urology, Anhui Provincial Children’s Hospital/Children’s Hospital of Fudan University (Affiliated Anhui Branch), Hefei, 230000 PR China

**Keywords:** Paediatric kidney disease, Kidney

## Abstract

This study aims to uncover the molecular mechanisms underlying pediatric kidney stone formation induced by renal calcium deposition by utilizing high-throughput sequencing data to reveal the regulation of PINK1 by MyoD1. We performed transcriptome sequencing on peripheral blood samples from healthy children and children with kidney stones to obtain differentially expressed genes (DEGs). Genes related to mitochondrial oxidative stress were obtained from the Genecards website and intersected with DEGs to obtain candidate target genes. Additionally, we conducted protein-protein interaction (PPI) analysis using the STRING database to identify core genes involved in pediatric kidney stone disease (KSD) and predicted their transcription factors using the hTFtarget database. We assessed the impact of MyoD1 on the activity of the PINK1 promoter using dual-luciferase reporter assays and investigated the enrichment of MyoD1 on the PINK1 promoter through chromatin immunoprecipitation (ChIP) experiments. To validate our hypothesis, we selected HK-2 cells and established an in vitro kidney stone model induced by calcium oxalate monohydrate (COM). We evaluated the expression levels of various genes, cell viability, volume of adherent crystals in each group, as well as mitochondrial oxidative stress in cells by measuring mitochondrial membrane potential (Δψm), superoxide dismutase (SOD) activity, reactive oxygen species (ROS), and malondialdehyde (MDA) content. Mitochondrial autophagy was assessed using mtDNA fluorescence staining and Western blot analysis of PINK1-related proteins. Apoptosis-related proteins were evaluated using Western blot analysis, and cell apoptosis was measured using flow cytometry. Furthermore, we developed a rat model of KSD and assessed the expression levels of various genes, as well as the pathologic changes in rat renal tissues using H&E and von Kossa staining, transmission electron microscopy (TEM), and the expression of creatinine, blood urea nitrogen, neutrophil gelatinase-associated lipocalin (NGAL), and kidney injury molecule-1 (KIM-1) to evaluate the mitochondrial oxidative stress in vivo (through measurement of Δψm, SOD activity, ROS, and MDA content). Mitochondrial autophagy was evaluated by Western blot analysis of PINK1-associated proteins. Apoptosis-related proteins were detected using Western blot analysis, and cellular apoptosis was examined using cell flow cytometry and TUNEL staining. Bioinformatics analysis revealed that the PINK1 gene is upregulated and vital in pediatric kidney stone patients. Our in vitro and in vivo experiments demonstrated that silencing PINK1 could inhibit kidney stone formation by suppressing mitochondrial oxidative stress both in vitro and in vivo. We identified MyoD1 as an upstream transcription factor of PINK1 that contributes to the occurrence of pediatric kidney stones through the activation of PINK1. Our in vivo and in vitro experiments collectively confirmed that silencing MyoD1 could inhibit mitochondrial oxidative stress, mitochondrial autophagy, and cellular apoptosis in a rat model of kidney stones by downregulating PINK1 expression, consequently suppressing the formation of kidney stones. In this study, we discovered that MyoD1 may promote kidney stone formation and development in pediatric patients by transcriptionally activating PINK1 to induce mitochondrial oxidative stress.

## Introduction

Pediatric renal calculi is a common childhood disease affecting children’s physical and mental health [1–4]. In recent years, with the continuous development of medical technology and the deepening of research, we have gained a deeper understanding of the pathogenesis of renal calculi. However, the specific molecular and biological mechanisms are still not fully understood [5–8]. This study focuses on two critical proteins - MyoD1 and PINK1 [9, 10]. MyoD1 is a transcription factor that plays a vital role in muscle cell differentiation, while PINK1 plays a central role in regulating mitochondrial homeostasis and the response to oxidative stress [11]. Recent studies have found that these two proteins play essential roles in developing many diseases, including neurodegenerative diseases, muscular diseases, and various types of tumors [12, 13].

Firstly, we will utilize high-throughput sequencing technology to perform transcriptome sequencing on peripheral blood samples from pediatric patients with kidney stones and healthy children. High-throughput sequencing technology provides us with a comprehensive and precise way to understand the expression of genes. It could help us identify gene expression differences between diseased and non-diseased tissues, which is crucial for identifying critical genes related to diseases in the future [14–16]. Using this data, we could identify genes with expression differences in pediatric nephrolithiasis patients, which may be critical factors influencing disease development [17].

Next, we will investigate the relationship between these genes with expression differences and mitochondrial oxidative stress. Through the Genecards website, we could obtain genes related to mitochondrial oxidative stress and then intersect them with the previously obtained differentially expressed genes. This way, we could identify candidate target genes differentially expressed in kidney stone patients and related to mitochondrial oxidative stress. By conducting in-depth research on these genes, we could uncover the mechanisms behind the occurrence of new diseases and provide new insights for developing treatment strategies.

Then, we conducted protein-protein interaction network analysis and transcription factor prediction to identify the core genes involved in pediatric kidney stone disease (KSD) and the transcription factors that may regulate the expression of these genes. The interplay between proteins is crucial for understanding the biochemical processes within organisms and the mechanisms underlying disease. Transcription factors, conversely, control gene expression and are an integral part of the regulatory network governing gene expression in organisms.

In this study, we validate our hypothesis through in vivo and in vitro experiments. By constructing an in vitro renal stone cell model and an in vivo renal stone rat model, we could visually observe the roles of MyoD1 and PINK1 in the occurrence of renal stones. Through experimental verification, we will reveal how MyoD1 activates the transcription of PINK1, thereby impacting mitochondrial oxidative stress and promoting the occurrence of kidney stones. This discovery will provide important clues for our understanding of the pathogenesis of kidney stones and the development of new treatment methods.

## Results

### PINK1 gene plays a crucial role in the occurrence and development of pediatric nephrolithiasis

Pediatric kidney stones are a common type of kidney disease, and their incidence gradually increases [4, 18].

**Fig. 1:**
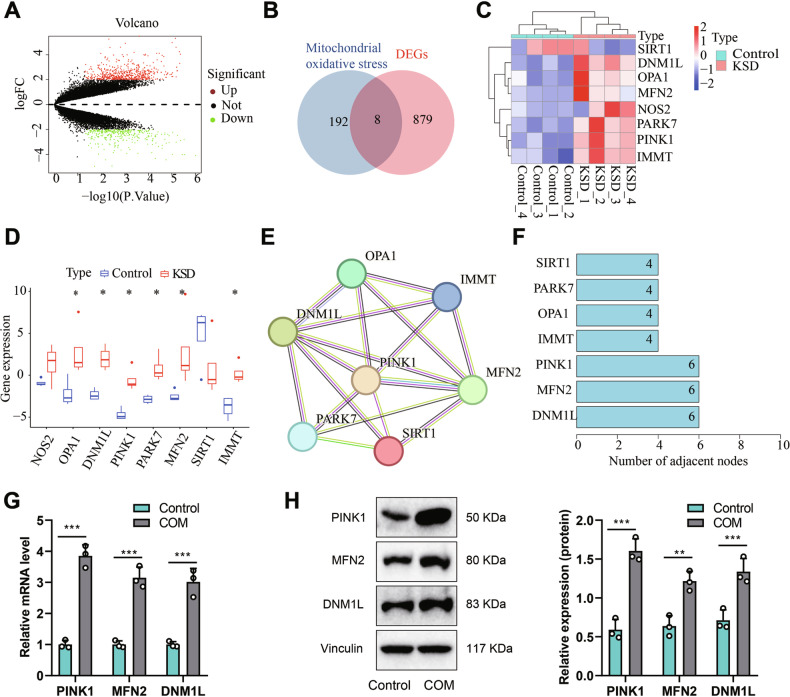
Identification of target genes involved in pediatric kidney stone formation through bioinformatics analysis. **A** Volcano plot of differentially expressed mRNA (x-axis represents -log10 *p*-value, y-axis represents logFC). Green dots represent downregulated genes, red dots represent upregulated genes, and black dots represent no difference. Control group *n* = 4, Model group *n* = 4. **B** Venn diagram showing the intersection between (Differentially Expressed Genes) DEGs and mitochondrial oxidative stress genes in the Genecards database. **C** Heatmap of differentially expressed genes (x-axis represents sample number, y-axis represents gene names). The dendrogram above represents sample clustering, and the histogram in the upper left corner represents the color gradient. **D** Expression profile of differentially expressed genes (Control group *n* = 4, (kidney stone disease) KSD group *n* = 4). **E** Protein interaction network diagram of candidate genes based on the String database. **F** Degree values of protein interactions encoded by intersecting candidate genes. **G** Expression levels of PINK1, MFN2, and DNM1L in HK-2 cells after (calcium oxalate monohydrate) COM stimulation, as detected by Real-Time quantitative Polymerase Chain Reaction (RT-qPCR). **H** Expression levels of PINK1, MFN2, and DNM1L in HK-2 cells after COM stimulation, as detected by Western blot. *P* < 0.05, **P < 0.001. All cell experiments were repeated 3 times.

Although extensive research has been conducted, the pathogenesis of pediatric nephrolithiasis remains highly complex and needs to be fully understood. To further investigate the potential molecular mechanisms of pediatric kidney stones, we performed transcriptome sequencing on peripheral blood samples from healthy children and children with pediatric kidney stones and identified 887 differentially expressed genes (Fig. 1A).

Later, we utilized the Genecards database to search for genes associated with mitochondrial oxidative stress and compared them with the differentially expressed gene sequences. We identified 8 mitochondrial oxidative stress genes differentially expressed in pediatric kidney stones (Fig. 1B–D). We imported these 8 candidate genes encoding proteins into the String database, obtaining a protein-protein interaction network (Fig. 1E), and displayed the number of interaction targets for each protein using Degree values (Fig. 1F).

In our subsequent analyses, we included PINK1, MFN2, and DNM1L as the target genes. Subsequently, we treated HK-2 cells with COM and established an in vitro kidney stone model. Based on the RT-qPCR and WB results (Fig. 1G–H), we observed the most significant differential expression of PINK1’s mRNA and protein in the COM group. Given PINK1’s central position and highest number of interaction targets in protein-protein interactions, we selected PINK1 for further experimental validation. Existing research has demonstrated that inhibiting PINK1 can mitigate oxidative stress and alleviate acute kidney injury [19], further justifying our focus on PINK1. In addition, existing literature has shown that inhibition of PINK1 could suppress oxidative stress and alleviate acute kidney injury [19].

Therefore, we chose PINK1 as the target gene for subsequent experimental validation. In summary, the above results indicate that in pediatric kidney stone patients, PINK1 expression is upregulated in peripheral blood, and the PINK1 gene may play an essential role in the occurrence and development of pediatric kidney stones.

### Silent PINK1 alleviates kidney stones by inhibiting mitochondrial oxidative stress in HK-2 cells

PINK1 is crucial in the cellular response to mitochondrial oxidative stress [19]. Furthermore, according to [20], PINK1 could also regulate mitochondrial membrane potential to affect mitochondrial respiratory chain function, thereby attenuating mitochondrial oxidative stress. It indicates that PINK1 plays a critical role in maintaining mitochondrial homeostasis. However, we do not know the role of PINK1 in pediatric kidney stones.

**Fig. 2:**
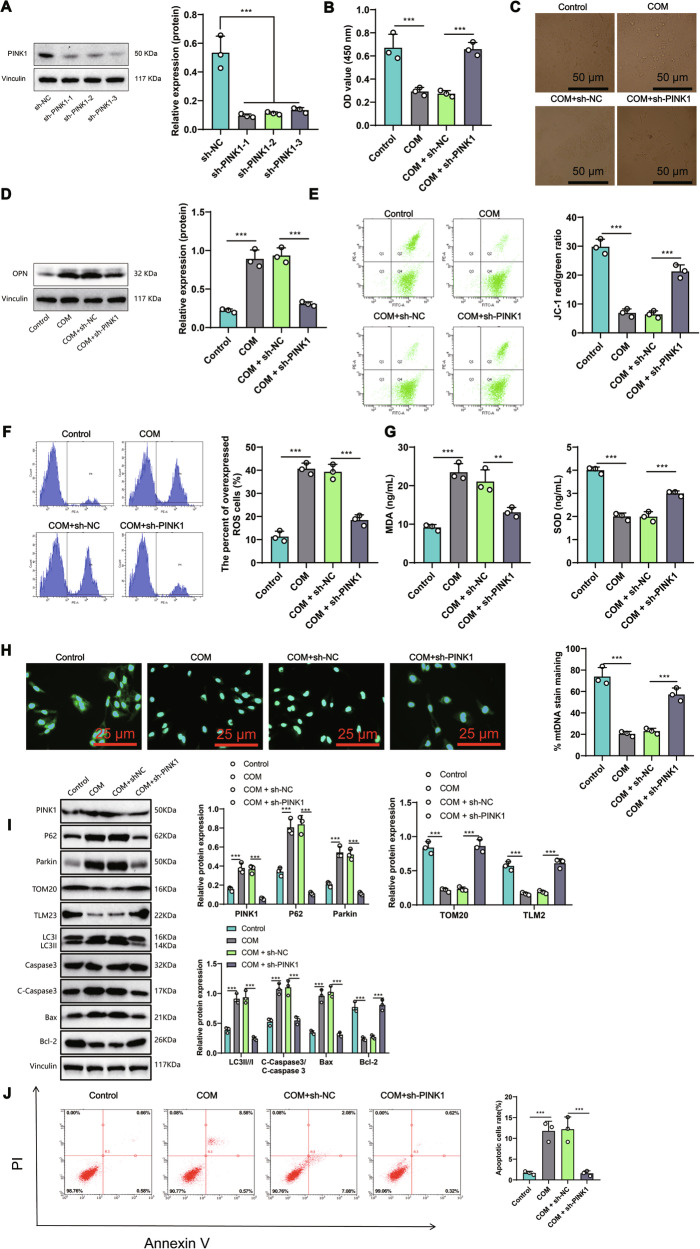
The impact of PINK1 silencing on the renal stone cell model. **A** Efficiency of PINK1 silencing as detected by Western blot. **B** The activity of HK-2 cells in various groups was measured by (Cell Counting Kit-8) CCK-8 assay. **C** Representative images (×100) showing COM crystal adhesion to each group’s surface of HK-2 cells. **D** Expression levels of the renal stone-related protein OPN in HK-2 cells of each group, as detected by Western blot. **E** Mitochondrial membrane potential (Δψm) levels in HK-2 cells of each group as measured by flow cytometry. **F** (Reactive Oxygen Species) ROS content in HK-2 cells of each group as measured by flow cytometry. **G** Levels of oxidative stress-related factors in HK-2 cells of each group. **H** Representative images of mitochondrial DNA (mtDNA) in each group were detected using immunofluorescence. **I** Changes in PINK1, p62, Parkin, TOM20, TLM2, LC3II/I, C-Caspase3/Caspase 3, Bax, and Bcl-2 in each group were detected by Western blotting. **J** The apoptosis status of cells in each group was evaluated using flow cytometry. *P < 0.05, **P < 0.01, ***P < 0.001. All cell experiments were repeated 3 times.

To elucidate the impact of PINK1 on pediatric nephrolithiasis, we utilized RNAi technology to silence PINK1 in COM-induced HK-2 cell line, a model for calcium oxalate monohydrate kidney stones. After measuring through western blot, we found that the expression level of PINK1 decreased after silencing it (Fig. 2A). Among the siRNAs used, the first one showed the best silencing effect. Therefore, we chose it for further experimental studies.

In the CCK8 experiment, a significant decrease in cell viability was observed in HK-2 cell line treated with COM, indicating successful establishment of the kidney stone model (Fig. 2B). Conversely, silencing of PINK1 led to a significant increase in cell viability post-COM treatment in HK-2 cells. Additionally, via an in vitro cell adhesion assay, it was found that silencing PINK1 reduced the attachment of COM crystals on HK-2 cells (Fig. 2C). Osteopontin (OPN) is a primary component of calcium-containing kidney stone matrix [21], with its high expression implicated in kidney stone formation [22]. Thus, the expression levels of OPN protein were assessed using Western blotting (WB) technique. The WB results (Fig. 2D) indicated a significant increase in OPN expression in the COM group compared to the control group; however, OPN expression decreased markedly after PINK1 silencing. These findings suggest that PINK1 may play a role in promoting pediatric kidney stone formation.

On the other hand, we also assessed the impact of COM treatment on cellular mitochondria by analyzing changes in mitochondrial membrane potential (Δψm) and ROS levels using flow cytometry. Compared to the control group, our results show that COM treatment leads to a decrease in cellular mitochondrial membrane potential (Δψm) and an increase in ROS levels (Fig. 2E, F). When we silence PINK1, these effects are reduced.

Further ELISA analysis revealed that silencing PINK1 could decrease the levels of MDA and increase the levels of SOD in cells after COM treatment (Fig. 2G), indicating that silencing PINK1 could alleviate cellular oxidative stress. PINK1 was found to be closely associated with mitochondrial autophagy in our study. Fluorescence experiments demonstrated that the silencing of PINK1 resulted in a higher preservation of mitochondrial DNA (mtDNA) after treatment with COM, when compared to the control group (Fig. 2H). This finding strongly indicates a decrease in the occurrence of mitochondrial autophagy. Additionally, we conducted an examination of various factors involved in mitochondrial autophagy, including the mitochondrial membrane proteins TOM20 (translocase of outer mitochondrial membrane 20 homolog) and TIM23 (translocase of inner mitochondrial membrane 23), the autophagy-related receptor p62, Parkin, and the autophagy marker LC3II/I. In the silencing PINK1 group, we observed a significant increase in the levels of both TOM20 and TIM23 following COM treatment, while the levels of autophagy-related proteins showed a clear reduction (Fig. 2I). These results further support the notion of a decrease in mitochondrial autophagy upon silencing PINK1. As PINK1-Parkin-induced mitochondrial dysfunction often accompanies changes in apoptosis, we employed Western blot analysis to examine the markers for apoptosis, including C-Caspase3/Caspase3, Bax, and the anti-apoptotic factor Bcl-2. The observed changes in these proteins indicated a decrease in apoptosis after COM treatment in the silencing PINK1 group when compared to the control group (Fig. 2I). Our findings were further confirmed by flow cytometry analysis, which demonstrated a reduction in apoptotic cells following the silencing of PINK1 and COM treatment (Fig. 2J).

Our research shows that PINK1 plays an essential role in the formation of pediatric kidney stones, possibly by regulating mitochondrial homeostasis to alleviate cellular damage caused by crystal stones.

### Silencing PINK1 alleviates renal calculi in rats by inhibiting mitochondrial oxidative stress

As a classic model of kidney stones, ethylene glycol-induced rat kidney stone formation has been widely used to explore its pathogenesis.

**Fig. 3:**
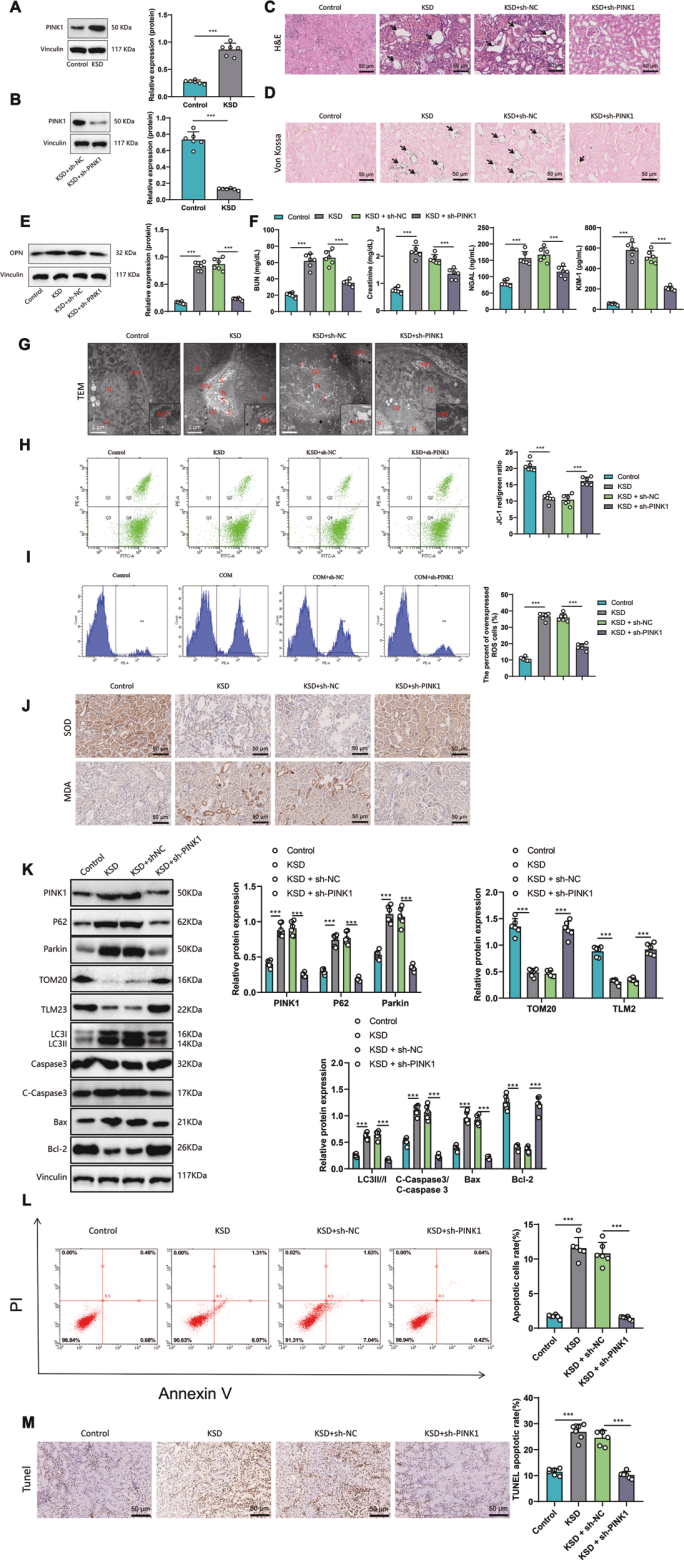
The impact of PINK1 silencing on renal stone rats. **A** Expression of PINK1 in the renal stone rat model. **B** The efficiency of PINK1 silencing was detected after silencing PINK1. **C** Hematoxylin and Eosin (HE) staining results of renal tissues in each group (magnification: 400x), with arrows indicating areas of renal tissue damage. **D** Representative images (magnification: 400x) of rat kidney calcium deposition detected by von Kossa staining, with arrows indicating black substances representing calcium deposits. **E** Expression levels of the renal stone-related protein OPN in each group, as detected by Western blot. **F** Abnormal biochemical index detection of creatinine, blood urea nitrogen, NGAL protein, and KIM-1 protein in each group. **G** Representative images of rat kidney tissue using transmission electron microscopy (scale bar = 10 μm, N indicates nucleus, MV indicates microvilli, LU indicates lumen, MIT indicates mitochondria, S indicates stone trace). **H** Flow cytometry measured δψm levels in each group’s rat kidney tissues. **I** ROS content in rat kidney tissues of each group as measured by flow cytometry. **J** Detection of Detection of Superoxide Dismutase (SOD) and Malondialdehyde (MDA) by immunohistochemistry (magnification: 200x). **K** The changes in PINK1, p62, Parkin, TOM20, TLM2, LC3II/I, and C-Caspase3/Caspase 3, Bax, and Bcl-2 in rat kidney tissues were detected by WB, with the corresponding statistical charts shown on the right side. **L** The apoptotic cell proportion in rat kidney tissues of each group was analyzed by flow cytometry, with the corresponding statistical chart in the first quadrant shown on the right side. **M** Terminal deoxynucleotidyl transferase dUTP nick end labeling (TUNEL) assay was performed to detect the apoptotic cells in rat kidney tissues of each group. Each group consisted of 6 mice. *P < 0.05, **P < 0.01, ***P < 0.001.

In the experiment, we induced a kidney stone model in SD rats using ethylene glycol and ammonium chloride. Western blot results revealed (Fig. 3A, B) a significant increase in PINK1 expression in the kidney tissues of KSD rats compared to the control group. However, after silencing PINK1, there was a notable decrease in PINK1 expression in the kidney tissues of KSD rats, demonstrating a relatively effective knockdown efficiency.

The results of HE staining (Fig. 3C) showed that, compared to the control group, KSD rats exhibited pathological changes such as tubular dilation, destruction, and shedding of renal cells, indicating that ethylene glycol and ammonium chloride could induce severe kidney injury. After silencing PINK1, these pathological changes were alleviated. The von Kossa staining results showed (Fig. 3D) no crystal deposition in the control group, while the oxalate calcium crystals were increased in the model group. After silencing PINK1, the calcium oxalate crystallization in rats decreased. Western blot detection showed increased expression of OPN (Fig. 3E) in the COM group compared to the control group. After silencing PINK1, the expression of OPN decreased. The biochemical indicators of renal function showed increased serum urea nitrogen and creatinine levels in KSD rats compared to the control group (Fig. 3F). The levels of NGAL protein and KIM-1 protein were also elevated in the urine of the rats. After silencing PINK1, urea nitrogen levels, creatinine, NGAL protein, and KIM-1 protein decreased. The results above indicate that the loss of PINK1 can effectively improve the phenotype of kidney stones.

Transmission electron microscopy results (Fig. 3G) show that compared to the control group, the kidneys of KSD rats have thinner renal tubules, dilated tubular lumen, visible crystals in the lumen, swollen mitochondria, and partial collapse of the mitochondrial membrane. After silencing PINK1, the mitochondrial membrane becomes more intact, and the number of cristae in the lumen decreases. The Δψm detection results (Fig. 3H) showed that compared to the control group, the Δψm of the rat kidney tissue in the KSD group decreased. After silencing PINK1, Δψm increased. The ROS content detection results (Fig. 3I) show that compared to the control group, there was a increase in ROS content in the KSD rat tissues, and the ROS content decreased after silencing PINK1.

The immunohistochemical staining results of SOD and MDA (Fig. 3J) showed that, compared with the control group, the expression level of SOD decreased, and the MDA content increased in KSD rats. In addition to the in vitro assays, we further examined indicators related to mitochondrial autophagy and cell apoptosis in different groups of rat kidney tissues (Fig. 3K–M). Compared to the control group, the expression of mitochondrial membrane proteins TOM20 and TLM23 was significantly reduced in the KSD group. Conversely, the expression of PINK1, P62, and Parkin proteins related to mitochondrial autophagy was significantly increased, as well as the expression of apoptosis-related proteins C-Caspase3/Caspase3 and Bax. Conversely, the expression of the apoptosis inhibitor Bcl-2 was significantly decreased, indicating that PINK1 induces increased mitochondrial autophagy and cell apoptosis in the pathological environment of KSD. Silencing PINK1, however, rescued the changes in the above-mentioned proteins induced by the pathological environment of KSD. Specifically, the expression of proteins related to mitochondrial autophagy and apoptosis was decreased, while the expression of the apoptosis inhibitor Bcl-2 was increased. Furthermore, flow cytometry analysis showed a significant increase in the proportion of apoptotic cells in KSD, which was significantly reduced after silencing PINK1. These results suggest that knocking down PINK1 can rescue the increased mitochondrial autophagy and cell apoptosis in KSD, thereby improving rat kidney stone formation.

### MyoD1 may participate in the occurrence of pediatric renal calculi by activating PINK1

**Fig. 4:**
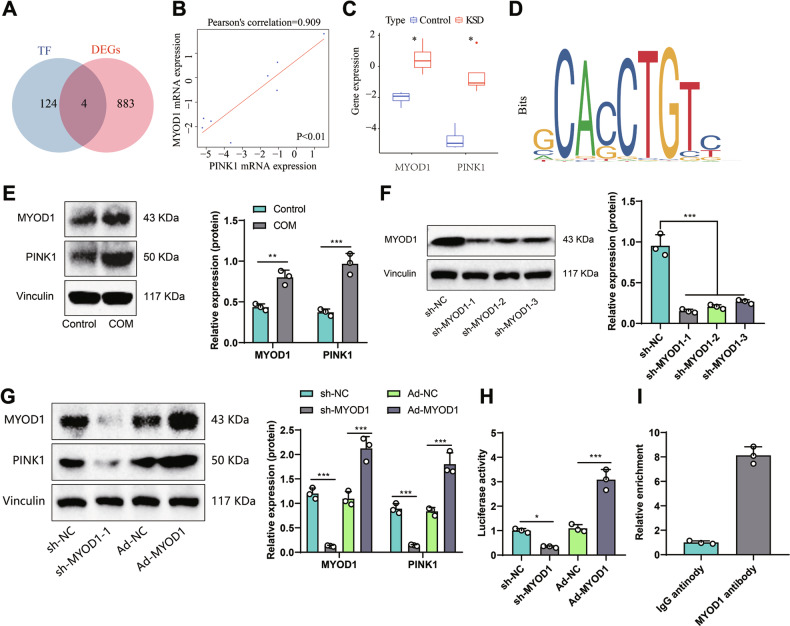
Transcriptional regulation of PINK1 by MyoD1. **A** Venn diagram showing the intersection of transcription factors predicted by hTFtarget and DEGs. **B** Pearson analysis of the correlation between the expression of MyoD1 and PINK1. **C** Expression levels of MyoD1 and PINK1 in peripheral blood of pediatric kidney stone patients in the control group (*n* = 4) and the KSD group (*n* = 4). **D** Identified MyoD1 binding sequence from JASPAR website and predicted binding sites on the PINK1 promoter region. **E** Expression levels of MyoD1 and PINK1 in HK-2 cells of the control and COM groups detected by Western blot. **F** Silencing effect of MyoD1 knockdown detected by Western blot. **G** Expression levels of MyoD1 and PINK1 in cells from different groups detected by Western blot. **H** Luciferase assay detecting the activity of the PINK1 promoter in cells from different groups. **I** ChIP assay detecting the enrichment of MyoD1 on the PINK1 promoter. Note: *P* < 0.05, *P* < 0.01, **P < 0.001. All cell experiments were performed in triplicate.

To study the transcriptional regulatory network of pediatric kidney stones, we obtained 128 transcription factors (TFs) targeting candidate genes through hTFtarget. By analyzing the differential expression in pediatric renal calculi, we identified four differentially expressed genes: CTCFL, MYH11, MyoD1, and PAX5 (Fig. 4A). The correlation analysis results indicate a strong positive correlation between the expression of MyoD1 and PINK1 (Fig. 4B), and they both show an upregulation trend in pediatric kidney stones (Fig. 4C). Literature reports indicate that MyoD1 plays a critical role in muscle development and differentiation and is vital in various diseases such as rhabdomyosarcoma and gastric cancer.

However, the regulatory mechanism of its occurrence in the process of kidney stones has yet to be studied. Therefore, we predicted through the JASPAR website that MyoD1 has binding sites in the promoter region of PINK1 (Fig. 4D). It is hypothesized that MyoD1 could affect the occurrence of kidney stones by activating the transcription of PINK1.

We conducted further Western blot (WB) analysis (Fig. 4E) to validate this hypothesis. In HK-2 cells treated with COM, the expression of MyoD1 and PINK1 both significantly increased, demonstrating a clear positive correlation. To confirm the regulatory role of MyoD1 on PINK1, we performed silencing and overexpression experiments using sh-MyoD1 and Ad-MyoD1, respectively. While ensuring the efficiency of silencing and overexpression, we found that MyoD1 can enhance PINK1 expression, and silencing MyoD1 can inhibit PINK1 expression (Fig. 4F, G). Dual-luciferase assay results indicated a significant decrease in PINK1 promoter activity after silencing MyoD1, and a notable increase in promoter activity after overexpressing MyoD1 (see Fig. 4H). Additionally, Chromatin Immunoprecipitation (ChIP) experiments showed (Fig. 4I), that MyoD1 can be enriched in the PINK1 promoter region and activate PINK1 transcription. These results collectively support our hypothesis that MyoD1 participates in pediatric kidney stone formation by activating PINK1.

### Silencing MyoD1 could inhibit mitochondrial oxidative stress and cellular damage in HK-2 cells by downregulating PINK1 expression

**Fig. 5:**
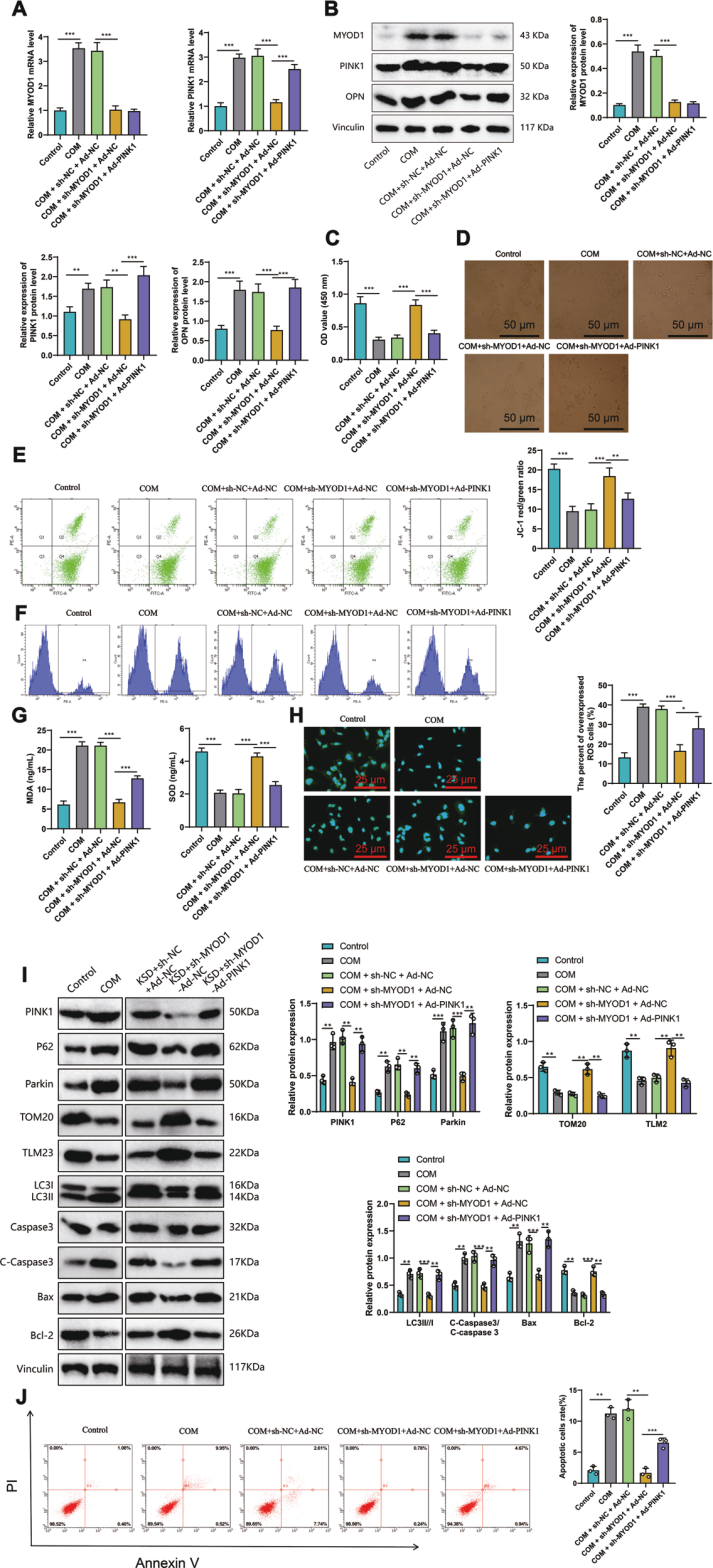
The influence of MyoD1-mediated regulation of PINK1 on mitochondrial oxidative stress and cell damage in HK-2 cells. **A** mRNA expression changes of MyoD1 and PINK1 in different groups detected by RT-qPCR. **B** Expression levels of MyoD1, PINK1, and OPN in different groups detected by Western blot. **C** Cell viability detected by CCK8 in different groups. **D** Representative images (×100) showing the adhesion of COM crystals to HK-2 cell surfaces in different groups. **E** Δψm of HK-2 cells in different groups detected by flow cytometry. **F** ROS levels in HK-2 cells from different groups detected by flow cytometry. **G** Levels of oxidative stress-related factors in HK-2 cells from different groups. **H** Representative images of immunofluorescence detection of mtDNA in each group of cells. **I** WB detection of changes in PINK1, p62, Parkin, TOM20, TLM2, LC3II/I, and C-Caspase3/Caspase 3, Bax and Bcl-2 in each group. **J** Flow cytometry detection of apoptosis in each group of cells, with the first quadrant statistical graph on the right. All cell experiments were repeated 3 times. All cell experiments were repeated three times. *P < 0.05, **P < 0.01, ***P < 0.001.

To investigate whether MyoD1 regulates PINK1 expression and thus affects mitochondrial oxidative stress, we simultaneously silenced MyoD1 and overexpressed PINK1 in HK-2 cells. The RT-qPCR results showed that compared to the control group, the expression of MyoD1 and PINK1 in the COM group was upregulated (Fig. 5A). Compared to the COM+sh-NC+Ad-NC group, the expression of MyoD1 and PINK1 in cells of the COM+sh-MyoD1+Ad-NC group was downregulated. PINK1 expression was upregulated in cells from the COM+sh-MyoD1+Ad-PINK1 group compared to the COM+sh-MyoD1+Ad-NC group. The results demonstrate that under the successful establishment of the kidney stone model, sh-MyoD1 and Ad-PINK1 exhibit highly desirable knockdown and overexpression efficiencies. Furthermore, the overexpression of PINK1 does not affect the expression levels of MyoD1, thus confirming once again the earlier conclusion that MyoD1 acts as an upstream transcription factor regulating PINK1. Western blot detection results showed that compared to the control group (Fig. 5B), MyoD1, PINK1, and OPN expression were upregulated in the COM group. The results indicate that overexpression of PINK1 can reverse the downregulation of PINK1 and the kidney stone-related protein OPN caused by knockdown of MyoD1.

Compared with the COM+sh-NC+Ad-NC group, MyoD1, PINK1, and OPN expression was downregulated in the COM+sh-MyoD1+Ad-NC group cells. However, compared with the COM+sh-MyoD1+Ad-NC group, the expression of PINK1 and OPN was upregulated in the COM+sh-MyoD1+Ad-PINK1 group cells. The CCK8 assay results demonstrated that the viability of HK-2 cells was decreased in the COM group compared to the control group (Fig. 5C).

Compared to the COM+sh-NC+Ad-NC group, the cell activity was increased in the COM+sh-MyoD1+Ad-NC group. However, compared to the COM+sh-MyoD1+Ad-NC group, the cell activity was decreased in the COM+sh-MyoD1+Ad-PINK1 group. The results of the in vitro adhesion experiment showed that, compared with the control group (Fig. 5D), the number of COM crystals adhered to HK-2 cells in the COM group increased. Furthermore, compared with the COM+sh-NC+Ad-NC group, the number of COM crystals in the COM+sh-MyoD1+Ad-NC group decreased, while compared with the COM+sh-MyoD1+Ad-NC group, the number of crystals in the COM+sh-MyoD1+Ad-PINK1 group increased. The Δψm detection results (Fig. 5E) showed that in the control group, the mitochondria maintained a higher membrane potential, while COM caused a decrease in Δψm.

The Δψm is increased in COM+sh-MyoD1+Ad-NC group cells compared to the COM+sh-NC+Ad-NC group but decreased in COM+sh-MyoD1+Ad-PINK1 group cells compared to the COM+sh-MyoD1+Ad-NC group. The ROS content detection results (Fig. 5F) showed that compared with the control group, the ROS content was increased in the COM group. In comparison with the COM+sh-NC+Ad-NC group, the ROS content was decreased in the COM+sh-MyoD1+Ad-NC group, whereas it was increased in the COM+sh-MyoD1+Ad-PINK1 group in comparison with the COM+sh-MyoD1+Ad-NC group. ELISA detection of oxidative stress-related factors showed that compared to the control group (Fig. 5G), the COM group exhibited increased MDA and decreased SOD levels. The results suggest that knocking down MyoD1 can decrease mitochondrial oxidative stress, enhance energy metabolism to maintain cell survival, and overexpression of its downstream target genes can reverse the aforementioned phenotype.

Meanwhile, we also assessed the levels of mitochondrial autophagy and cell apoptosis. We found that the mtDNA content was significantly increased in the COM+sh-MyoD1+Ad-NC group compared to the COM+sh-NC+Ad-NC group (Fig. 5H). Western blot analysis revealed a significant decrease in the expression of mitochondria-autophagy-related proteins PINK1, p62, and Parkin, and a significant increase in the expression of mitochondrial membrane proteins TOM20 and TLM23, as well as a decrease in the expression of apoptosis-related proteins C-Caspase3/Caspase3 and Bax, and an increase in the expression of the anti-apoptotic factor Bcl-2 (Fig. 5I). Flow cytometry analysis showed a significant decrease in the proportion of apoptotic cells (Fig. 5J). In contrast, in the COM+sh-MyoD1+Ad-PINK1 group compared to the COM+sh-MyoD1+Ad-NC group, there was a significant decrease in mtDNA content (Fig. 5H), a significant increase in the expression of mitochondria-autophagy-related proteins PINK1, p62, and Parkin, a significant decrease in the expression of mitochondrial membrane proteins TOM20 and TLM23, a significant increase in the expression of apoptosis-related proteins C-Caspase3/Caspase3 and Bax, and a significant decrease in the expression of the anti-apoptotic factor Bcl-2 (Fig. 5I). Furthermore, flow cytometry analysis showed a significant increase in the proportion of apoptotic cells (Fig. 5J). Comparing to the control group, the COM group exhibited a significant decrease in mtDNA content (Fig. 5H), a significant increase in the expression of mitochondria-autophagy-related proteins PINK1, p62, and Parkin, a significant decrease in the expression of mitochondrial membrane proteins TOM20 and TLM23, a significant increase in the expression of apoptosis-related proteins C-Caspase3/Caspase3 and Bax, and a significant decrease in the expression of the anti-apoptotic factor Bcl-2 (Fig. 5I). Moreover, flow cytometry analysis showed a significant increase in the proportion of apoptotic cells (Fig. 5J). The results indicate that during the process of kidney stone formation, PINK1, as a downstream molecule of MyoD1, can enhance mitochondrial autophagy, thereby promoting cell apoptosis and accelerating the progression of kidney stones.

These results indicate that silencing MyoD1 within HK-2 cells can inhibit PINK1 expression, thereby suppressing mitochondrial oxidative stress, mitochondrial autophagy, and cell apoptosis, leading to reduced cellular damage.

### Silencing MyoD1 could suppress mitochondrial oxidative stress and kidney stone formation by downregulating PINK1 expression in rats with kidney stones

**Fig. 6:**
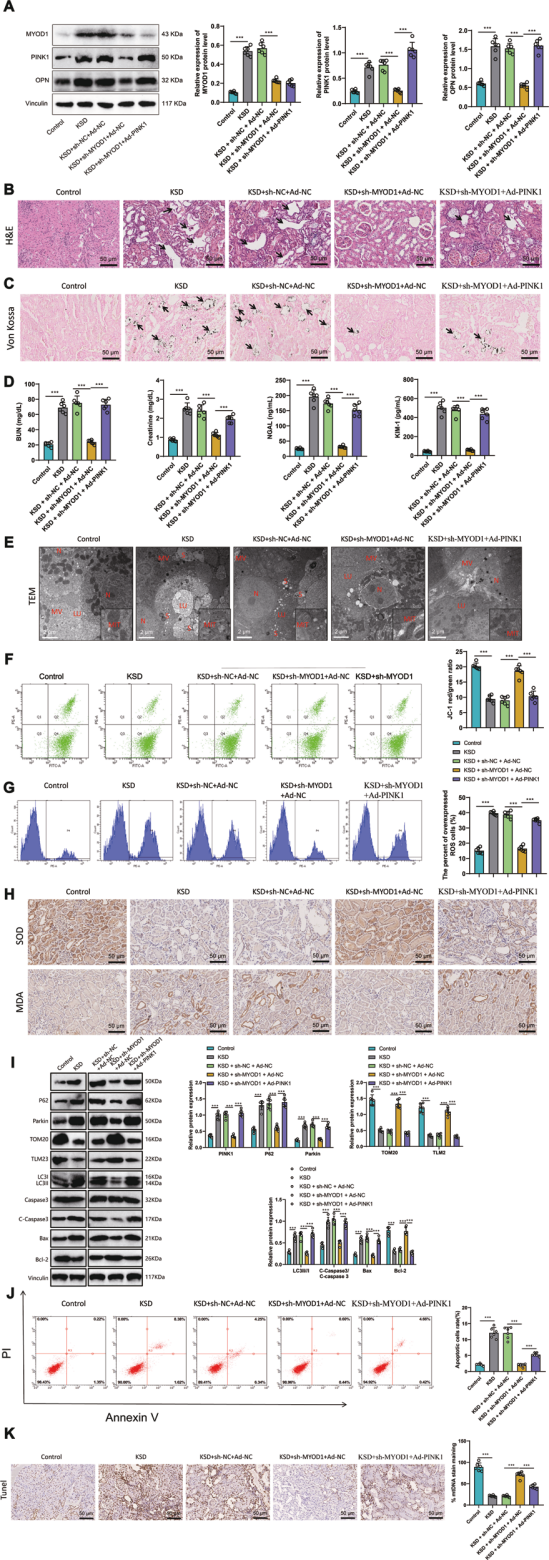
The influence of MyoD1-mediated regulation of PINK1 on mitochondrial oxidative stress and kidney stone formation in rats. **A** Expression levels of MyoD1, PINK1, and OPN in different groups detected by Western blot. **B** Representative HE staining results (×400) indicating the damaged areas in kidney tissues with arrows. **C** Representative images (×400) showing calcium deposition in rat kidneys detected by von Kossa staining with arrows indicating the black substance representing calcium deposits. **D** Biochemical analysis of creatinine, blood urea nitrogen, NGAL protein, and KIM-1 protein levels in different groups. **E** Representative transmission electron microscopy images of rat kidney tissues in different groups (scale bar = 10 μm, N, nucleus; MV, microvillous; LU, lumen; MIT, mitochondria; S, stone remnants). **F** Δψm in kidney tissues from different groups. **G** ROS levels in rat kidney tissues from different groups detected by flow cytometry. **H** Immunohistochemical detection of SOD and MDA levels in rat kidney tissues (×400). **I** WB detected changes in PINK1, p62, Parkin, TOM20, TLM2, LC3II/I, and C-Caspase3/Caspase 3, as well as Bax and Bcl-2 in the renal tissues of each rat group. **J** Flow cytometry was used to detect the percentage of apoptosis in the renal tissues of each rat group. The statistical graph for the first quadrant was shown on the right. **K** TUNEL was used to detect apoptosis in the renal tissues of each rat group. Note: Each group consisted of 6 mice. *P < 0.05, **P < 0.01, ***P < 0.001.

This study utilized a rat model of kidney stones to confirm the inhibitory effect of silencing MyoD1 on PINK1 expression, which could regulate mitochondrial oxidative stress and impact kidney stone formation. Western blot detection results (Fig. 6A) showed that compared to the control group, MyoD1, PINK1, and OPN expression levels were upregulated in the KSD group and compared to the KSD+sh-NC+Ad-NC group, MyoD1, PINK1, and OPN expression levels decreased in the KSD+sh-MyoD1+Ad-NC group. At the same time, compared to the KSD+sh-MyoD1+Ad-NC group, the expression levels of PINK1 and OPN were upregulated in the KSD+sh-MyoD1+Ad-PINK1 group.

The HE staining results (Fig. 6B) showed that compared to the control group, rats in the KSD group exhibited renal tubular dilation, damage, and desquamation. It indicates that ethylene glycol and ammonium chloride severely damage the kidneys. However, compared to the KSD+sh-NC+Ad-NC group, the KSD+sh-MyoD1+Ad-NC group showed a reduction in the severity of renal injury. At the same time, the kidney damage in the KSD+sh-MyoD1+Ad-PINK1 group was even more severe. The von Kossa staining results (Fig. 6C) show no crystal deposition in the control group, while the potassium sodium tartrate (KSD) group increases calcium oxalate crystal deposition. Compared to the KSD+sh-NC+Ad-NC group, the KSD+sh-MyoD1+Ad-NC group reduced intrarenal crystal deposition.

However, compared to the KSD+sh-NC+Ad-NC group, the KSD+sh-MyoD1+Ad-PINK1 group showed increased crystal deposition in the kidneys. The results of renal function biochemical index detection (Fig. 6D) showed that compared to the control group, the levels of blood urea nitrogen and creatinine in the serum of rats in the KSD group increased, and the levels of NGAL and KIM-1 in the urine also increased. However, compared to the KSD+sh-NC+Ad-NC group, the levels of blood urea nitrogen and creatinine in the serum of rats in the KSD+sh-MyoD1+Ad-NC group were reduced, and the content of NGAL and KIM-1 in the urine was also decreased. The results demonstrate that the absence of MyoD1 and PINK1 can effectively ameliorate the phenotype of kidney injury.

However, compared to the KSD+sh-NC+Ad-NC group, the levels of blood urea nitrogen and creatinine in the serum of rats in the KSD+sh-MyoD1+Ad-PINK1 group increased, and the content of NGAL and KIM-1 in urine also increased. Transmission electron microscopy results (Fig. 6E) showed that compared to the control group, the renal tubules in the KSD group of rats were thinner, the tubular lumen was dilated, crystals were visible inside the lumen, mitochondria were swollen, and parts of the mitochondrial membranes collapsed. However, compared to the KSD+sh-NC+Ad-NC group, the mitochondrial membrane was more intact, and the luminal crystals were reduced in the KSD+sh-MyoD1+Ad-NC group. The results indicate that the absence of MyoD1 and PINK1 is of significant importance for maintaining the integrity of mitochondrial membrane structure during the process of kidney stone formation.

And compared to the KSD+sh-NC+Ad-NC group, the KSD+sh-MyoD1+Ad-PINK1 group exhibited more severe mitochondrial swelling and membrane collapse. The Δψm detection results (Fig. 6F) show that compared to the control group, the KSD group has a decrease in Δψm. However, compared to the KSD+sh-NC+Ad-NC group, the Δψm content in the KSD+sh-MyoD1+Ad-NC group increased. Compared to the KSD+sh-NC+Ad-NC group, the Δψm content decreased in the KSD+sh-MyoD1+Ad-PINK1 group. The ROS content detection results (Fig. 6G) showed that compared to the control group; the KSD group had a increase in ROS content.

However, compared to the KSD+sh-NC+Ad-NC group, the KSD+sh-MyoD1+Ad-NC group showed a decrease in ROS content. Compared to the KSD+sh-NC+Ad-NC group, the ROS content increased in the KSD+sh-MyoD1+Ad-PINK1 group. Immunohistochemical detection of SOD and MDA showed that, compared to the control group, the expression level of SOD decreased, and the content of MDA increased in KSD group rats (Fig. 6H). Compared with the KSD+sh-NC+Ad-NC group, the KSD+sh-MyoD1+Ad-NC group exhibited elevated renal SOD expression levels and reduced MDA content.

In the study, we also examined the levels of mitochondrial autophagy and cell apoptosis. We observed significant decreases in the expression of mitochondrial autophagy-related proteins PINK1, p62, and Parkin in the KSD+sh-MyoD1+Ad-NC group compared to the KSD+sh-NC+Ad-NC group. Additionally, the expression of mitochondrial membrane proteins TOM20 and TLM23 significantly increased, while the expression of apoptosis-related proteins C-Caspase3/Caspase3 and Bax decreased, and the expression of the apoptosis inhibitor Bcl-2 increased (Fig. 6I). Furthermore, the proportion of apoptotic cells detected by flow cytometry and TUNEL assay significantly decreased (Fig. 6J, K). In contrast, in the KSD+sh-MyoD1+Ad-PINK1 group compared to the KSD+sh-MyoD1+Ad-NC group, we observed significant increases in the expression of PINK1, p62, and Parkin, decreases in the expression of TOM20 and TLM23, increases in the expression of C-Caspase3/Caspase3 and Bax, and decreases in the expression of Bcl-2 (Fig. 6I). Additionally, the proportion of apoptotic cells detected by flow cytometry and TUNEL assay significantly increased (Fig. 6J, K) in this group. Compared to the control group, the KSD group exhibited significant increases in the expression of PINK1, p62, and Parkin, decreases in the expression of TOM20 and TLM23, increases in the expression of C-Caspase3/Caspase3 and Bax, and decreases in the expression of Bcl-2 (Fig. 6I). Furthermore, the proportion of apoptotic cells detected by flow cytometry and TUNEL assay significantly increased (Fig. 6J, K) in the KSD group. However, compared to the KSD+sh-NC+Ad-NC group, the KSD+sh-MyoD1+Ad-PINK1 group exhibited a decrease in renal SOD expression levels, a significant increase in MDA content, enhanced mitochondrial autophagy, and increased cell apoptosis. This suggests that downregulating MyoD1 can inhibit PINK1 expression, thereby preserving the integrity of mitochondrial membrane structure, suppressing mitochondrial oxidative stress and autophagy, subsequently reducing cell apoptosis and the formation of subsequent kidney stones according to the experimental findings.

## Discussion

This study explores the molecular mechanism of MyoD1-mediated mitochondrial homeostasis regulation in the induction of pediatric kidney stone formation through the transcriptional activation of PINK1 expression. It is an innovative study in the field. Previous research has mainly focused on studying the environmental and genetic factors involved in the formation of kidney stones, while relatively little is known about how specific genes or proteins affect the mechanism of kidney stone formation [23]. Although the functions of MyoD1 and PINK1 have been extensively studied in other diseases, their roles in pediatric kidney stones still need to be clarified. By incorporating the studies of MyoD1 and PINK1 into the field of kidney stones, this research provides a new perspective for understanding the pathogenesis of kidney stones. We found that PINK1 is highly expressed in pediatric nephrolithiasis patients, consistent with some earlier research findings that revealed abnormal expression of PINK1 in various diseases [24]. However, there are currently no conclusive research results regarding the specific role of PINK1 in pediatric kidney stones. Through experimental verification, we found that the silence of PINK1 could inhibit the formation of kidney stones inside and outside the body, indicating that PINK1 plays an essential role in the formation process of pediatric kidney stones.

Another important finding of this study is that MyoD1 is an upstream transcription factor of PINK1, which could participate in developing pediatric kidney stones by activating PINK1. This point has yet to be mentioned in previous studies. Although it is known that MyoD1 could regulate the expression of many genes, this is the first time that it has been found to activate the expression of PINK1 and affect the formation of pediatric kidney stones [25–28]. This study provides us with new clues for understanding the pathogenesis of kidney stones.

**Fig. 7:**
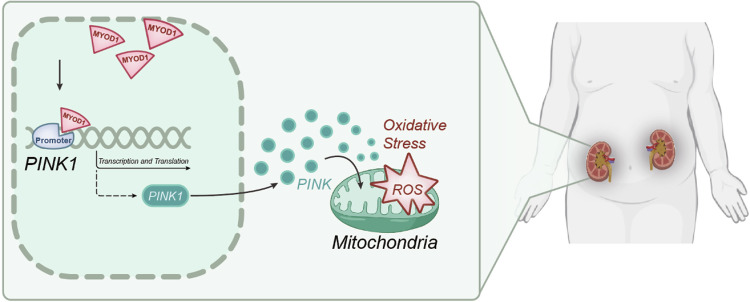
Schematic of the molecular mechanism by which MyoD1 activates the transcription of PINK1 to promote the occurrence of pediatric kidney stones.

Our in vivo and in vitro experiments confirm that silencing MyoD1 could inhibit mitochondrial oxidative stress and kidney stone formation in rats by downregulating the expression of PINK1. This finding strengthens the vital role of MyoD1 in regulating PINK1 expression and mediating pediatric kidney stone formation through mitochondrial oxidative stress. Although MyoD1 has been shown to have the ability to influence mitochondrial function in other diseases, this ability has not been reported in the formation process of kidney stones [29]. In summary, this study preliminarily concludes the following: MyoD1 may promote the occurrence and development of kidney stones through transcriptional activation of PINK1 expression, which in turn promotes mitochondrial oxidative stress (Fig. 7).

This study reveals for the first time that MyoD1 may regulate mitochondrial homeostasis and induce pediatric kidney stone formation by transcriptionally activating the expression of PINK1, shedding light on the possible mechanism behind it. We found that silencing MyoD1 could inhibit the formation of kidney stones by downregulating PINK1 expression in both in vitro and in vivo kidney stone models, providing new insights into the pathogenesis of pediatric nephrolithiasis. In addition, our findings also provide potential targets for developing new strategies to treat pediatric kidney stones, such as developing drugs that could inhibit the expression of MyoD1 or PINK1. Overall, this study is valuable for scientific research and holds potential for clinical applications. This study provides insights into the potential roles of MyoD1 and PINK1 in pediatric nephrolithiasis, offering a novel perspective for understanding the pathogenesis of this disease. In the future, we will further investigate the dynamic expression and function of MyoD1 and PINK1 in pediatric nephrolithiasis, aiming to reveal their more detailed molecular mechanisms in the disease progression. At the same time, we will validate our findings in a larger clinical sample to evaluate whether MyoD1 and PINK1 could serve as potential targets for treating pediatric kidney stones. Finally, we will also attempt to develop drugs that could suppress the expression of MyoD1 or PINK1, providing new strategies for treating pediatric kidney stones.

## Materials and Methods

### Clinical sample collection

This study collected peripheral blood samples from 4 pediatric patients with renal calculi and 4 without kidney disease or metabolic abnormalities. The sampling method is venous blood extraction, with 3 mL of peripheral blood collected for each sample and 1 mL of anticoagulant added for preservation. All participants or their legal guardians have signed informed consent forms, and this study has obtained approval from ethics committee of Anhui Provincial Children’s Hospital by the relevant requirements of the Helsinki Declaration.

### Access to high-throughput transcriptome sequencing data set of clinical samples

Clinical samples should be transported immediately to the laboratory for sample processing and RNA extraction. Use Trizol reagent (ThermoFisher, 16096020, New York, USA) to extract total RNA from each sample according to the manufacturer’s instructions. The concentration, purity, and integrity of the RNA were measured using the Qubit® 2.0 Fluorometer® (Life Technologies, California, USA) with the Qubit® RNA Analysis Kit (HKR2106-01, Shanghai Baoji Bioscience Co., Ltd.), the Nanometer spectrophotometer (IMPLEN, California, USA), and the RNA Nano 6000 Analysis Kit (Agilent, 5067-1511) on the Bioanalyzer 2100 system. The total RNA content of each sample is 3 μg, which serves as the input material for RNA sample preparation. Use NEBNext®UltraTM RNA Library Prep Kit (NEB, E7435L, Beijing, compatible with Illumina®, Nebraska, USA) to prepare the cDNA library as recommended by the manufacturer and evaluate the quality on Agilent Bioanalyzer 2100 system. The TruSeq PE Cluster Kit v3 cBot HS (Illumina, PE-401-3001) for Illumina is used for clustering indexed samples. After cluster formation, the library preparation is sequenced using the Illumina-Hiseq 550 platform [30].

### High-throughput sequencing data quality control

Use FastQC software version v0.11.8 to check the quality of paired-end reads in the raw sequencing data. Preprocess the raw data using Cutadapt software version 1.18 to remove Illumina sequencing adapters and poly(A) tail sequences. Remove reads with an N percentage exceeding 5% using a Perl script. Next, use the FASTX Toolkit software version 0.0.13 to extract reads with base quality above 20 and retain only 70% of the bases. Repair the paired-end sequences using BBMap software. Finally, the filtered high-quality reads were aligned to the human reference genome using hisat2 software version 0.7.12 [31, 32].

### Bioinformatics analysis

The “Limma” package in R software was utilized to identify differentially expressed genes (DEGs) between normal control samples and kidney stone samples, with adjusted P values being calculated using the Benjamini–Hochberg false discovery rate (FDR). Genes meeting the criteria of |log2FC | > 2 and *P*-value < 0.05 were considered as DEGs. Next, query the keyword “mitochondrial oxidative stress” on the Genecards website to obtain 200 genes related to mitochondrial oxidative stress. Then use the Xiantao Academic website to find the intersection. Protein-protein interaction analysis was performed by importing the selected genes into the STRING database (https://string-db.org/). After limiting the species to humans, use the “count” package in R language to sort the nodes in the PPI network and perform analysis based on the Degree value [33].

### Preparation of calcium oxalate monohydrate COM

Add 10 mM CaCl_2_ and 1 mM sodium oxalate Na_2_C_2_O_4_ to equal volumes of 90 mM NaCl in 10 mM Tris-HCl buffer (pH 7.4). Mix the CaCl_2_ solution with the NaOx solution, resulting in a final concentration of 5 mM CaCl_2_ and 0.5 mM Na_2_C_2_O_4_. After incubating overnight, centrifuge at 3000 g for 10 min at room temperature. Collect COM crystals and wash them twice with methanol. Finally, air dry at 37 °C [34].

### Construction and grouping of in vitro cell models of renal calculi

The renal tubular epithelial cell line HK-2 was purchased from ATCC (USA, CRL-2190). In DMEM/F-12 medium (USA, ThermoFisher, 11320033), add penicillin (100 U/mL), streptomycin (100 μg/mL), and 10% FBS to culture under conditions of 5% CO_2_ and 37 °C. The cells were divided into the following groups: control group, COM group, COM+sh-NC group (infected with negative control adenovirus sh-NC), COM+sh-PINK1 group (infected with adenovirus sh-PINK1), COM+sh-NC+Ad-NC group (infected with negative control adenovirus sh-NC and Ad-NC), COM+sh-MyoD1+Ad-NC group (infected with adenovirus sh-MyoD1 and negative control adenovirus Ad-NC), COM+sh-MyoD1+Ad-PINK1 group (infected with adenovirus sh-MyoD1 and adenovirus Ad-PINK1). Add 1 × 10^7^ corresponding adenoviruses to all groups of HK-2 except for the control group and incubate in DMEM/F-12 medium containing 100 μg/mL COM crystals for 24 h after 48 h of cultivation [35].

### Construction and grouping of the rat model of kidney stones

Male SD rats at eight weeks of age were purchased from Beijing Vitonlihua Experimental Animal Technology Co., Ltd. in Beijing, China and were housed under non-pathogenic conditions at a temperature of 26–28 °C and humidity of 50–65%. All animal experiments comply with the ethical standards approved by our Animal Ethics Committee. The animals were randomly divided into the following groups: Control group, KSD group, KSD+sh-NC group (infected with sh-NC negative control adenovirus), KSD+sh-PINK1 group (infected with sh-PINK1 adenovirus), KSD+sh-NC+Ad-NC group (infected with sh-NC negative control adenovirus and Ad-NC negative control adenovirus), KSD+sh-MyoD1+Ad-NC group (infected with sh-MyoD1 adenovirus and Ad-NC negative control adenovirus), and KSD+sh-MyoD1+Ad-PINK1 group (infected with sh-MyoD1 adenovirus and Ad-PINK1 adenovirus), with six rats in each group. Adenovirus was purchased from Shanghai GenePharma Co., Ltd. The mice in the control and KSD groups are divided according to experimental needs. The KSD group of rats was subjected to daily gavage with 1% (w/v) ammonium chloride 1 ml and given 1% (v/v) ethylene glycol (EG) water to drink for three weeks. The KSD rats were divided into the following groups: sh-NC, sh-PINK1, sh-NC+Ad-NC, sh-MyoD1+Ad-NC, and sh-MyoD1+Ad-PINK1. After an intravenous injection of 1 mL of adenovirus daily, all rats in the Experimental group were administered 1% (w/v) physiological saline, 1 mL, by oral gavage daily. Other groups of rats were treated with 1% (v/v) ethylene glycol (EG) in water daily, and 1% (w/v) ammonium chloride 1 ml was added to the drinking water for three weeks. After three weeks, all rats were euthanized under ether anesthesia, and their kidneys were immediately dissected for analysis. Each rat kidney was fixed in 4% paraformaldehyde, dehydrated in ethanol, embedded in paraffin, and prepared as continuous sections of 5 μm. Staining with hematoxylin-eosin (HE) and von Kossa, CaOx crystals were observed under a polarized light microscope. Another kidney was used for RNA and protein extraction. All procedures in the experiment adhere to the National Institutes of Health Guide for the Care and Use of Laboratory Animals [30].

### Real-Time quantitative Polymerase Chain Reaction (RT-qPCR)

Total RNA was extracted from cells using TRIzol (15596026, ThermoFisher, USA), and the purity and concentration of RNA were assessed using a nanodrop2000 spectrophotometer (ThermoFisher, USA). According to the instructions of the PrimeScript RT Reagent Kit (Takara Code: RR047A, Takara, Japan), mRNA is reverse transcribed into cDNA. TaKaRa Corporation synthesized all the gene primers (Table S1). Real-time fluorescence quantitative PCR detection was performed using the 7500 Fast system (4351106, ThermoFisher, USA). The reaction conditions were as follows: pre-denaturation at 95 °C for 10 min, denaturation at 95 °C for 10 s, annealing at 60 °C for 20 s, extension at 72 °C for 34 s, and a total of 40 cycles. Using GAPDH as an internal reference, relative quantification (${2}^{{-\Delta\Delta}{\rm{CT}}}$ method) is used to calculate the relative transcription levels: $\Delta\Delta{\rm{Ct}}$ = $\Delta$Ct experimental group - $\Delta$ Ct control group, $\Delta$ Ct = Ct (target gene) - Ct (internal reference), the relative transcription level of the target gene mRNA = ${2}^{{-\Delta\Delta}{\rm{ct}}}$ [36]. The experiment was repeated three times.

### Adenovirus infection

After purchasing the adenovirus overexpression vector AdEasy (24009, Agilent Technologies, USA) and the adenovirus interference vector (76977, China Haijihaoge Biotechnology Co., Ltd.), we constructed an adenovirus-based MyoD1 silencing vector as well as a PINK1 overexpression or silencing vector. Please refer to Table S2 for the silent sequence. We co-transfect the packaged virus with the target vector into HEK293T cells (CRL-3216, ATCC, USA), and after 48 h, collect the cell culture supernatant to obtain an adenovirus titer of 1 × 10^8^ TU/ml [37, 38].

### Western blot

Extract total proteins from tissues using RIPA lysis buffer containing PMSF (P0013C, Biyuntian, Shanghai, China). Incubate the sample on ice for 30 min, centrifuge at 8000 g for 10 min at 4 °C, and collect the supernatant. Detect the total protein concentration using the BCA assay kit (23227, ThermoFisher, USA). Take 50 μg of protein and dissolve it in 2x SDS loading buffer, boil for 5 minu, then perform SDS-PAGE gel electrophoresis on the samples. Transfer the proteins onto a PVDF membrane and then block with 5% skim milk powder at room temperature for 1 h. The PVDF membrane was subsequently incubated overnight at 4 °C with a dilution of primary antibodies against skeletal muscle differentiation protein (MyoD1, rabbit, ab307805, 1:1000), Pink1 protein (PINK1, mouse, ab186303, 1:1000), osteopontin protein (OPN, rabbit, ab63856, 1:1000), mitochondrial fusion protein 2 (MFN2, rabbit, ab124773, 1:5000), mitochondrial fission protein 1 (DNM1L, rabbit, ab184247, 1:1000), LC3II/I (rabbit, 12741 S, 1:1000), TOM20 (rabbit, 42406 S, 1:1000), TLM23 (rabbit, 11123-1-AP, 1:1000), Caspase3 (rabbit, 9664 S, 1:1000), p62 (rabbit, 18420-1-AP, 1:1000), Parkin (mouse, from Santa Cruz Biotechnology, sc-32282, 1:1000), Bax (rabbit, ab32503, 1:1000), Bcl-2 (rabbit, ab194583, 1:1000), and Vinculin (rabbit, ab219649, 1:10000) as internal controls. The membrane was then washed three times for 10 min each with TBST.

Incubate the membrane with the secondary antibody goat anti-rabbit IgG H&L (HRP) labeled with horseradish peroxidase (ab97051, 1:2000, Abcam, Cambridge, UK) and the secondary antibody rabbit anti-mouse IgG H&L (HRP) (ab6728, 1:2000, Abcam, Cambridge, UK) for 1 h. After washing with TBST, place the membrane on a clean glass plate. Add equal amounts of ECL fluorescent detection reagents A and B onto the membrane after mixing them well in a darkroom. Using the Quantity One v4.6.2 image analysis system from Bio-Rad, images were captured, and the relative protein content of corresponding protein bands was represented by the brightness value/Vinculin protein band intensity value [39]. Each experiment is repeated three times, and the average value is taken. Please refer to the Supplementary information for Full length uncropped original western blots.

### Cell Counting Kit-8 (CCK-8)

The study utilized the CCK-8 assay kit (CA1210, Beijing Solaibao Technology Co., Ltd., Beijing, China) to conduct experiments on cell proliferation and analyze the results. In the experiment, logarithmic growth phase cells were seeded in 96-well plates at a density of 1 × 10^4^ cells per well. After pre-culturing for 24 h, cell transfection was performed according to the grouping. After transfection for 48 h, add 10 μL of CCK-8 reagent and incubate at 37 °C for 3 h. Finally, measure the absorbance values of each well on an ELISA reader, detecting the values at a wavelength of 450 nm. The value could be directly proportional to the number of cell proliferation in the culture medium [40]-finally, drawbar charts of the activity levels for each group of cells.

### In vitro adhesion assay

Transfer the pre-treated HK-2 cells to a 6-well plate at a density of 100,000 cells per well. After incubation in serum-free medium for 6 h, crushed calcium oxide crystals (100 μg/mL) were added to the 6-well plate and incubated for 30 min. Then, remove the original culture medium, rinse the cells with PBS three times for 5 min each to remove unbound crystals, and observe the adhesion of crystals to the surface of HK-2 cells under a light microscope [41].

### Hematoxylin and Eosin (HE) staining

Unfold the kidney tissue slices and dewax them in water. According to the instructions provided by Shanghai Bogu Biological Technology Co., Ltd., PT001, purchased from Shanghai, China, is used for HE staining kidney tissue sections. The main steps are as follows: Sudan IV staining at room temperature for 10 min, followed by rinsing with running water for 30–60 s; differentiation with 1% hydrochloric acid alcohol for 30 s, followed by rinsing with running water and soaking in water for 5 min; counterstaining with eosin at room temperature for 1 minute; dehydration with alcohol gradients (concentrations of 70%, 80%, 90%, 95%, and 100%), each gradient for 1 minute; treatment with xylene and ammonium carbonate for 1 minute, followed by two steps of clarification in xylene I and II, each for 1 min; finally, the sections were sealed with neutral mounting medium and observed for morphological changes of different renal tissues under an optical microscope (BX50; Olympus Corp, Tokyo, Japan) [42, 43].

### Von Kossa staining was used to detect CaOx deposits in rat kidneys

After removing the paraffin from the paraffin-embedded slices, soak them in 2% silver nitrate for 20–60 min, then rinse with distilled water for five min. Next, treat the slices with a 5% sodium thiosulfate solution for two min, and rinse them under tap water for five min. Finally, use 0.1% hematoxylin to counterstain the slices for one to two min, rinse for 5 to 10 s, then dehydration, cleaning, and sealing with neutral gum. To observe the crystallization of CaOx, five random areas were selected from each renal cortex section in tissue slices stained with Von-Kossa [30, 44].

### Transmission Electron Microscope (TEM)

The method for observing the microscopic structure of mitochondria in the kidney using a transmission electron microscope is as follows: Firstly, perfuse the kidney with 20 ml of 0.1 M phosphate buffer and 20 ml of 2.5% glutaraldehyde to complete fixation. Then, wash with phosphate buffer. Next, fix it in 2% osmium tetroxide for 2 h, then dehydrate it using graded ethanol (50–100%). Embed the organization in epoxy resin and polymerized for 48 h at 60 °C. Finally, uranium and lead double-staining was performed on the ultra-thin sections (99 nm), and they were observed using a JEM-1011 TEM microscope (JEOL Ltd., Tokyo, Japan) [45].

### TUNEL staining

The Terminal deoxynucleotidyl transferase dUTP nick-end labeling (TUNEL) staining kit (Roch, China) was used to detect cell apoptosis in the renal tissues of each rat group. After removing the paraffin-embedded slices, the tissue sections were incubated with proteinase K (2 mg/ml) for 10 min and then covered with 100 μl of 1 × Equilibration Buffer to fully cover the target areas. The sections were incubated at room temperature for 10–30 min. Simultaneously, the dUTP Labeling Mix was thawed on ice, excess Equilibration Buffer was removed, and 50 μl of TdT incubation buffer was added to the tissues to avoid exposure to light. The sections were then incubated at 37 °C for 60 min, observed and photographed under a microscope. Five random areas were selected in each renal cortex section [46].

### Biochemical parameter detection of serum and urine samples

Collect the whole blood samples from the rats after the experiment ends, let them stand at 37 °C for 1 h, and centrifuge at 4000 rpm for 8 min. Subsequently, the serum was taken for renal function testing. SCr and BUN tests were conducted using the ADVIA 1800 biochemical analyzer from Siemens, Germany. One day before the end of the experiment, we collected 24-h fasting urine samples from the rats using metabolic cages. The levels of urinary NGAL and KIM-1 were measured using ELISA kits provided by Chinese manufacturer FineTest (ER0003) and Jiangsu Jianglai (FT-PD6252S), respectively [34, 47].

### Flow cytometry

We used specific assay kits for flow cytometry to detect changes in mitochondrial membrane potential (Δψm) and the generation of reactive oxygen species (ROS). For Δψm, we used a mitochondrial membrane potential detection kit (C2006, Beyotime, China) and JC-1 solution to incubate the cells and perform flow cytometry analysis. To detect the generation of ROS, we followed the instructions of the ROS detection kit (S0033M, Beyotime, China) and incubated the cells with 10 µmol/L of 2’,7’-dichlorodihydrofluorescein diacetate (DCFH-DA) for 20 min. After that, the cells were washed with serum-free DMEM/F-12, and the fluorescence intensity under 488 nm excitation and 525 nm emission was analyzed by flow cytometry. We dissected and isolated the kidneys in the animal experiment and prepared a 10% homogenate. Furthermore, we incubate the cells in the dark with 10 µM DCFH-DA for 15 min. It is worth noting that cellular esterases oxidize the non-fluorescent dye into a fluorescent product called DCF, which is primarily used for detecting ROS, especially hydrogen peroxide (H_2_O_2_) levels. Finally, we recorded the average fluorescence intensity (MFI) of DCF-DA using a flow cytometer (BD FACScanto II) [33, 48, 49]. In order to detect cellular apoptosis, the Annexin V-FITC/PI apoptosis detection kit (E-CK-A211, Elabscience) was utilized to assess the occurrence of apoptosis in both cells and renal tissues. The treated cells and digested single-cell renal tissues were incubated with Annexin V-APC and propidium iodide (PI) in a dark chamber at room temperature for 15 min. Analysis was performed using the FACScan flow cytometry system (Becton Dickinson). The resulting data revealed that the top left quadrant represented necrotic cells, the top right quadrant represented cells in late-stage apoptosis, the bottom right quadrant represented cells in early-stage apoptosis, and the bottom left quadrant represented cells that had not undergone apoptosis. The mortality rate in this study was calculated as the sum of the early-stage and late-stage mortality rates [50].

### Detection of SOD and MDA activity

Harvest the supernatants from each cell group, then strictly follow the instructions of the ELISA kit for experimental procedures to detect the levels of MDA and SOD activity in the cell culture medium. The ELISA reagents used in the experiment were: human MDA (JL11466, Shanghai Jianglai Industrial Co., Ltd., Shanghai, China) and human SOD (JL14005, Shanghai Jianglai Biotechnology Co., Ltd., Shanghai, China) [51].

### Immunohistochemical testing

For the immunohistochemical detection of SOD activity and MDA content in SD rats, tissue sections embedded in paraffin (with a thickness of 4.0 μm) were dewaxed, dehydrated, and rehydrated in a graded ethanol series. To inhibit endogenous peroxidase activity, add 3% H2O2. Subsequently, the slices were rinsed with distilled water and phosphate-buffered saline (PBS), placed in citrate buffer (10 mM, pH 6.0), and heated in a microwave oven at 95 °C for 30 min. Subsequently, before overnight treatment at 4 °C, anti-rat SOD and MDA antibodies (SODsc-101523, Santa Cruz Biotechnology, MDAab79055, Abcam, Cambridge, UK) were utilized. According to the manufacturer’s instructions, the Vectastain Elite ABC kit (Vector Laboratories, Burlingame, CA, USA) was used to detect positive reactions [45].

### Dual luciferase assay

The PCR amplification was used to amplify the PINK1 promoter sequence with the MyoD1 binding site and then cloned it into the pGL3 (Promega, Madison) vector to generate the pGL3-PINK1 plasmid. The plasmid pGL3-PINK1 was co-transfected with oe-NC, oe-MyoD1, sh-NC, and sh-MyoD1 into human embryonic kidney cells HEK293T (iCell-h237, Sibenco Biotech Co., Ltd., Shanghai, China) respectively, to evaluate the impact of MyoD1 on PINK1 promoter activity. The activity of the internal reference Renilla luciferase is used to standardize the activity of the luciferase reporter gene. Cells were collected and lysed 48 h after transfection, and the luciferase activity was measured using the Luciferase Assay Kit (K801-200, BioVision, USA). The luciferase reporter gene detection uses the dual luciferase reporter gene analysis system (Promega, Madison, WI, USA). To determine the changes in PINK1 promoter activity, we compared the luciferase reporter gene activation levels among different transfection groups by calculating the ratio of relative light units (RLU) obtained from firefly luciferase assay to those obtained from renilla luciferase assay [52, 53].

### Chromatin immunoprecipitation (ChIP)

Cells should be fixed in 1% formaldehyde for 10 min and terminated with 0.125 M glycine. After washing, scrape off the cells and collect the precipitate by centrifugation. Then resuspend the cells in cell lysis buffer (20 mM Tris-HCL, pH 8.0, 85 mM KCL, 0.5% NP40, and protease inhibitor) and centrifuge to collect the cell nuclei. Translate the sentence into English: Disrupt the nuclear pellet in SDS lysis buffer (1% SDS, 10 mM EDTA, 50 mM Tris-HCL, pH 8.1, and protease inhibitors), followed by sonication to shear DNA to a size range of 200 bp to 1000 bp. Then perform ChIP using diluted sonication buffer. Incubated with anti-MyoD1 antibody (ab307805, 1:1000, Abcam, Cambridge, UK) and anti-IgG antibody overnight at 4 °C, with IgG antibody as the negative control.

The Pierce Protein A/G magnetic beads (88803, Thermo Fisher Scientific, USA) could bind DNA with MyoD1 through centrifugal precipitation. Then the precipitate was centrifuged at 12,000 × g for 5 min, non-specific complexes were washed away from the residue, and the cross-linked DNA fragments were recovered and purified as amplification templates overnight at 65 °C. Detecting immunoprecipitated PINK1 was performed using iQ SYBR Green Supermix (BioRad) via RT-qPCR. Forward primer sequence of PINK1 promoter: TGTGTGTTCTGTGGTGAGCA; reverse primer sequence: TTTCCCTTTGCACTTTGGAC [54–56]. The experiment was repeated three times.

### Mitochondrial DNA (mtDNA) Immunostaining

Cell were seeded on Lab Tek cell chamber slides and treated with the corresponding COM. The treated cells were then rinsed in PBS and fixed at room temperature with 4% paraformaldehyde for 15 min. After fixation, the cells were treated with 0.1% Triton X-100 for 10 min at room temperature, followed by blocking with 3% goat serum for 40 min. Subsequently, the cells were incubated overnight at 4 °C with diluted DNA antibody (Progen Biotechnik) in 3% goat serum. After washing with PBS, the cells were incubated for 1 h at room temperature with secondary antibodies, either anti-rabbit or anti-mouse Alexa Fluor-488 and 633 conjugated antibodies (Life Technologies). The cells were washed three times with 1% Triton X-100 PBS, each time for 5 min. For the final wash, the cells were incubated with DAPI (10 μg/mL, Sigma) in PBS for 5 min. Mitochondrial autophagy was assessed by immunostaining of mitochondrial DNA (mtDNA). Images were acquired from DAPI-stained samples using an LSM 510 confocal microscope (Zeiss) equipped with a Plan-Apochromat 63×/1.4 oil DIC objective for immunostaining of DNA. Four image slices encompassing the top and bottom of the cells were collected in the Z-plane. All images obtained from the Z-plane were subjected to image analysis using Volocity software (Perkin-Elmer v6.0.1). The remaining mtDNA staining percentage was calculated using the formula: (cDNAv - nDNAv) / n, where cDNAv represents the total cellular DNA volume determined by staining with an anti-DNA antibody, nDNAv represents the total nuclear DNA volume determined by DAPI staining, and n is the number of cells. The mtDNA staining volume in control cells was normalized to 100% [57].

### Statistical analysis

All data were analyzed using SPSS 22.0 statistical software (SPSS, Inc., Chicago, IL, USA) and processed with GraphPad Prism 9.5. Measurement data are represented by mean ± standard deviation (Mean ± SD), and the comparison between the two groups is performed using an unpaired t-test. Multiple-group comparisons should be conducted using a one-way analysis of variance. Use the Levene test to test the homogeneity of variances; if the variances are homogenous, use Dunnett’s t and LSD-t tests to compare between two groups, where *P* < 0.05 indicates that the difference is statistically significant. If the variances are not homogeneous, use Dunnett’s T3 test.

## Supplementary information

Supplementary information

## Data Availability

The datasets generated and/or analyzed during the current study are available from the corresponding author on reasonable request.
