# Supplementary material for: Transcriptional activation of PINK1 by MyoD1 mediates mitochondrial homeostasis to induce renal calcification in pediatric nephrolithiasis
Source: Cell Death Discov. 2024 Sep 6;10:397. doi: 10.1038/s41420-024-02117-w (PMC11379875; doi:10.1038/s41420-024-02117-w)
Supplement: Supplementary file 1 — Supplementary information [file 41420_2024_2117_MOESM1_ESM.docx]

**Table S1. RT-qPCR primer sequences**

| Gene | primer sequences (5'-3') |
| --- | --- |
| PINK1 (human) | Forword：5'-CCTGGAGTGTGAAACGCTCT-3'  Reverse：5'-CTCCCACCCTCACCATTCAC-3' |
| MFN2 (human) | Forword：5'-GAGGCCGGGAAGGTGAAGTC-3'  Reverse：5'-AGAGCAGGGACATTGCGTTTT-3' |
| DNM1L (human) | Forword：5'-TCACCCGGAGACCTCTCATT-3' |
|  | Reverse：5'-GTTTTTCCATGTAGCAGGGTCA-3' |
| MYOD1 (human) | Forword：5'-CAGAGCTGAACCTTGAGGGG-3' |
|  | Reverse：5'-TGGGTTACGGTTACACCTGC-3' |
| GAPDH (human) | Forword：5'-AAAGCCTGCCGGTGACTAAC-3' |
|  | Reverse：5'-TTCCCGTTCTCAGCCTTGAC-3' |

**Table S2. shRNAsequence**

| Name | shRNA sequence |
| --- | --- |
| shRNA-NC | Sense 5’-UAAGGCUAUGAAGAGAUACdTdT-3’ |
|  | Antisense 5’-GUAUCUCUUCAUAGCCUUAdTdT-3’ |
| shRNA-PINK1-1 (human) | Sense 5’- TCCCAAGGTGCTGTGATTATAdTdT-3’ |
|  | Antisense 5’-TATAATCACAGCACCTTGGGAdTdT-3’ |
| shRNA-PINK1-2 (human) | Sense 5’- CCTACTCCTACCTACATTAAAdTdT-3’ |
|  | Antisense 5’-TTTAATGTAGGTAGGAGTAGGdTdT-3’ |
| shRNA-PINK1-3 (human) | Sense 5’- GAAGGAACTTGTGCCAAATTAdTdT-3’ |
|  | Antisense 5’-TAATTTGGCACAAGTTCCTTCdTdT-3’ |
| shRNA-MYOD1-1 (human) | Sense 5’- CCCTCCCAACAGCGCTTTAAA dTdT-3’ |
|  | Antisense 5’-TTTAAAGCGCTGTTGGGAGGGdTdT-3’ |
| shRNA-MYOD1-2 (human) | Sense 5’- CAGGGAATTTGTACGTTTATAdTdT-3’ |
|  | Antisense 5’-CAGGGAATTTGTACGTTTATAdTdT-3’ |
| shRNA-MYOD1-3 (human) | Sense 5’-GCCGCCTGAGCAAAGTAAATGdTdT-3’ |
|  | Antisense 5’-CATTTACTTTGCTCAGGCGGCdTdT-3’ |
| shRNA-PINK1-1 (rat) | Sense 5’-AGTAAACTGTACAGGAAATTAdTdT-3’ |
|  | Antisense 5’-TAATTTCCTGTACAGTTTACTdTdT-3’ |
| shRNA-PINK1-2 (rat) | Sense 5’-GTAAACTGTACAGGAAATTAAdTdT-3’ |
|  | Antisense 5’-TTAATTTCCTGTACAGTTTACdTdT-3’ |
| shRNA-PINK1-3 (rat) | Sense 5’-GTCAGGAGATCCAGGCAATTTdTdT-3’ |
|  | Antisense 5’-AAATTGCCTGGATCTCCTGACdTdT-3’ |
| shRNA-MYOD1-1 (rat) | Sense 5’-ATGGTTCAGGACCACTTATTTdTdT-3’ |
|  | Antisense 5’-AAATAAGTGGTCCTGAACCATdTdT-3’ |
| shRNA-MYOD1-2 (rat) | Sense 5’-TCGCTCAGGTGTTGGAAATAAdTdT-3’ |
|  | Antisense 5’-TTATTTCCAACACCTGAGCGAdTdT-3’ |
| shRNA-MYOD1-3 (rat) | Sense 5’-CGCTCAGGTGTTGGAAATAAAdTdT-3’ |
|  | Antisense 5’-TTTATTTCCAACACCTGAGCGdTdT-3’ |

Full length uncropped original western blots


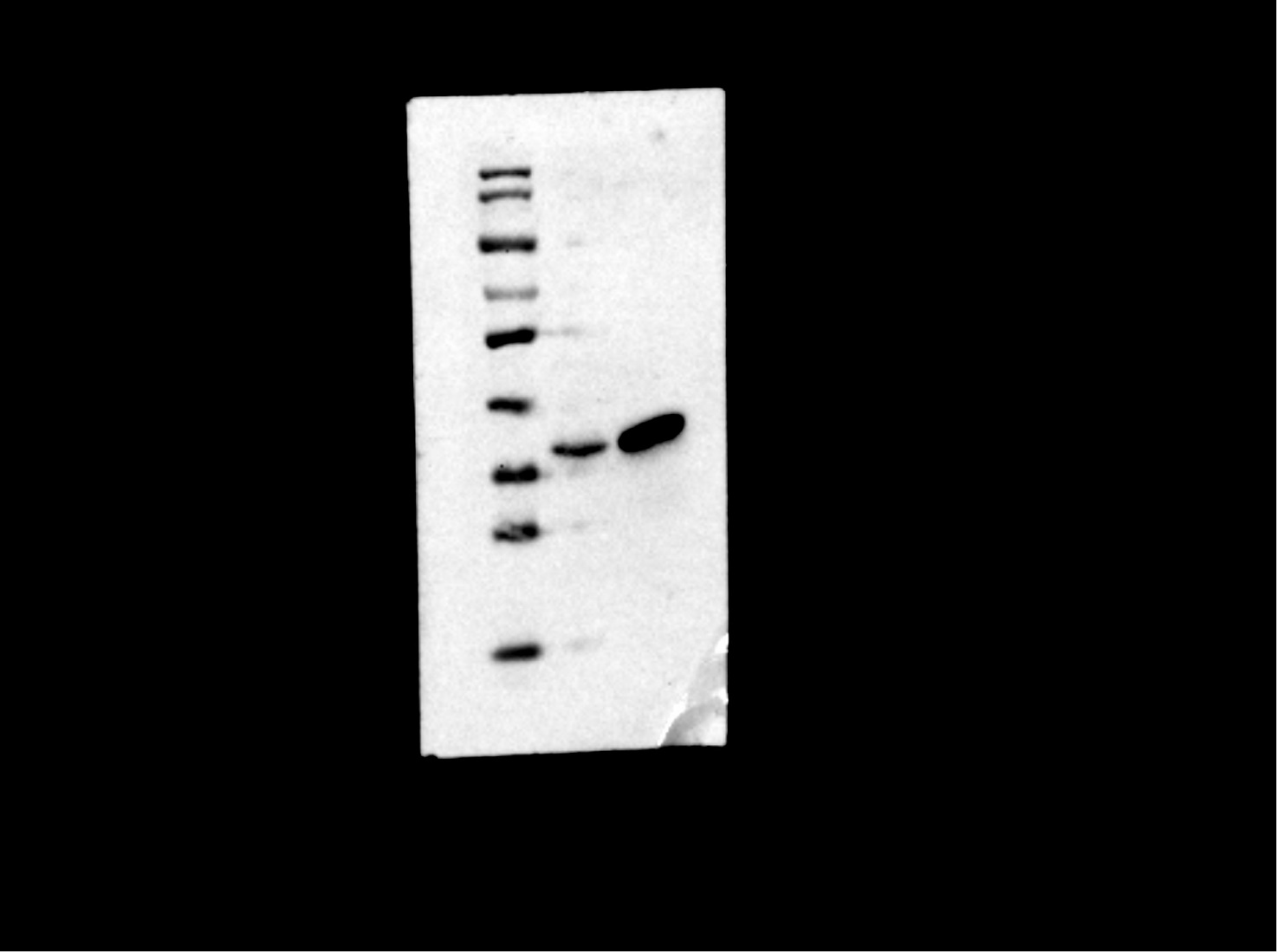


Figure 1H-1


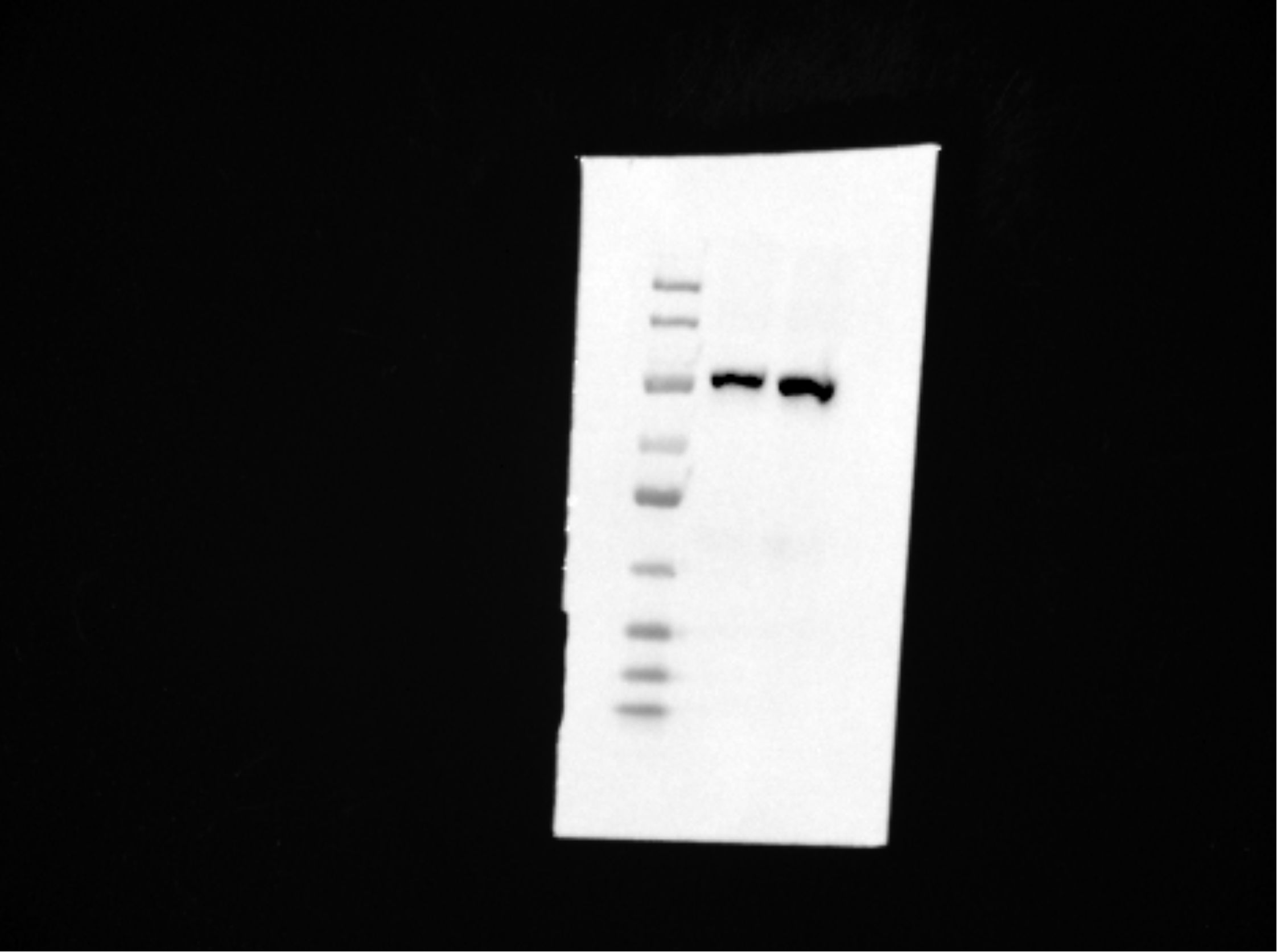


Figure 1H-2


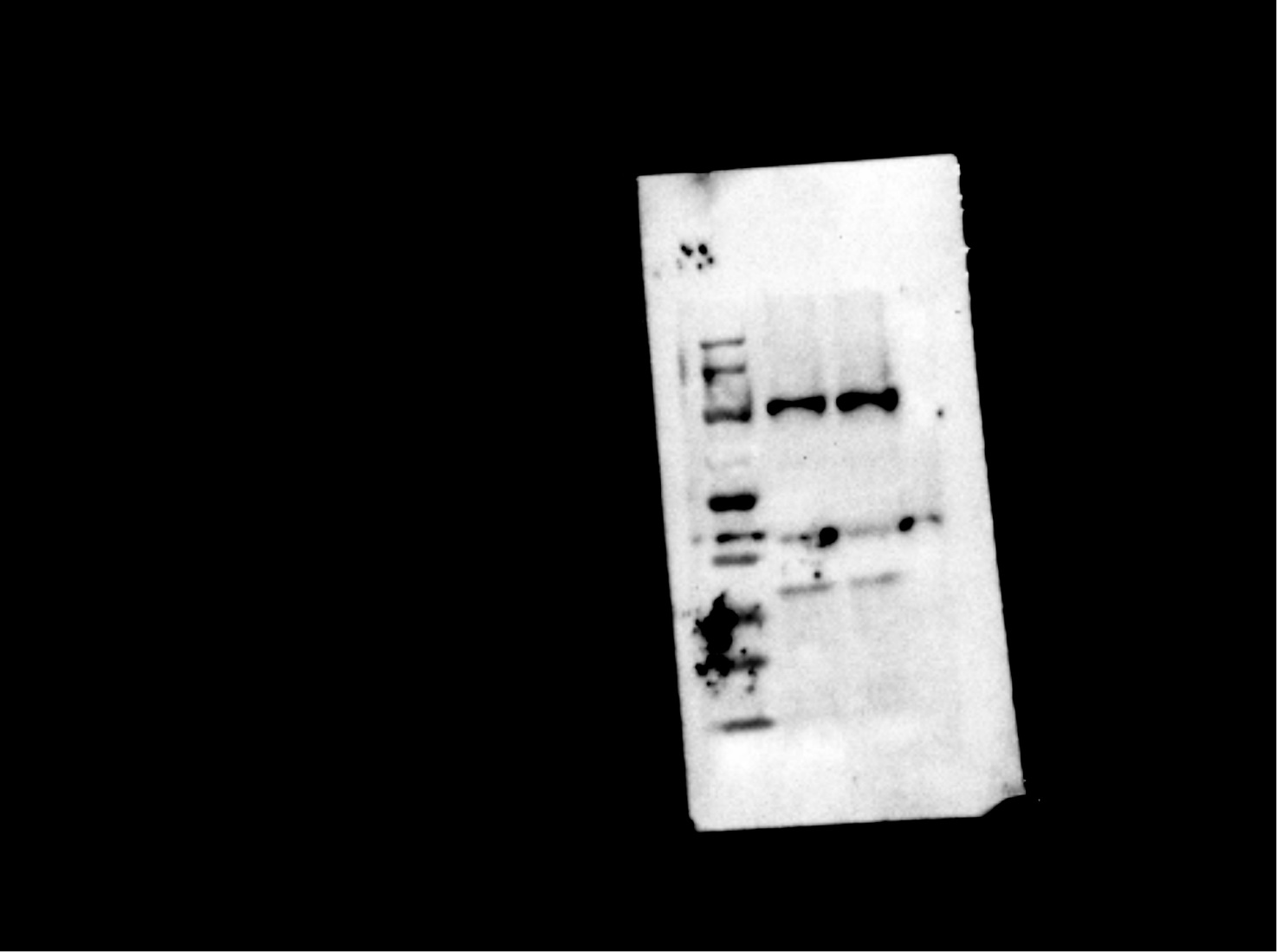


Figure 1H-3


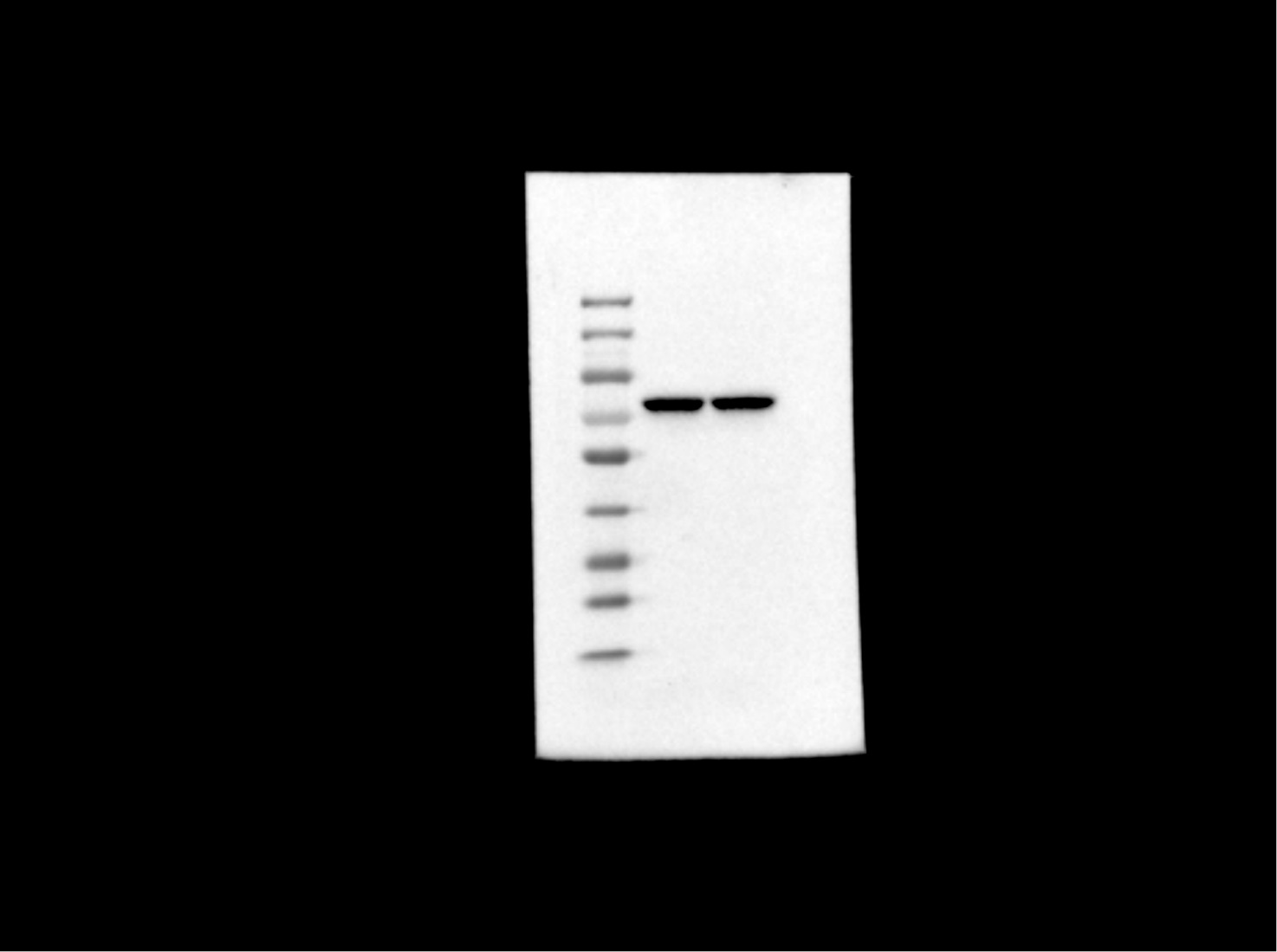


Figure 1H-4


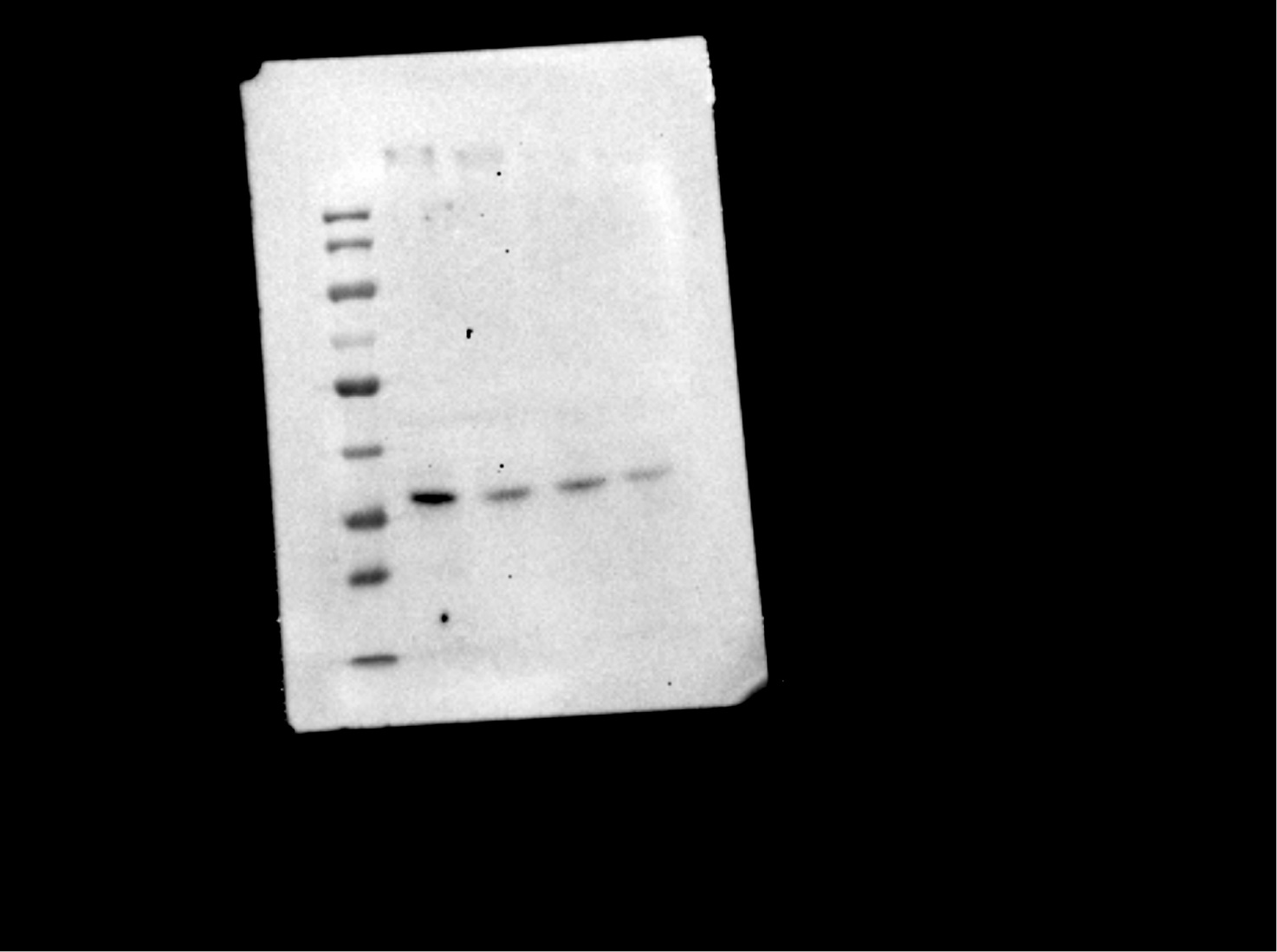


Figure 2A-1


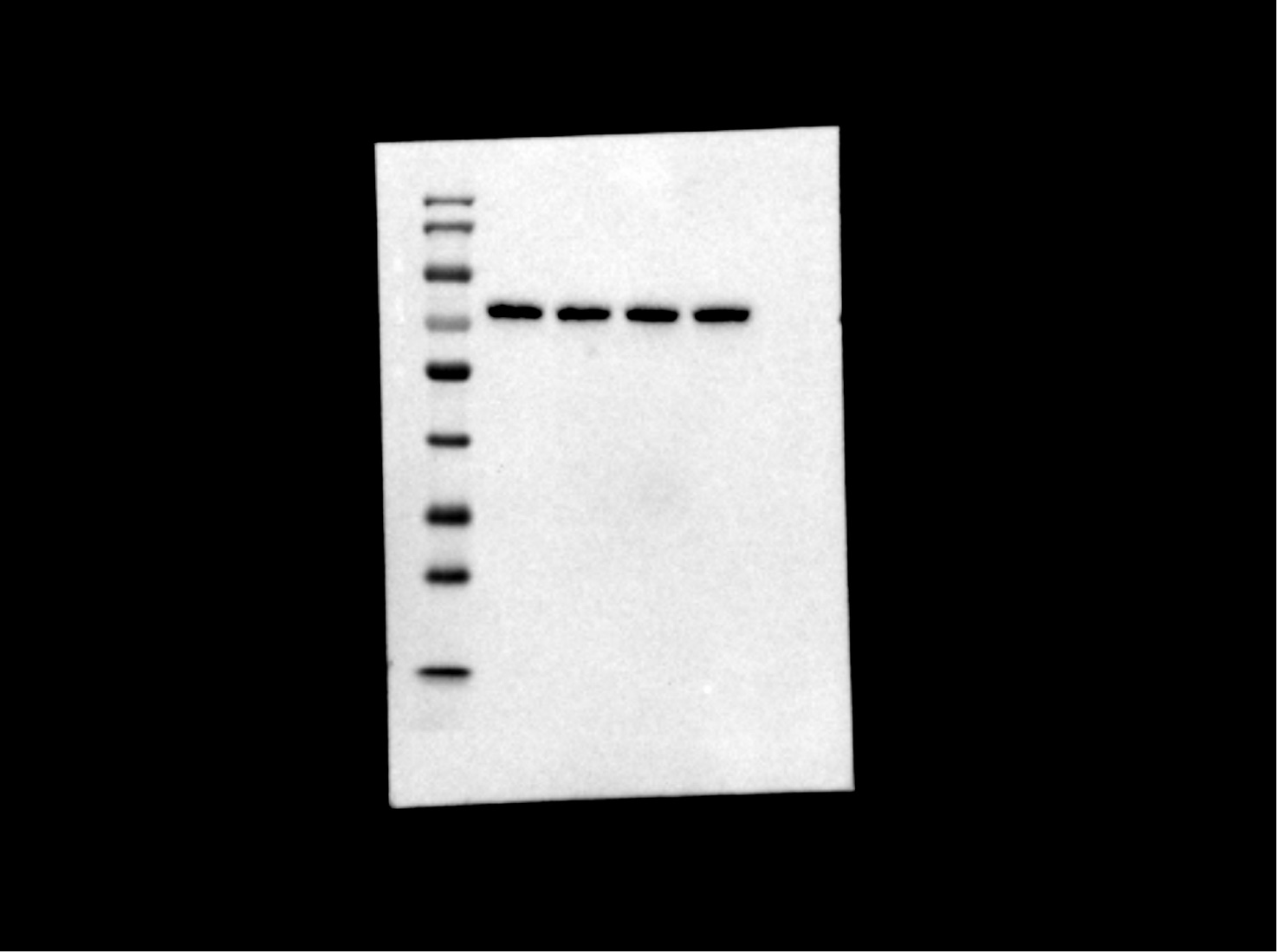


Figure 2A-2


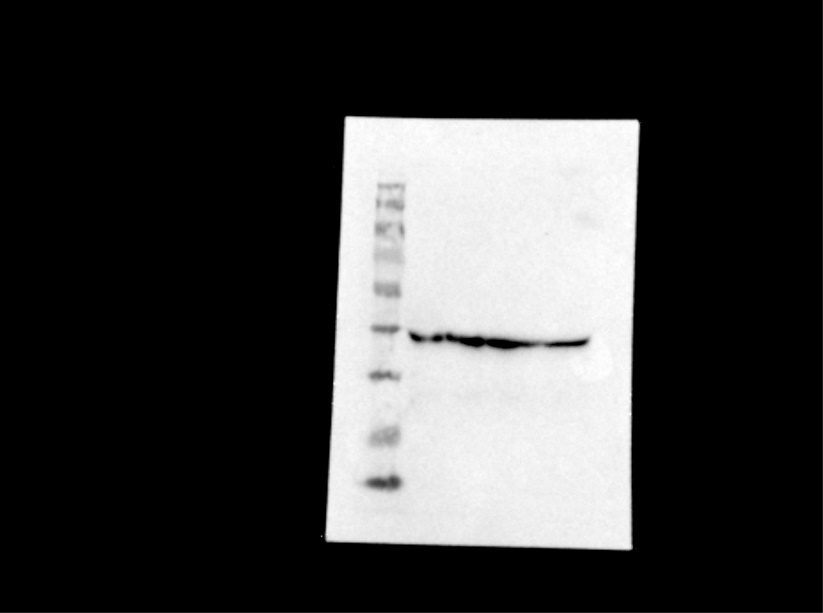


Figure 2I-1


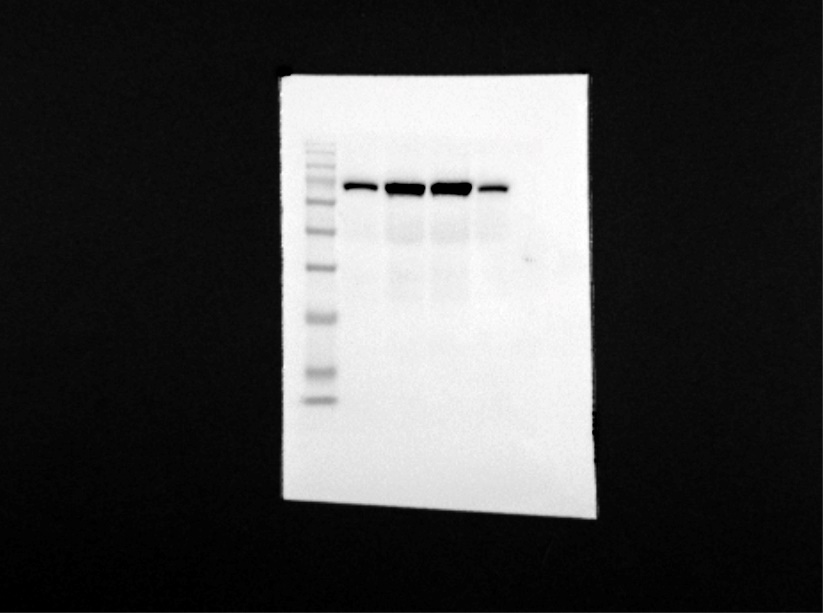


Figure 2I-2


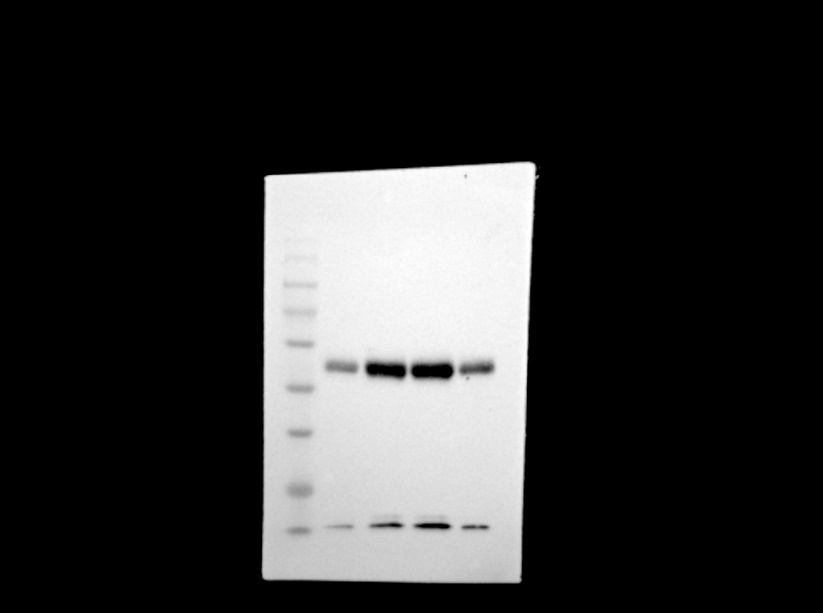


Figure 2I-3


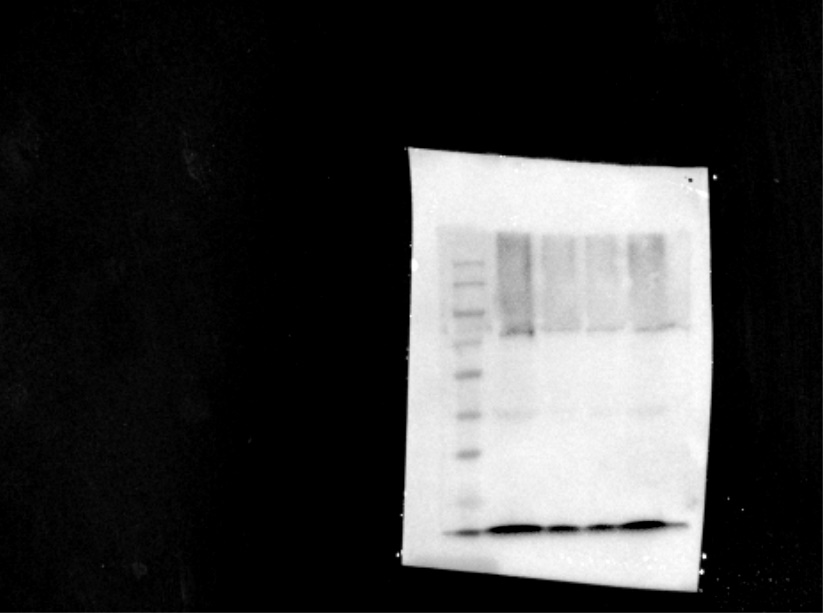


Figure 2I-4


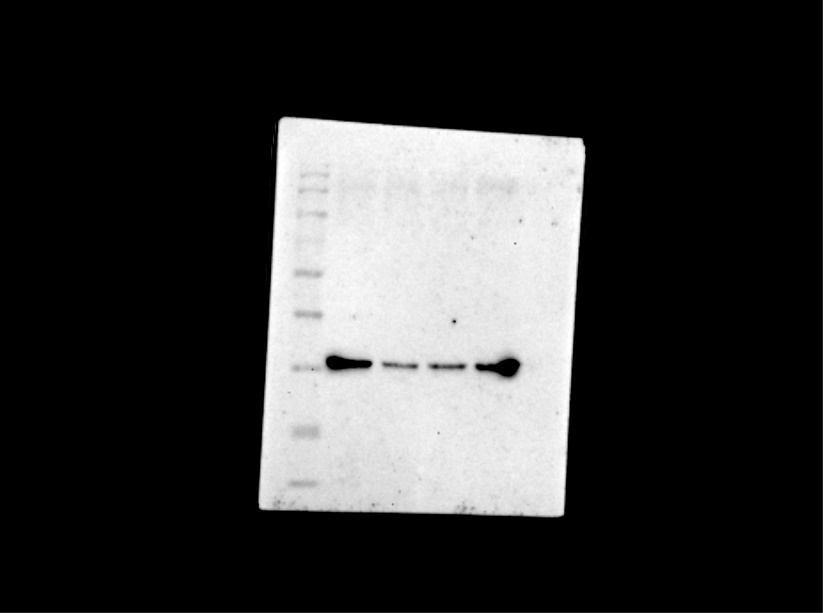


Figure 2I-5


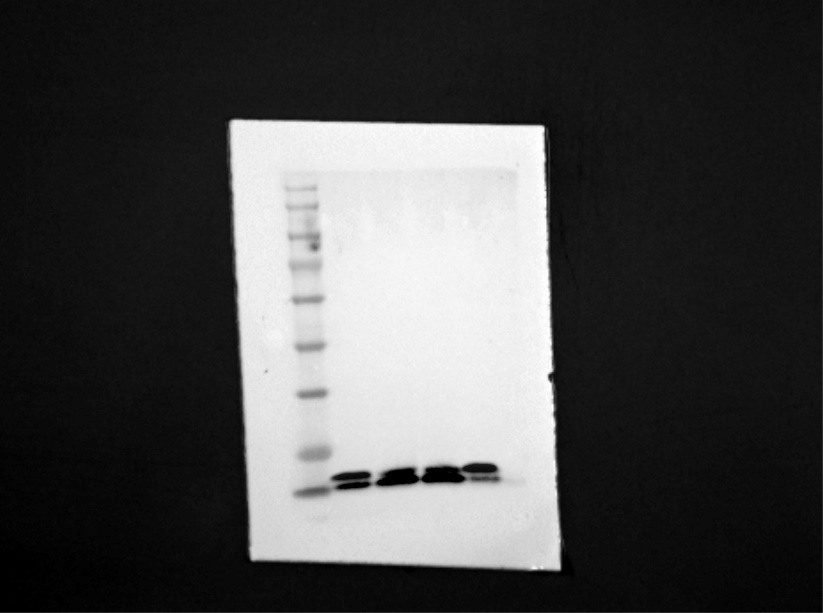


Figure 2I-6


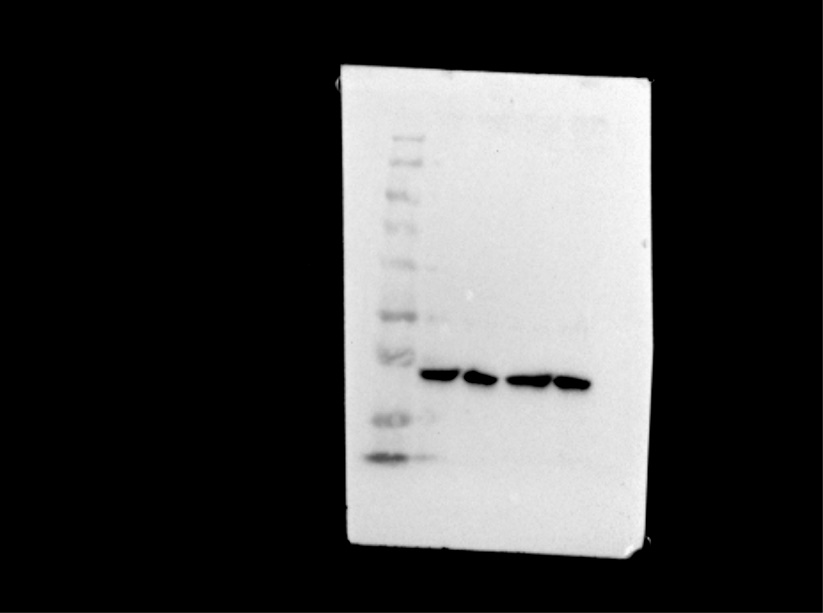


Figure 2I-7


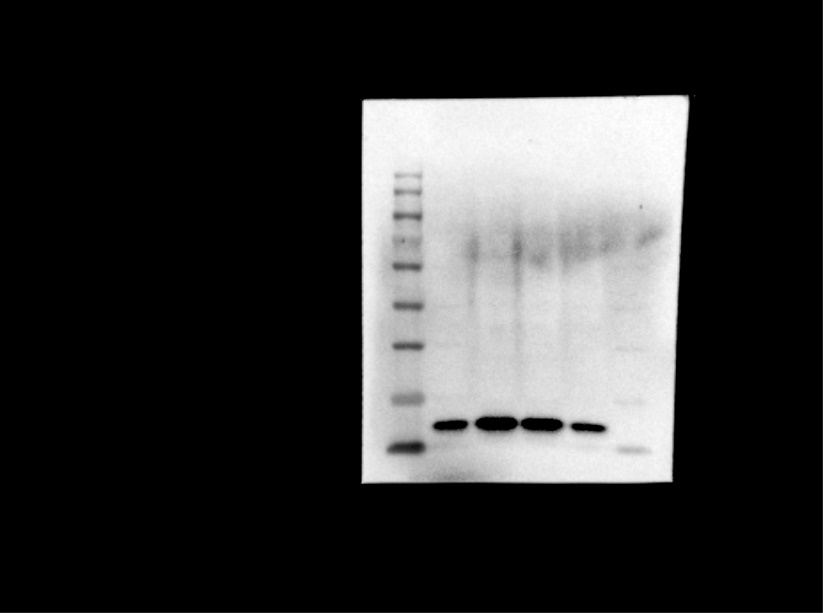


Figure 2I-8


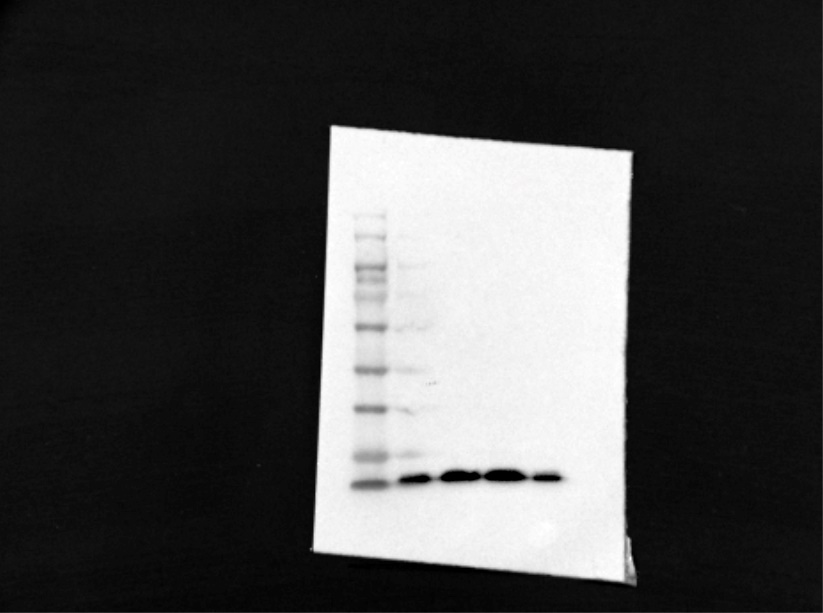


Figure 2I-9


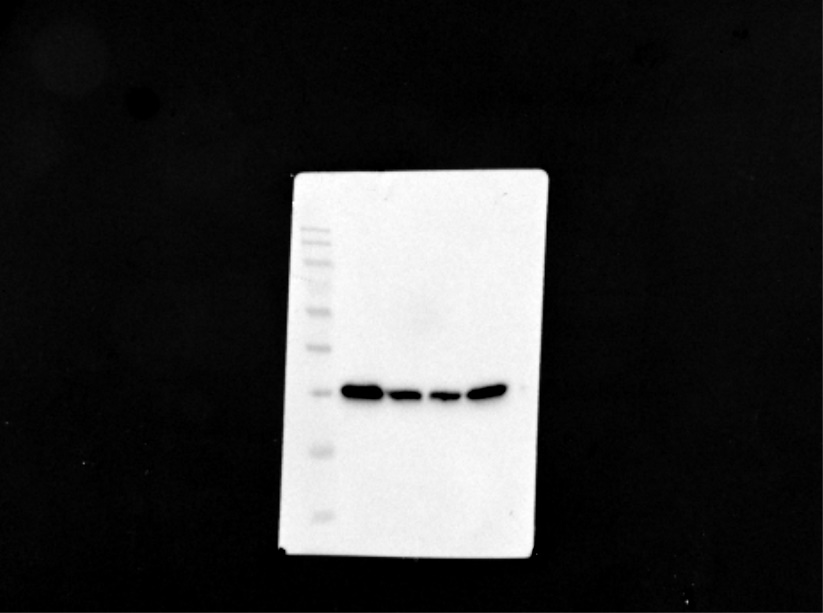


Figure 2I-10


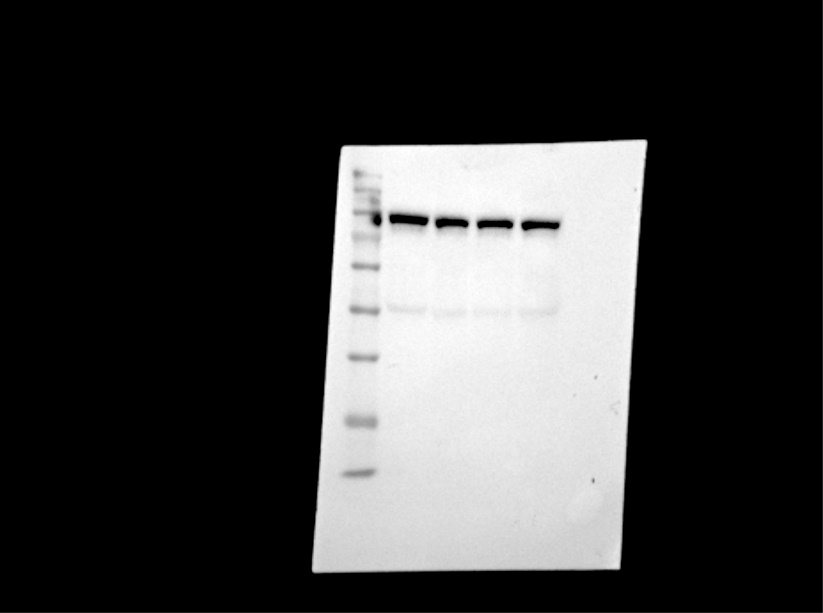


Figure 2I-11


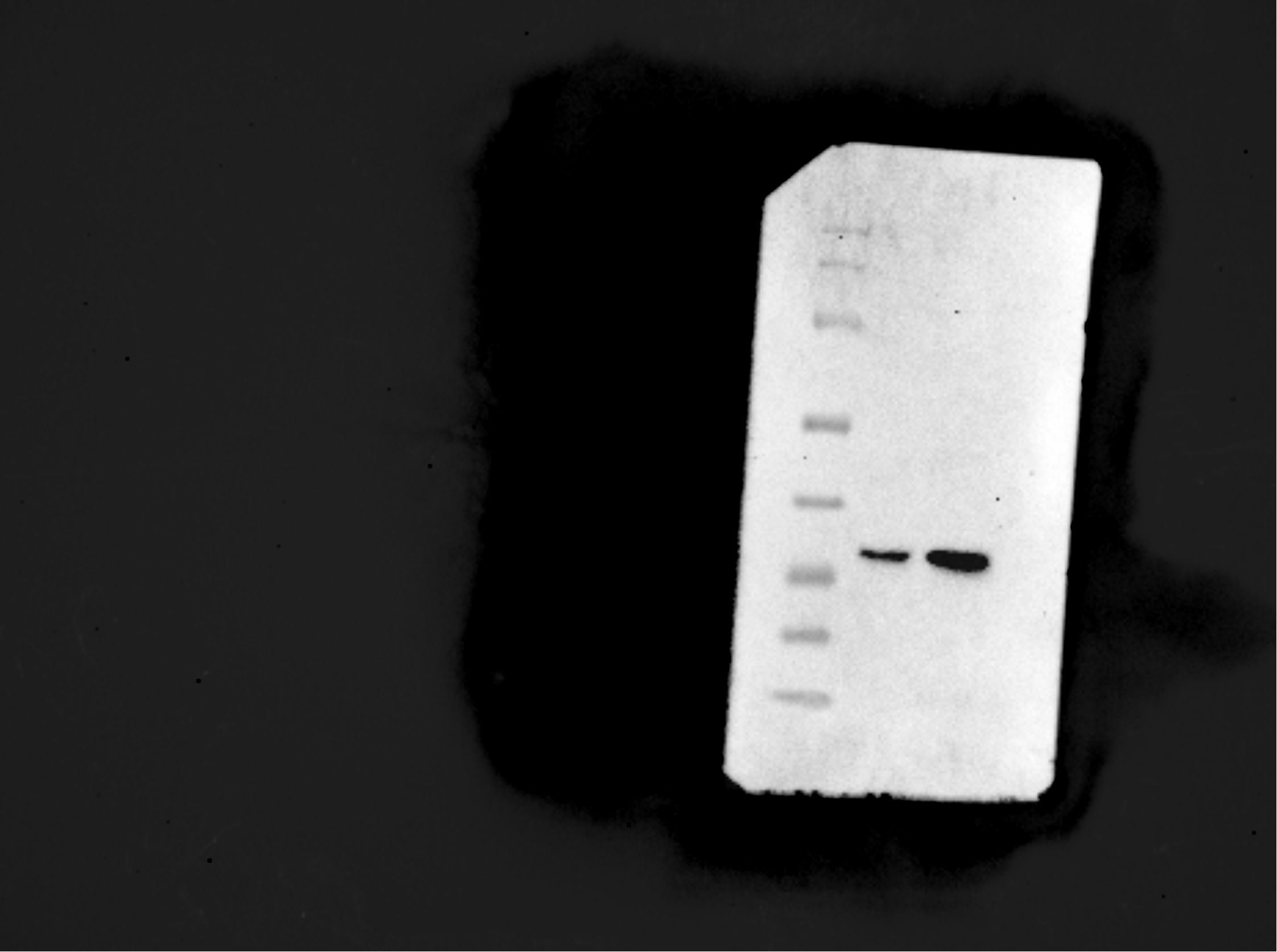


Figure 3A-1


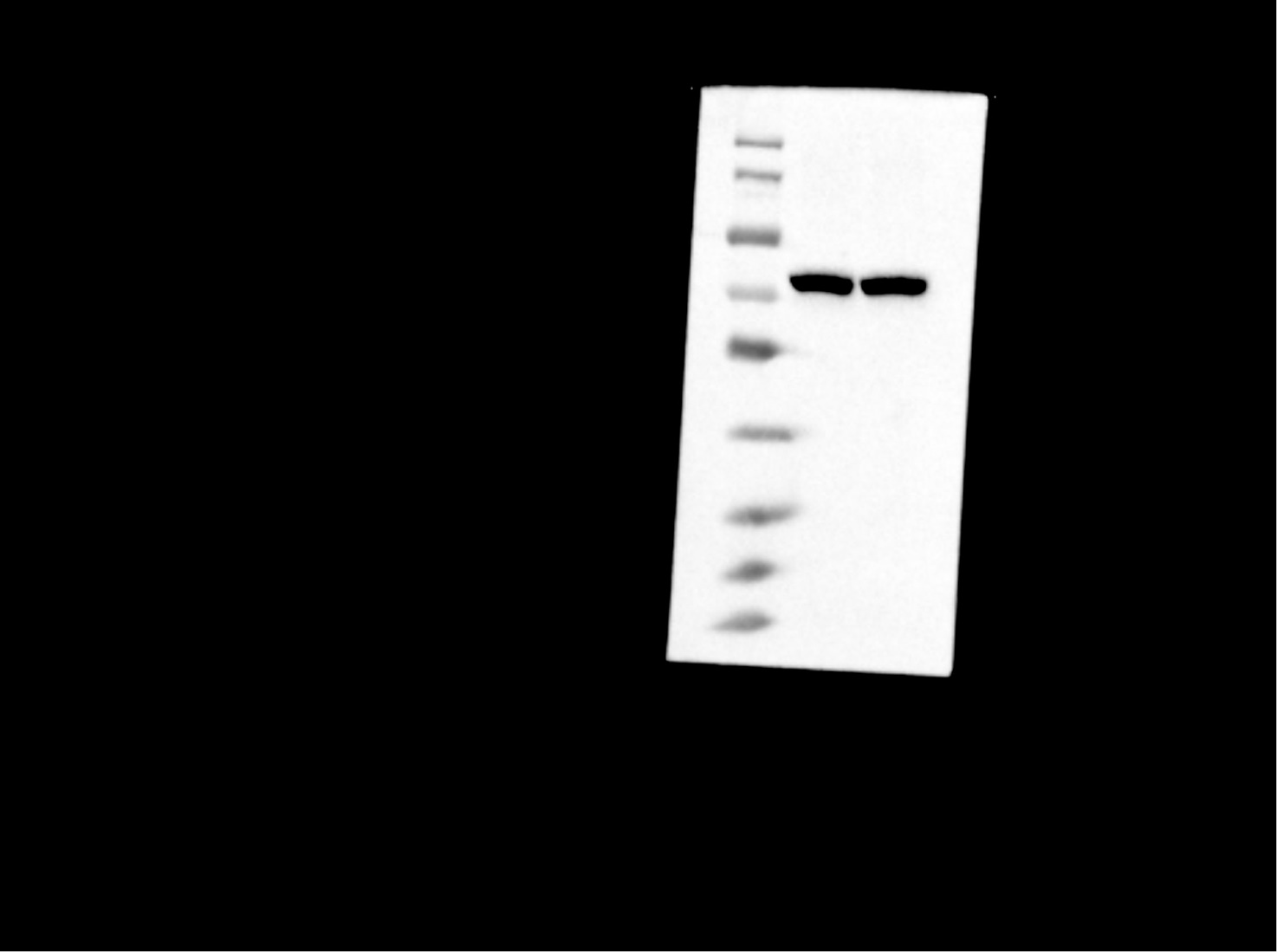


Figure 3A-2


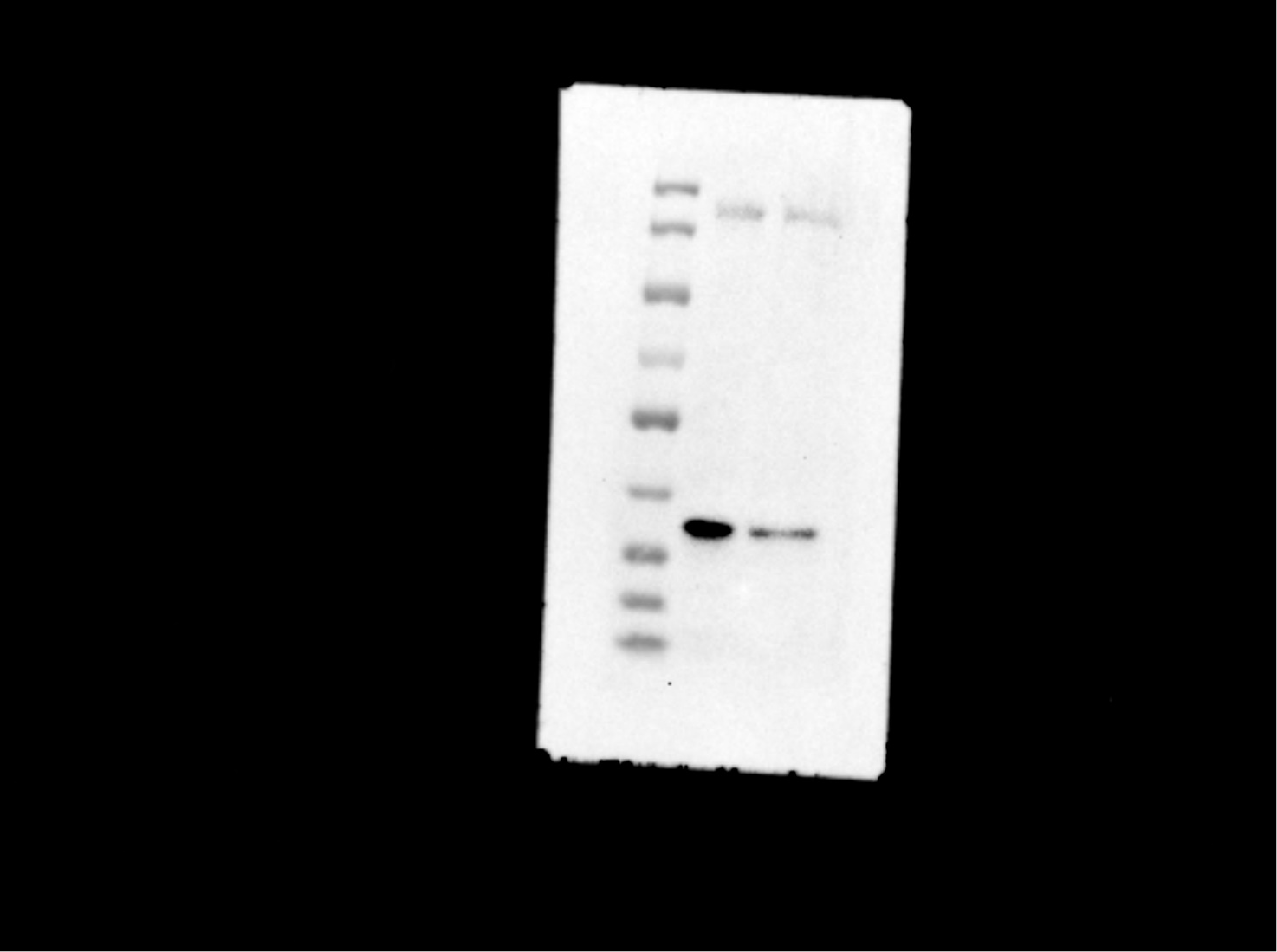


Figure 3B-1


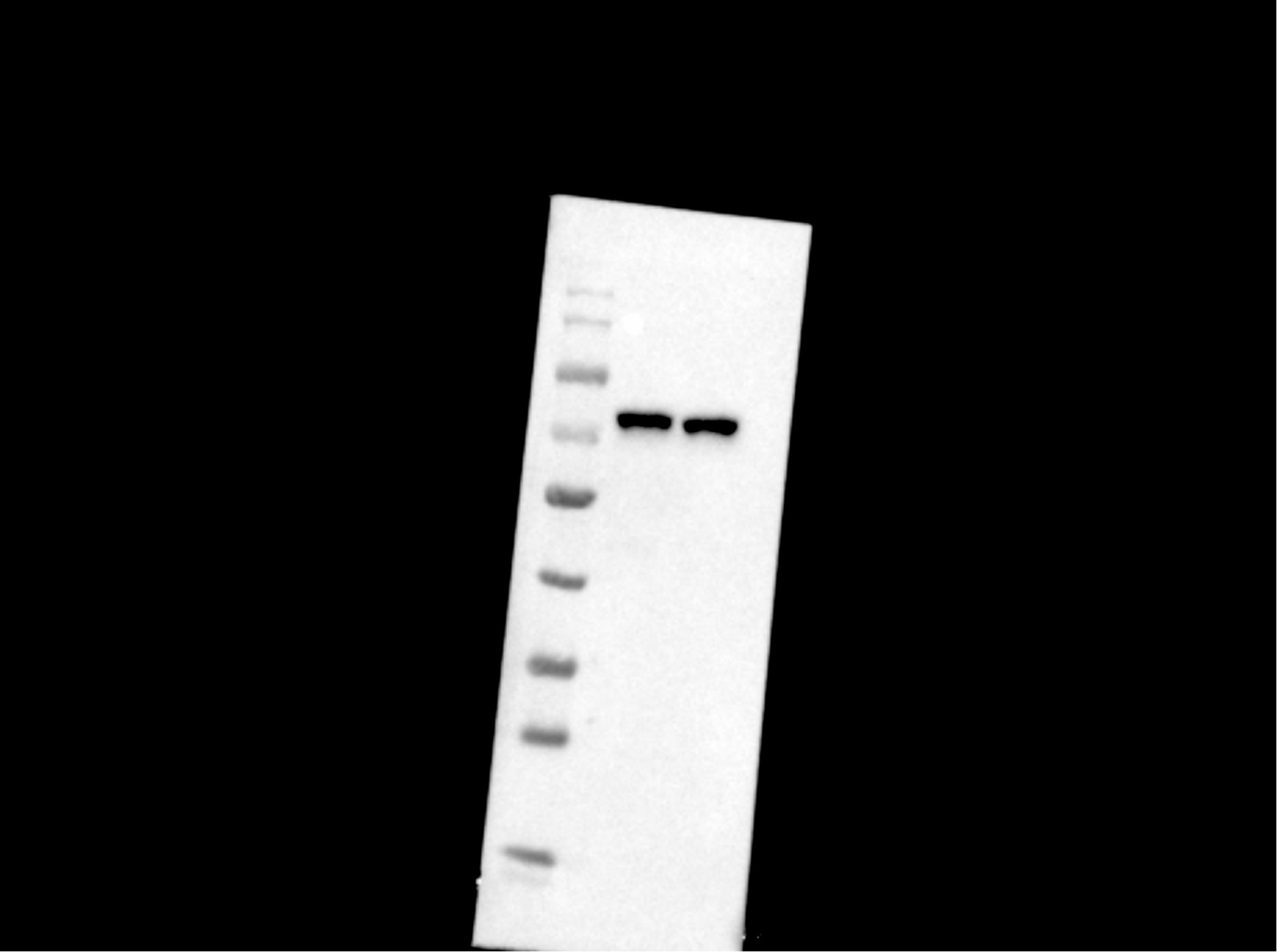


Figure 3B-2


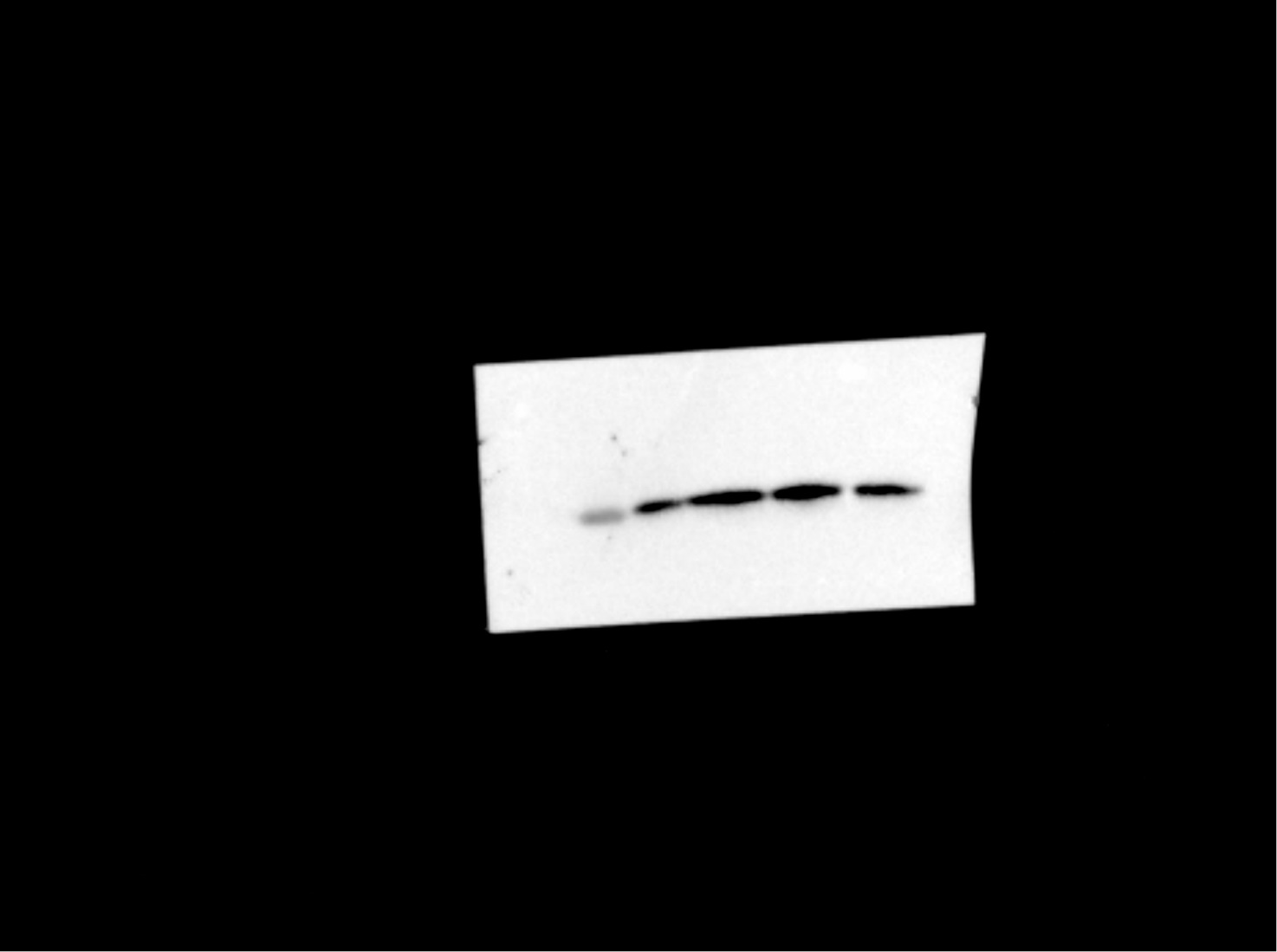


Figure 3E-1


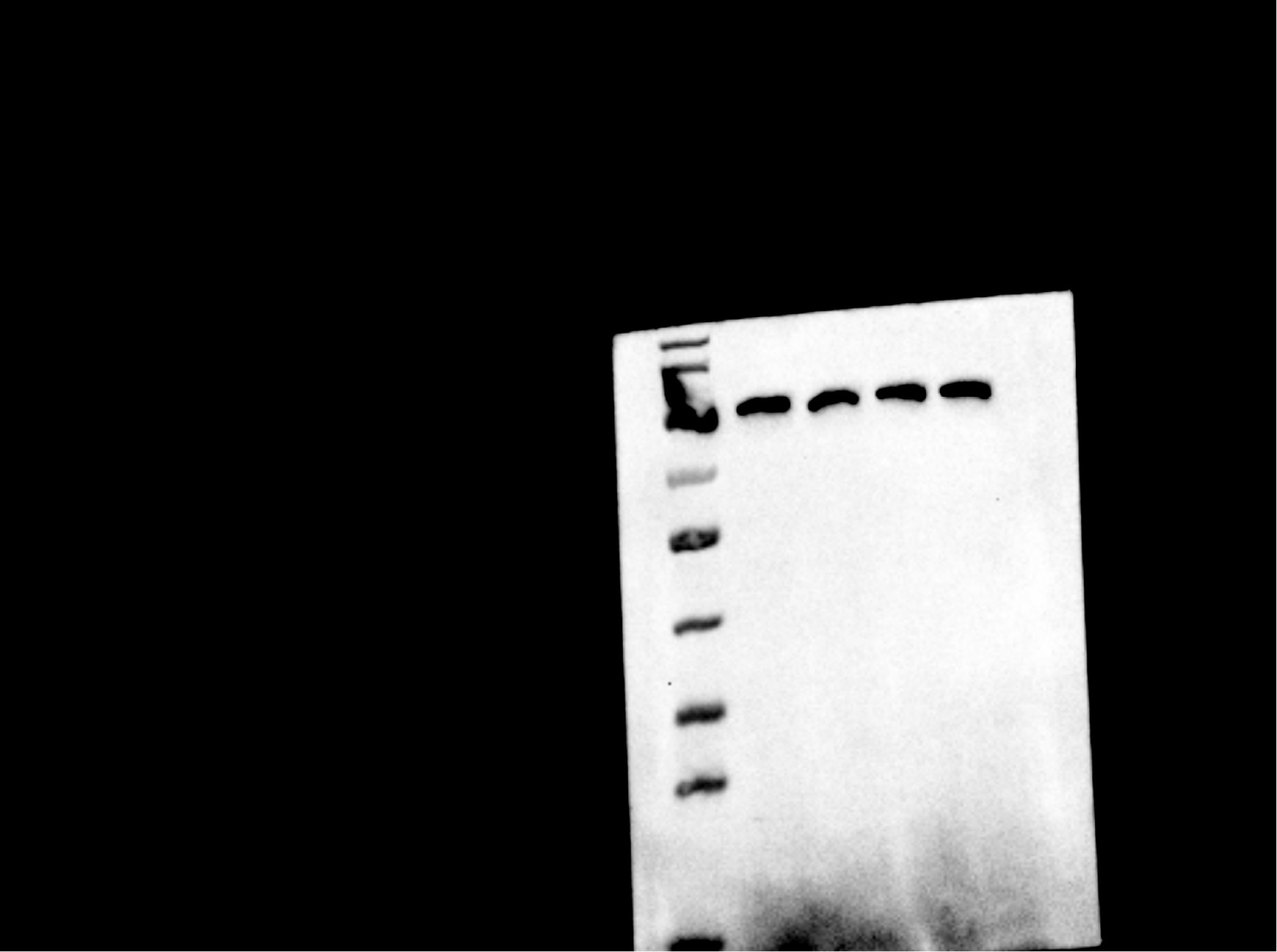


Figure 3E-2


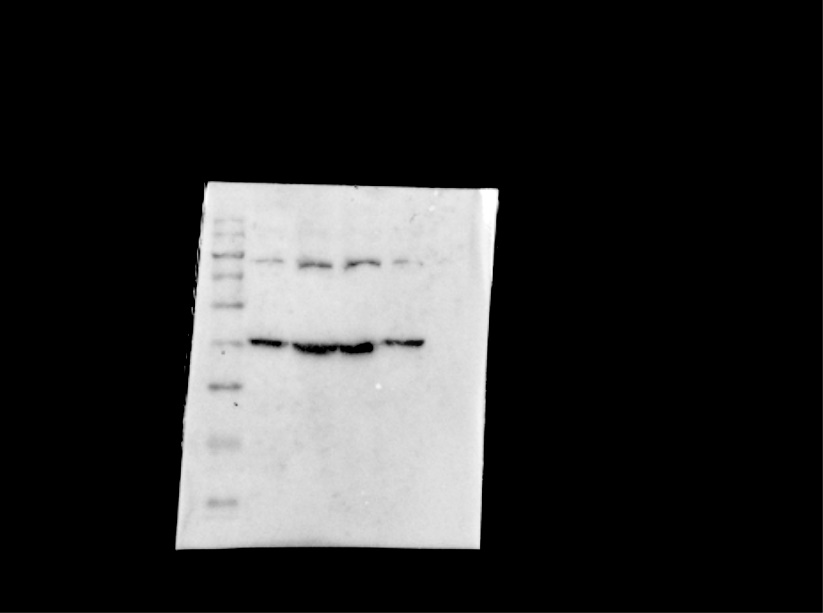


Figure 3K-1


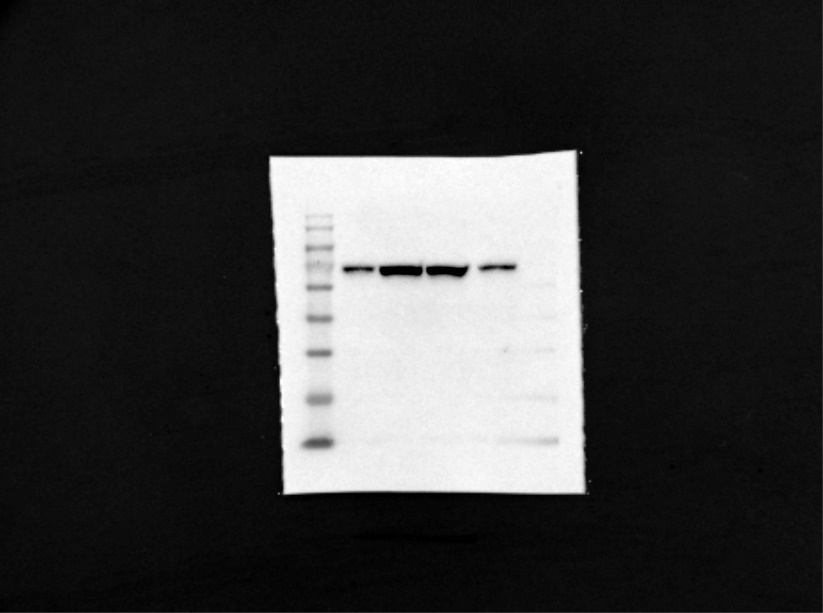


Figure 3K-2


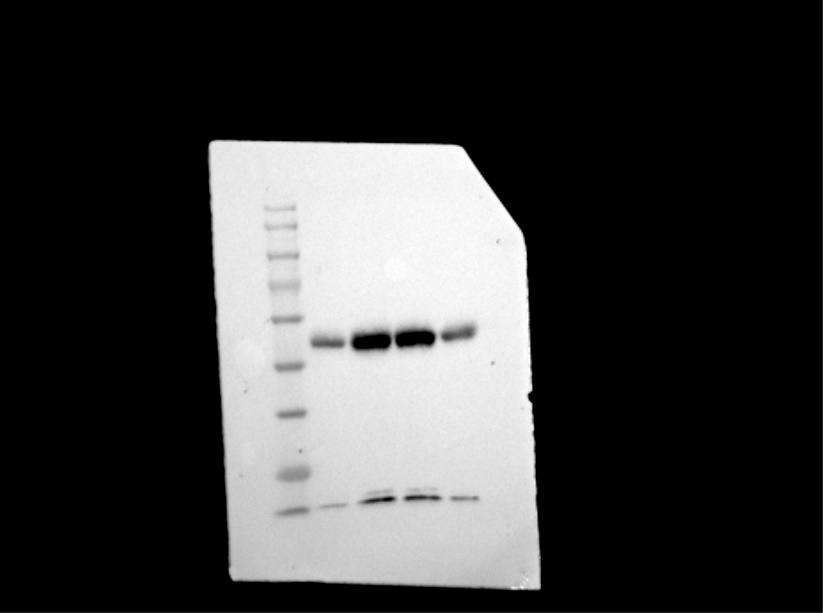


Figure 3K-3


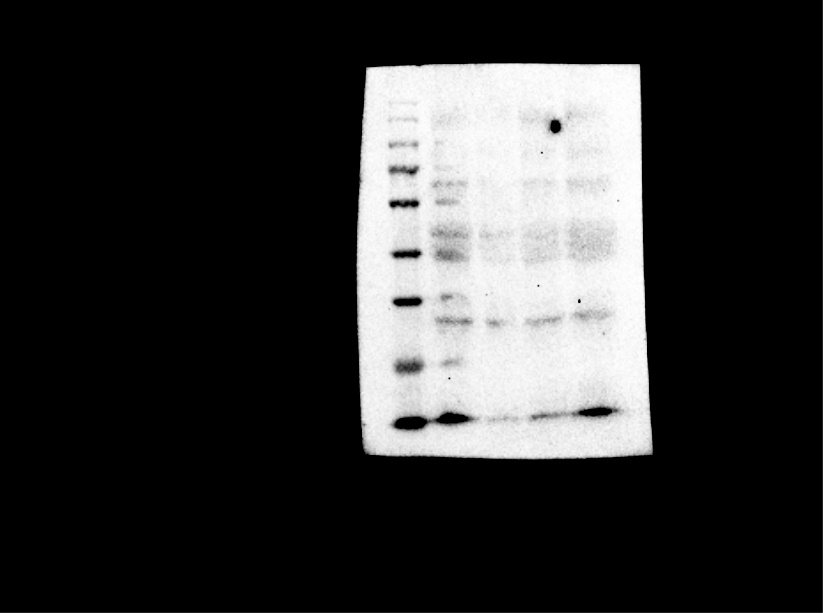


Figure 3K-4


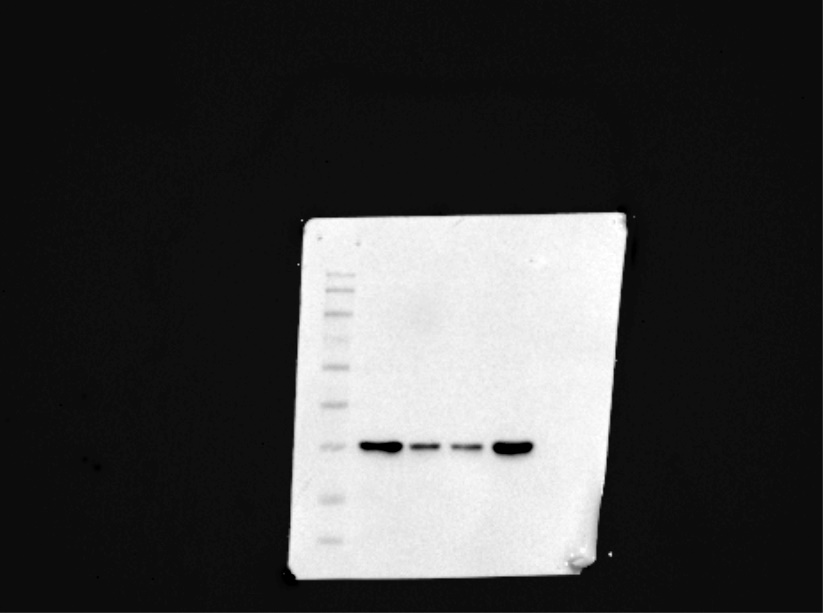


Figure 3K-5


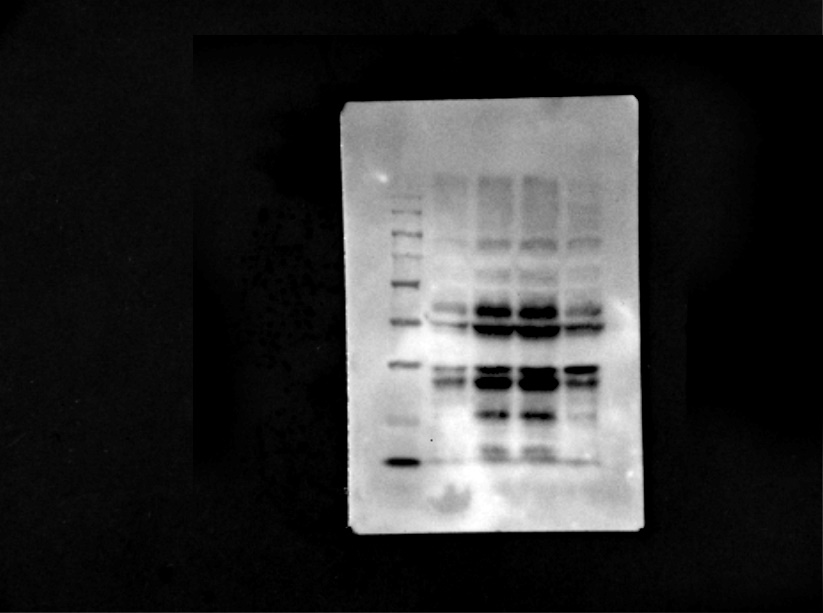


Figure 3K-6


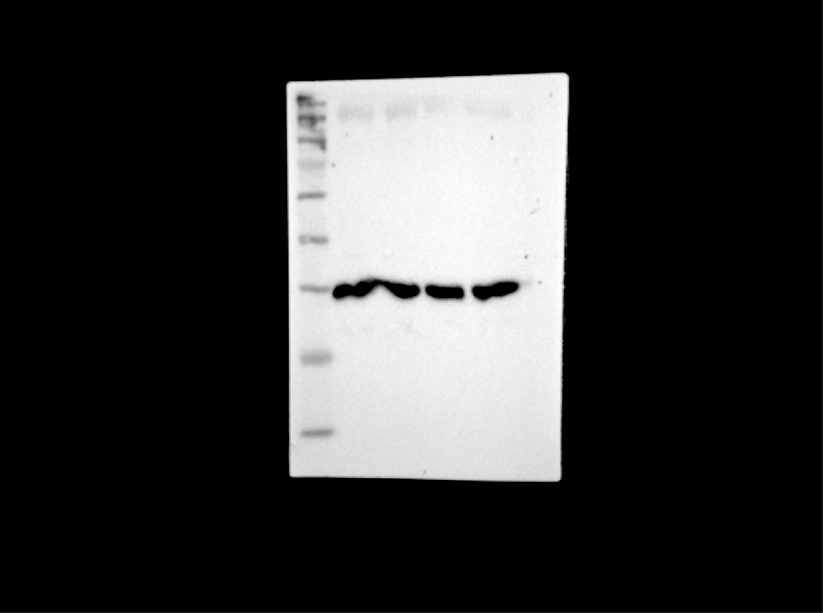


Figure 3K-7


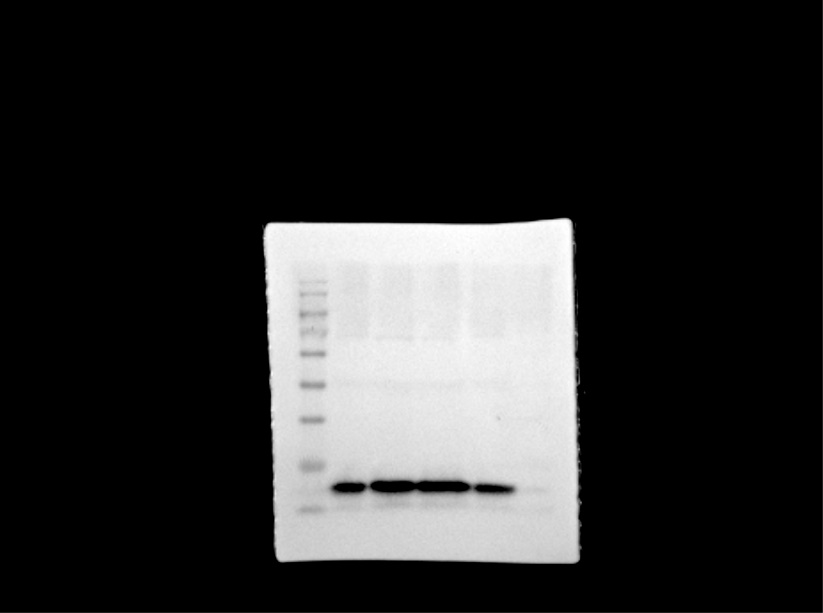


Figure 3K-8


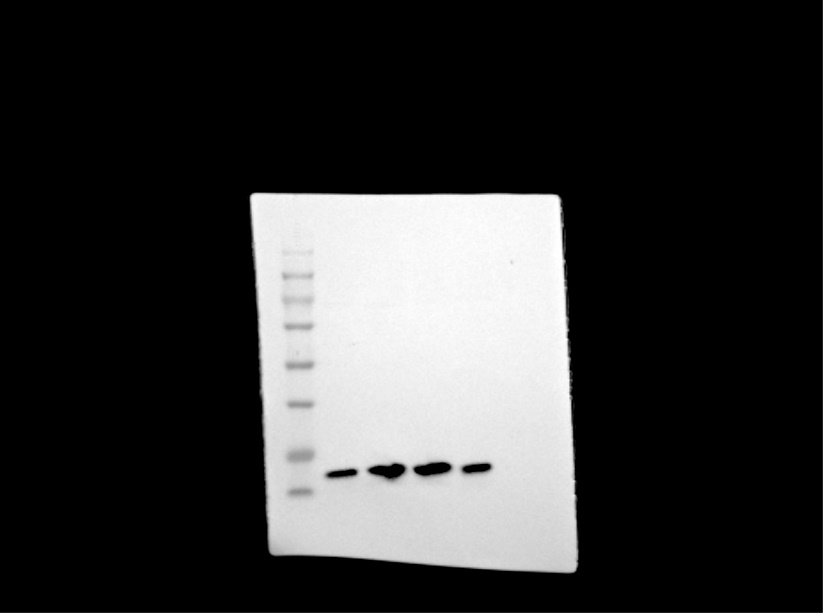


Figure 3K-9


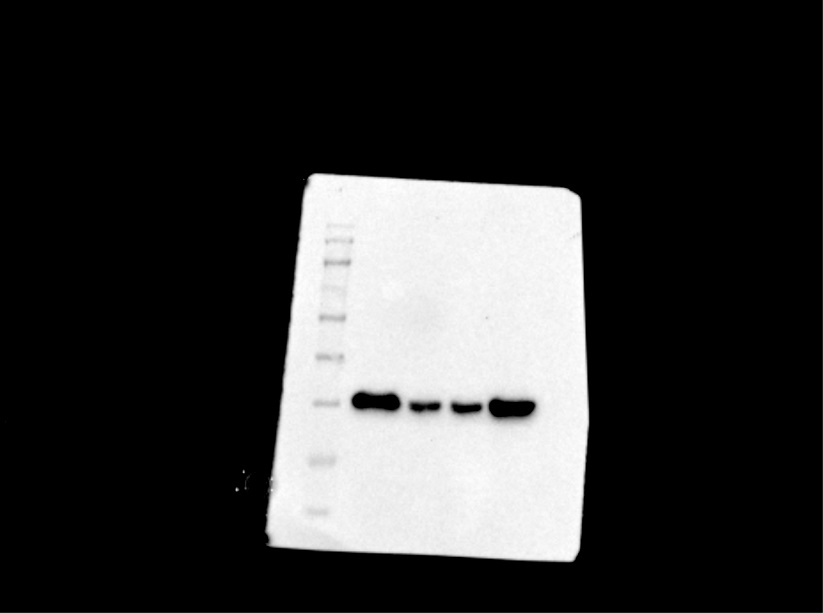


Figure 3K-10


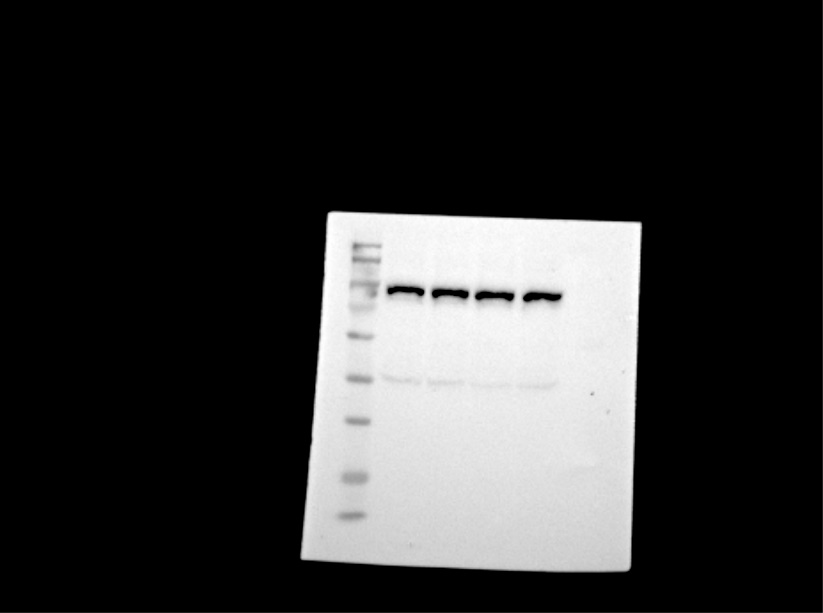


Figure 3K-11


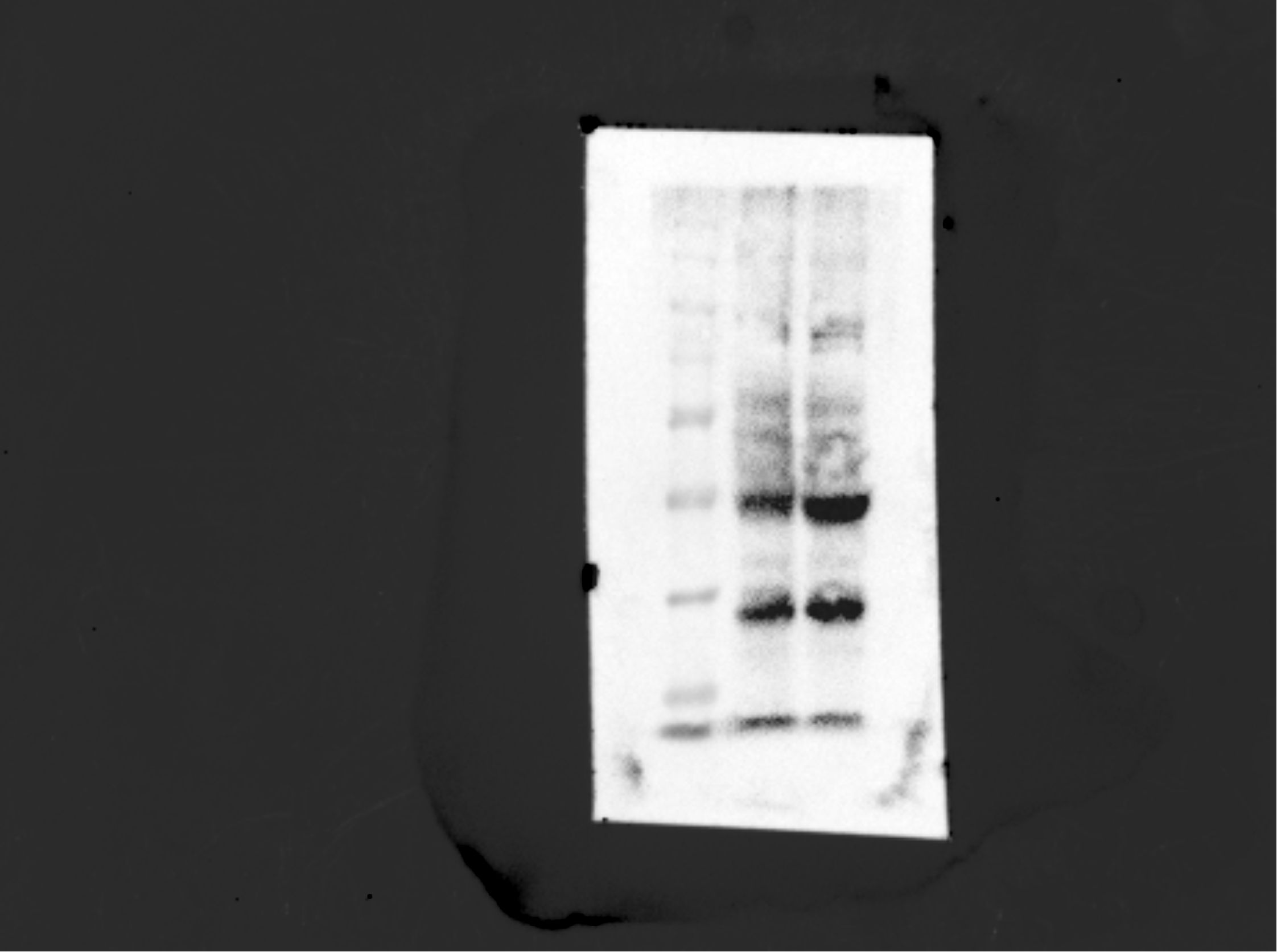


Figure 4E-1X


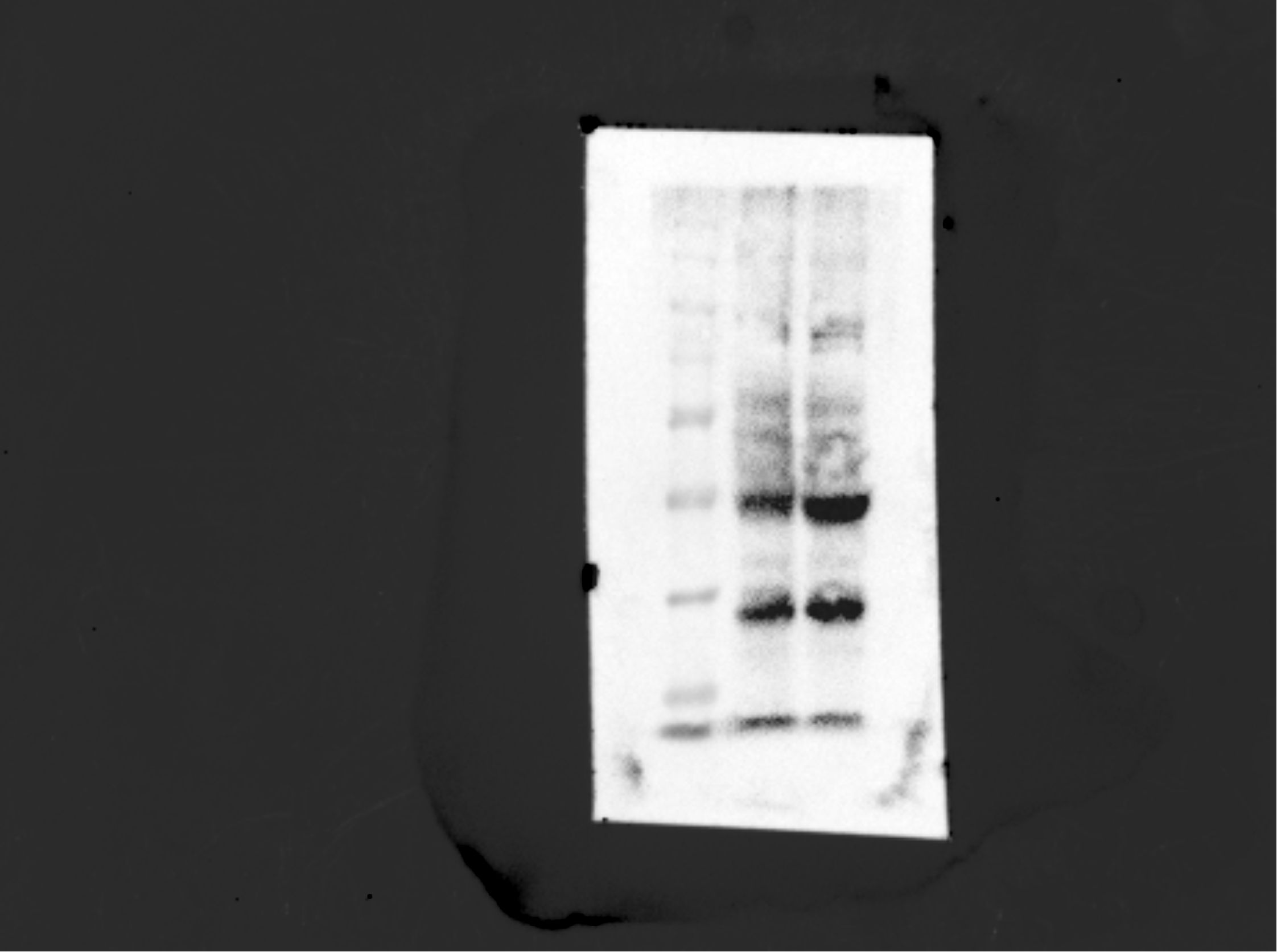


Figure 4E-2S


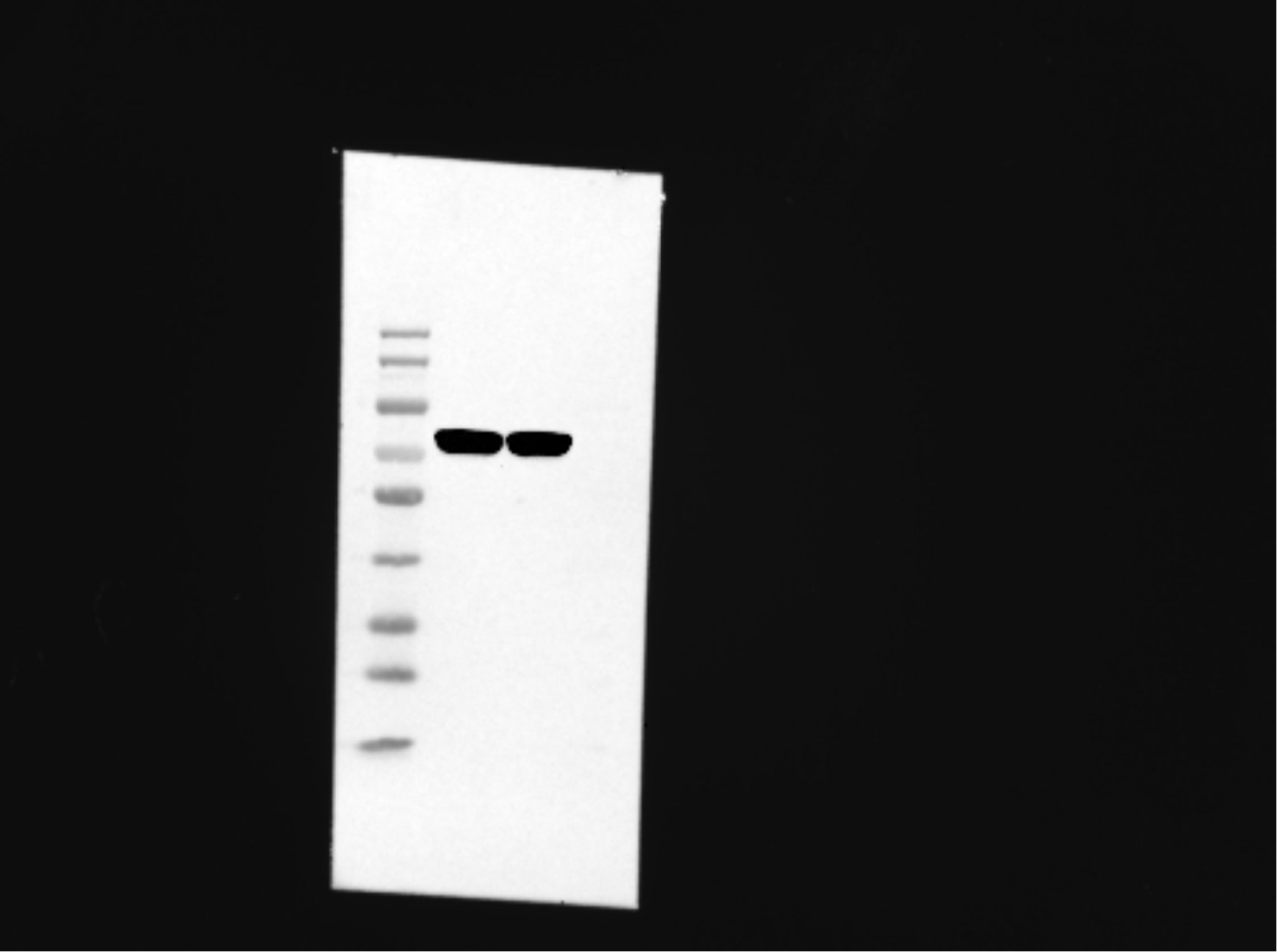


Figure 4E-3


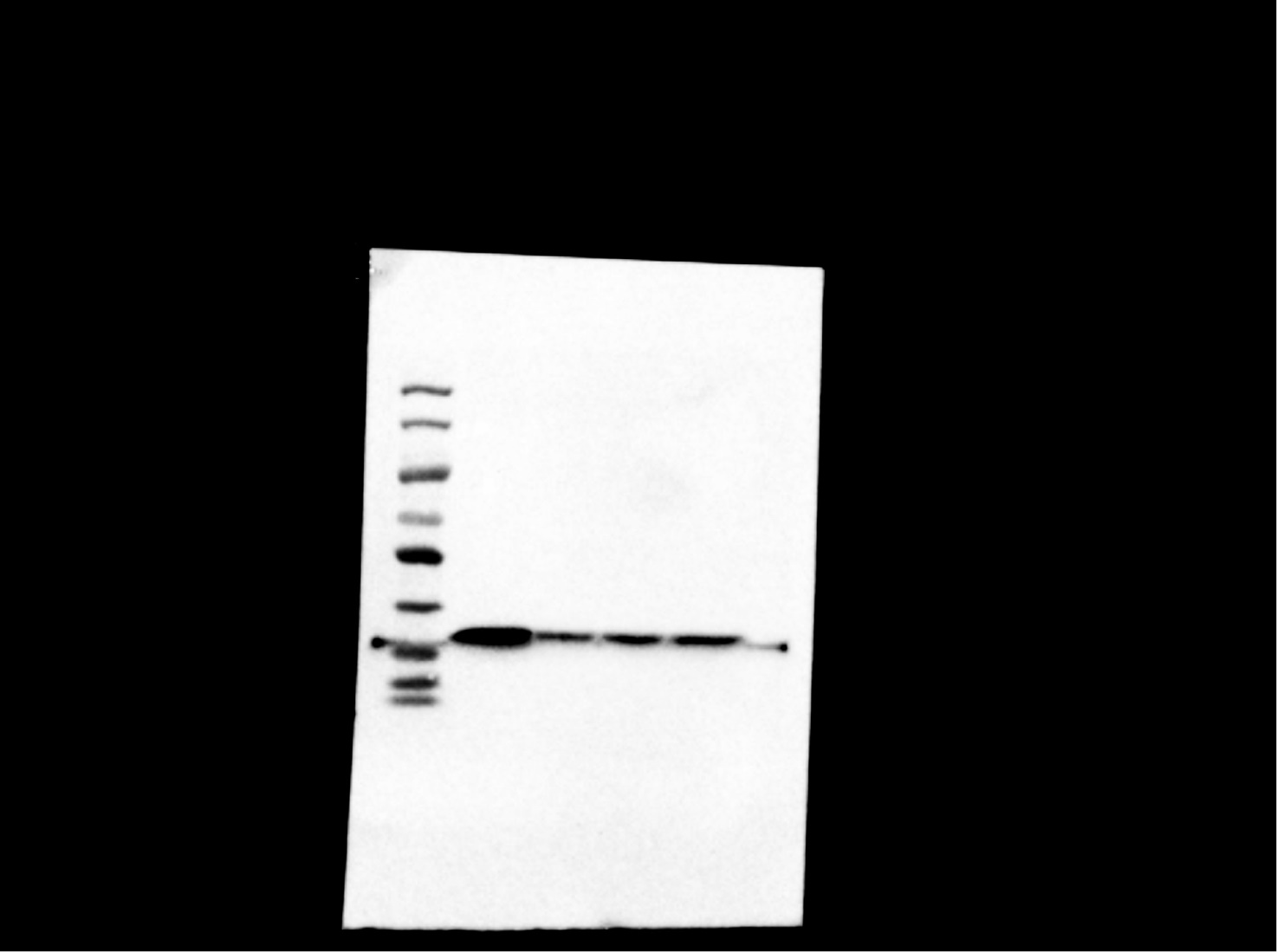


Figure 4F-1


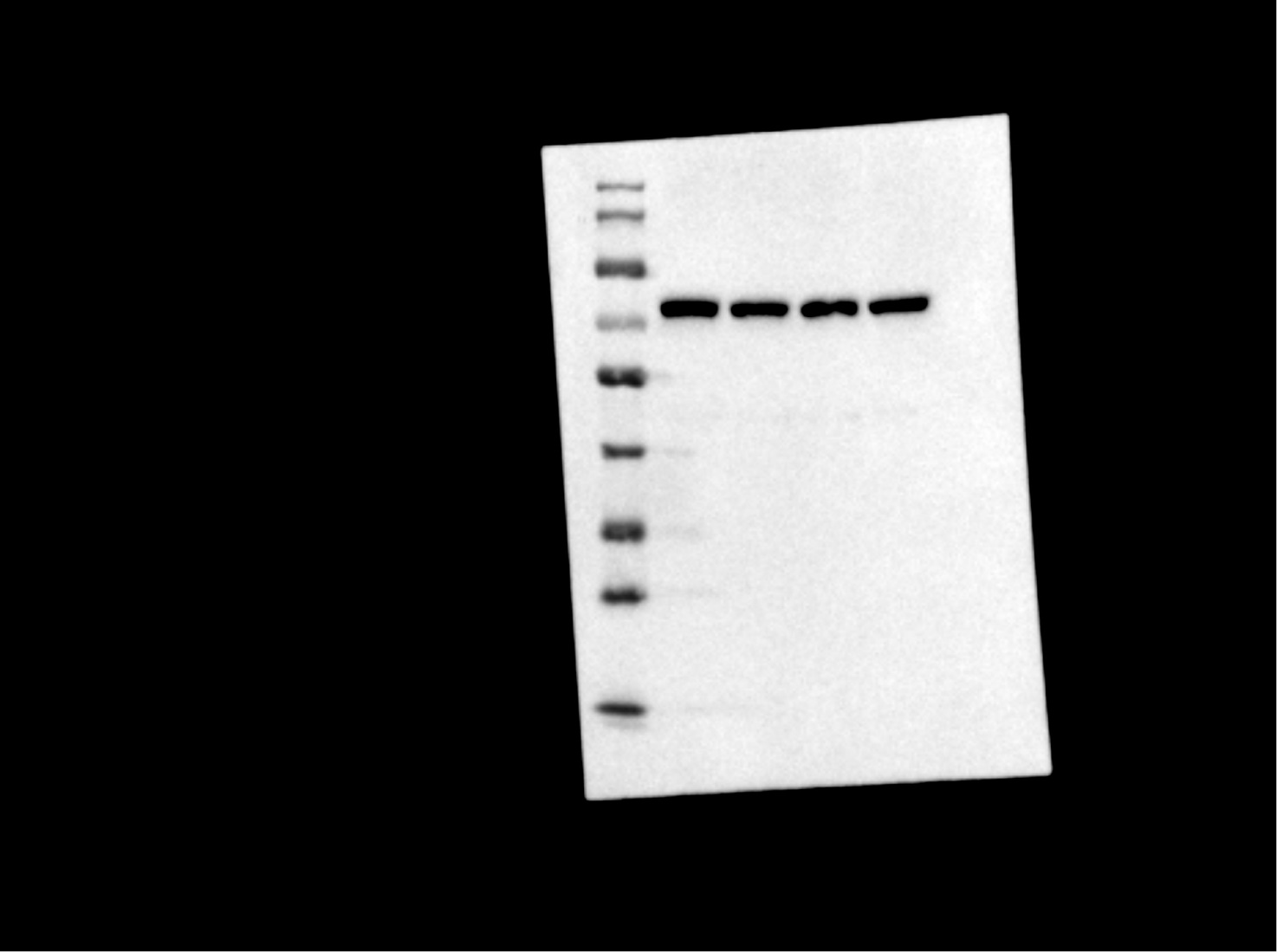


Figure 4F-2


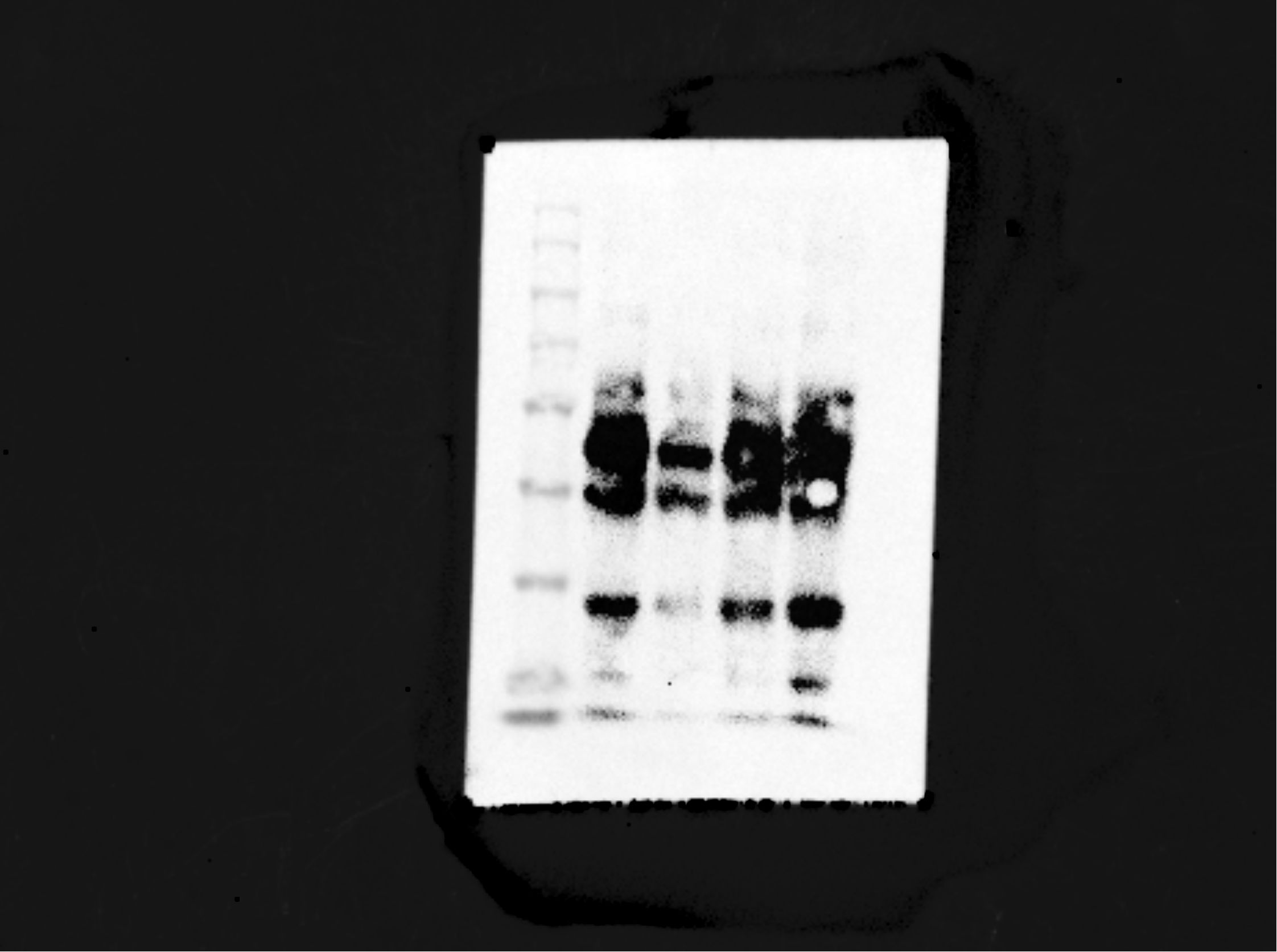


Figure 4G-1


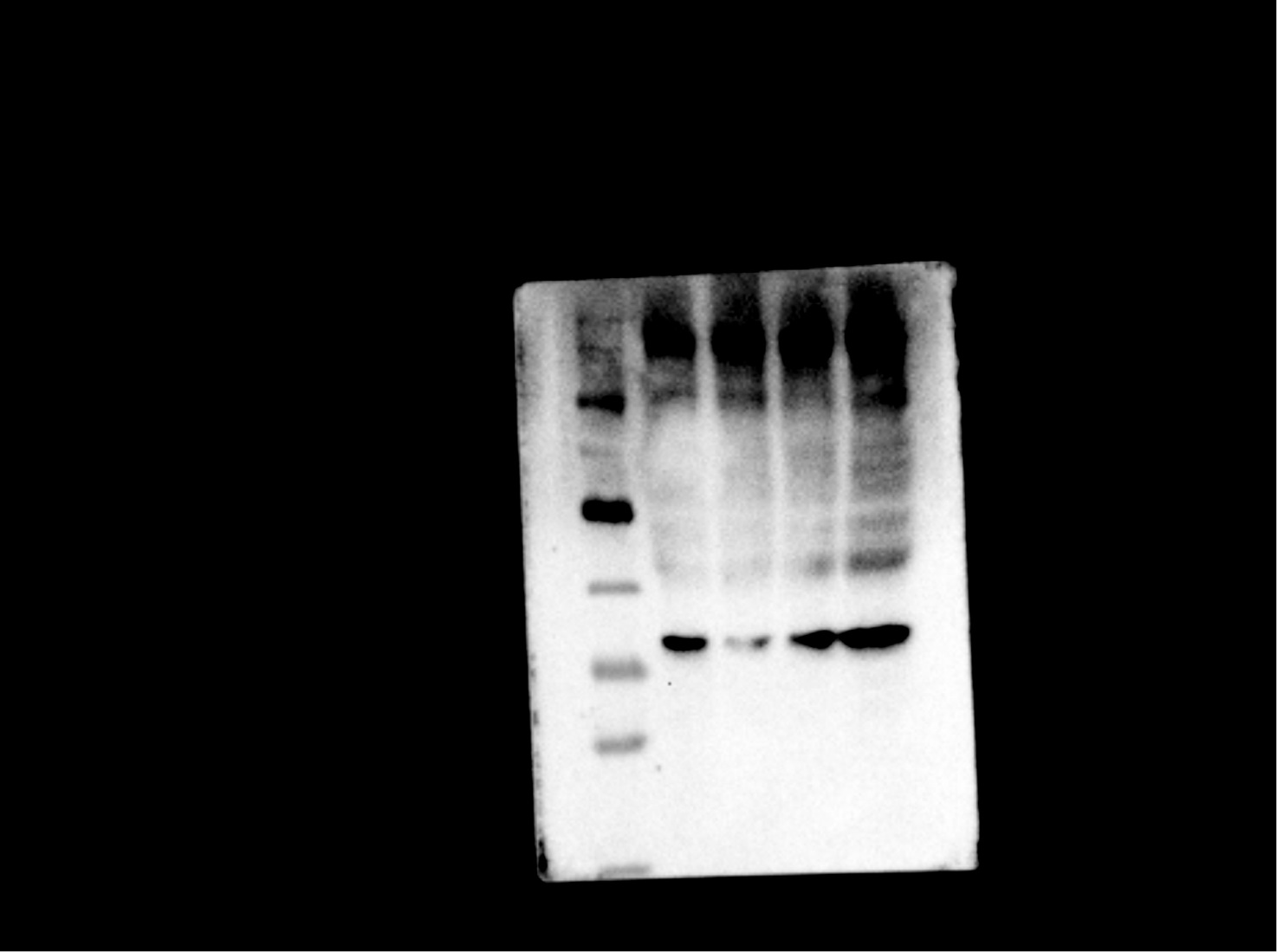


Figure 4G-2


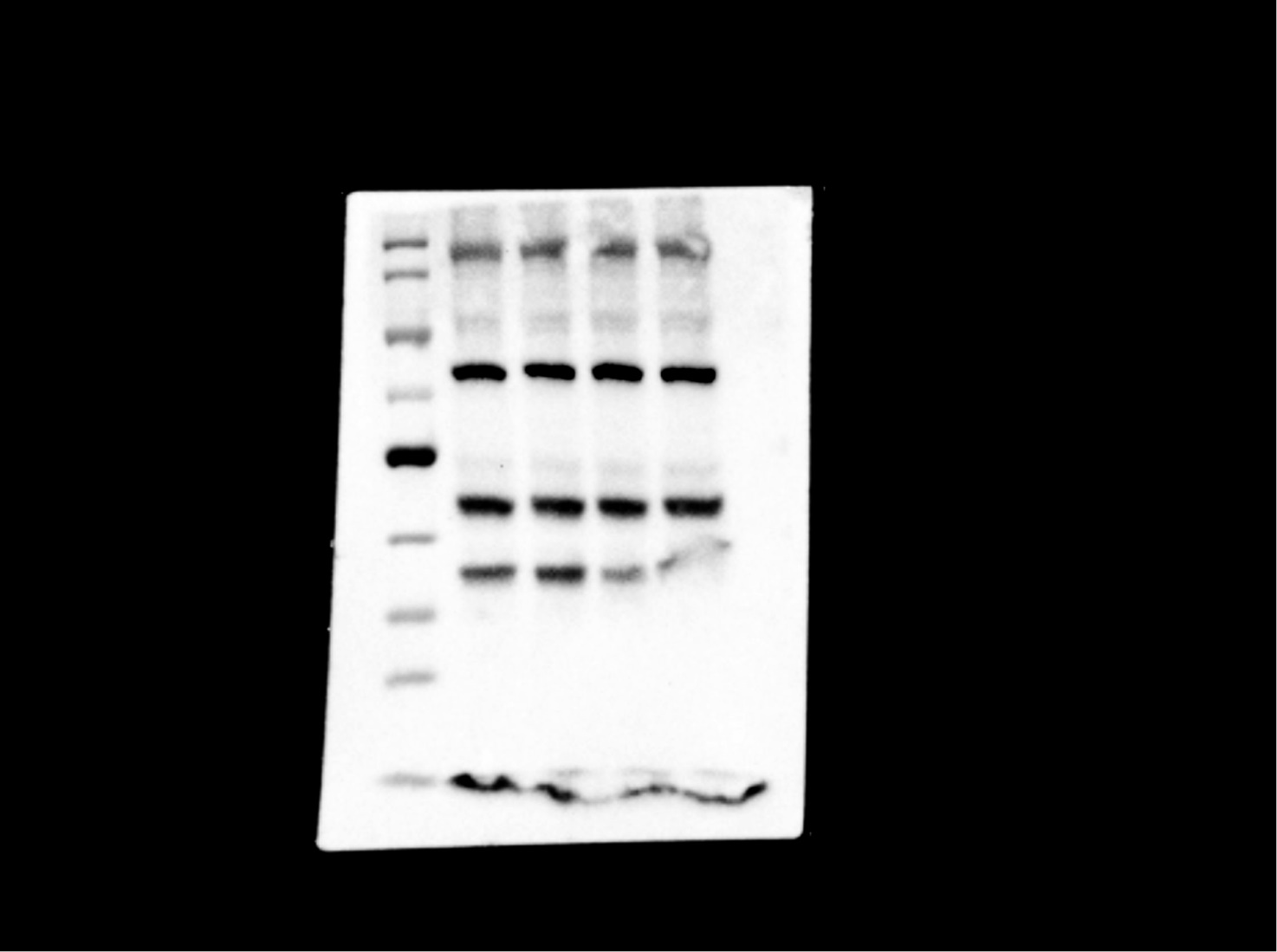


Figure 4G-3


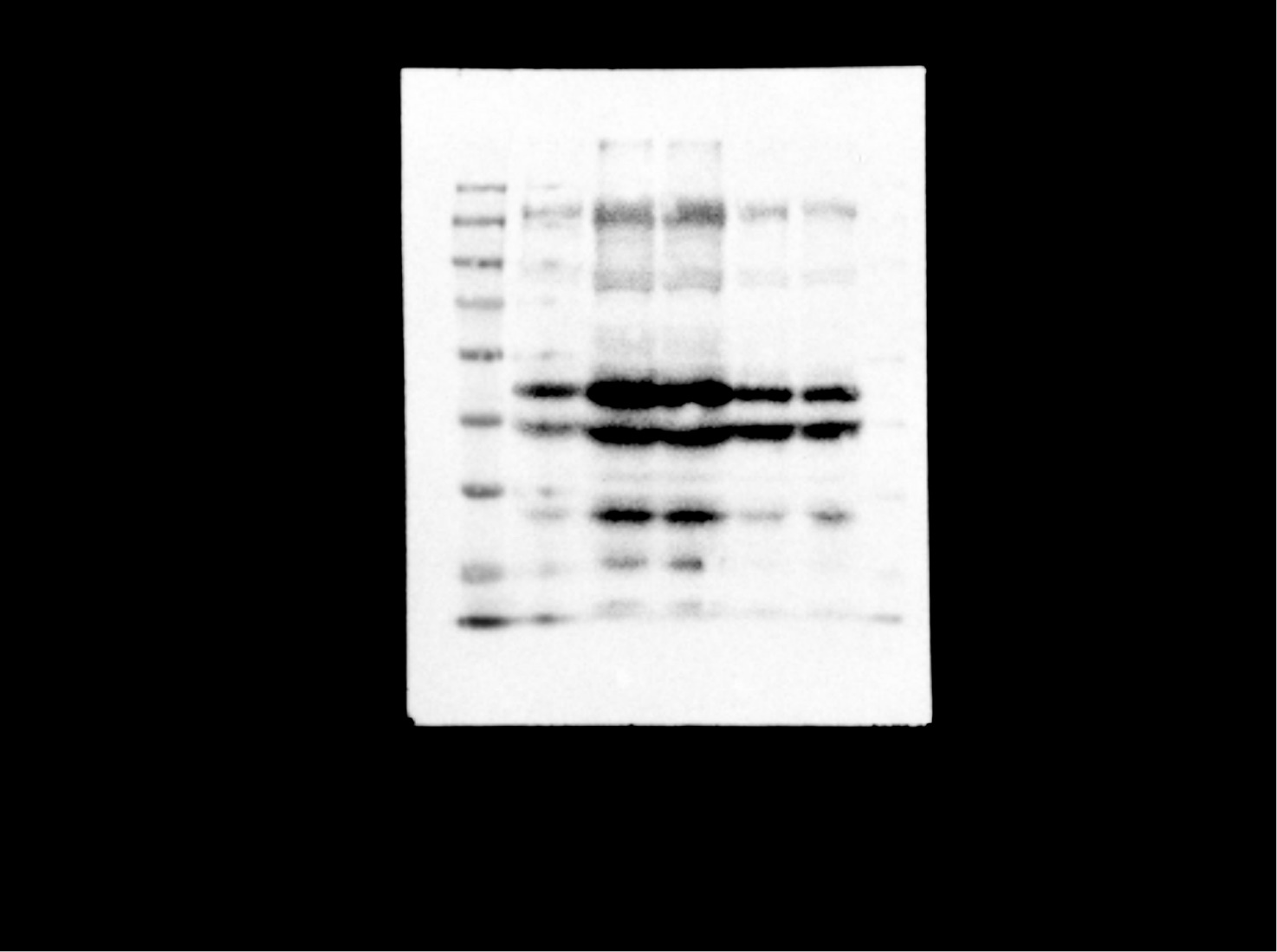


Figure 5B-1X


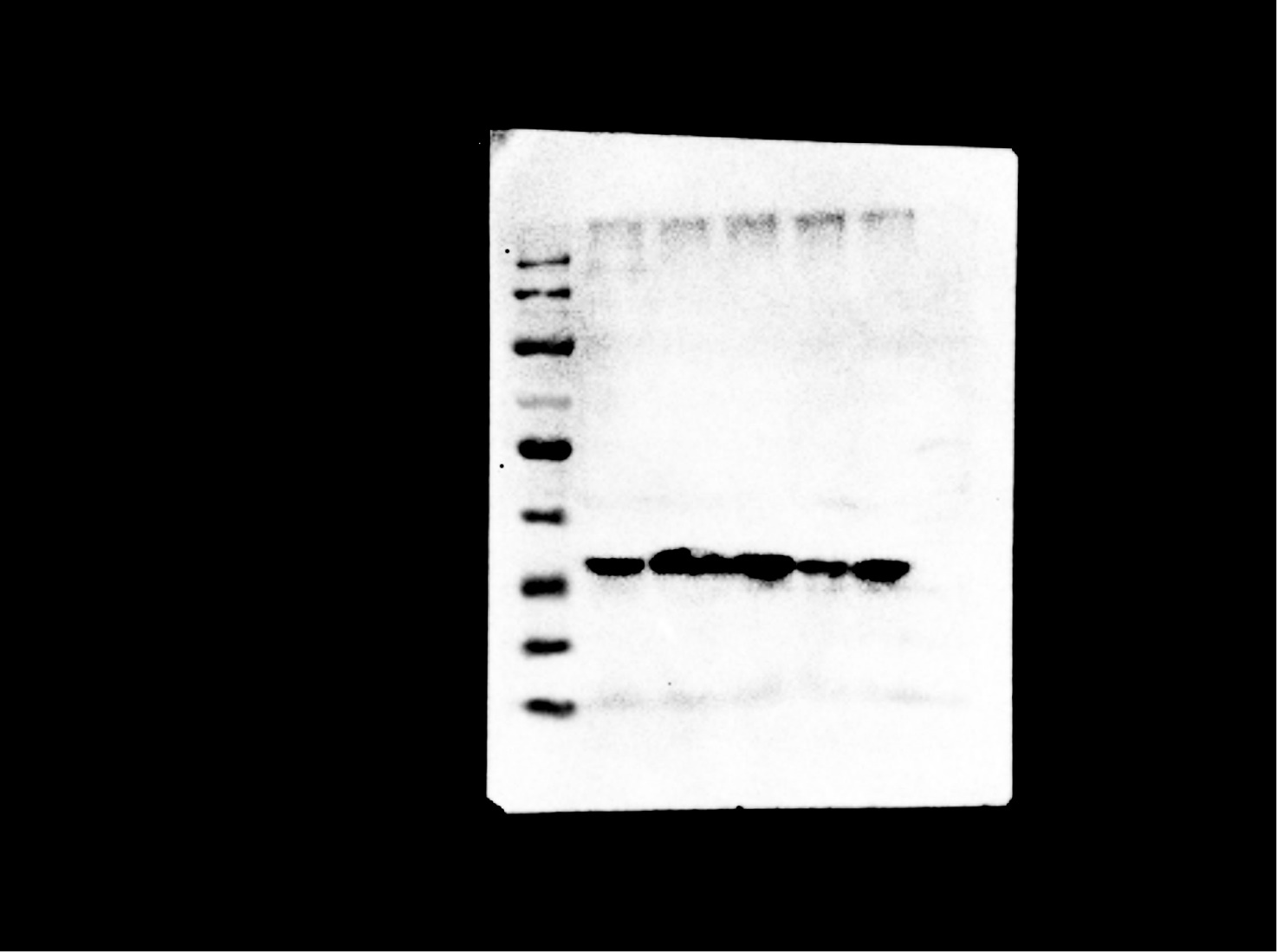


Figure 5B-2


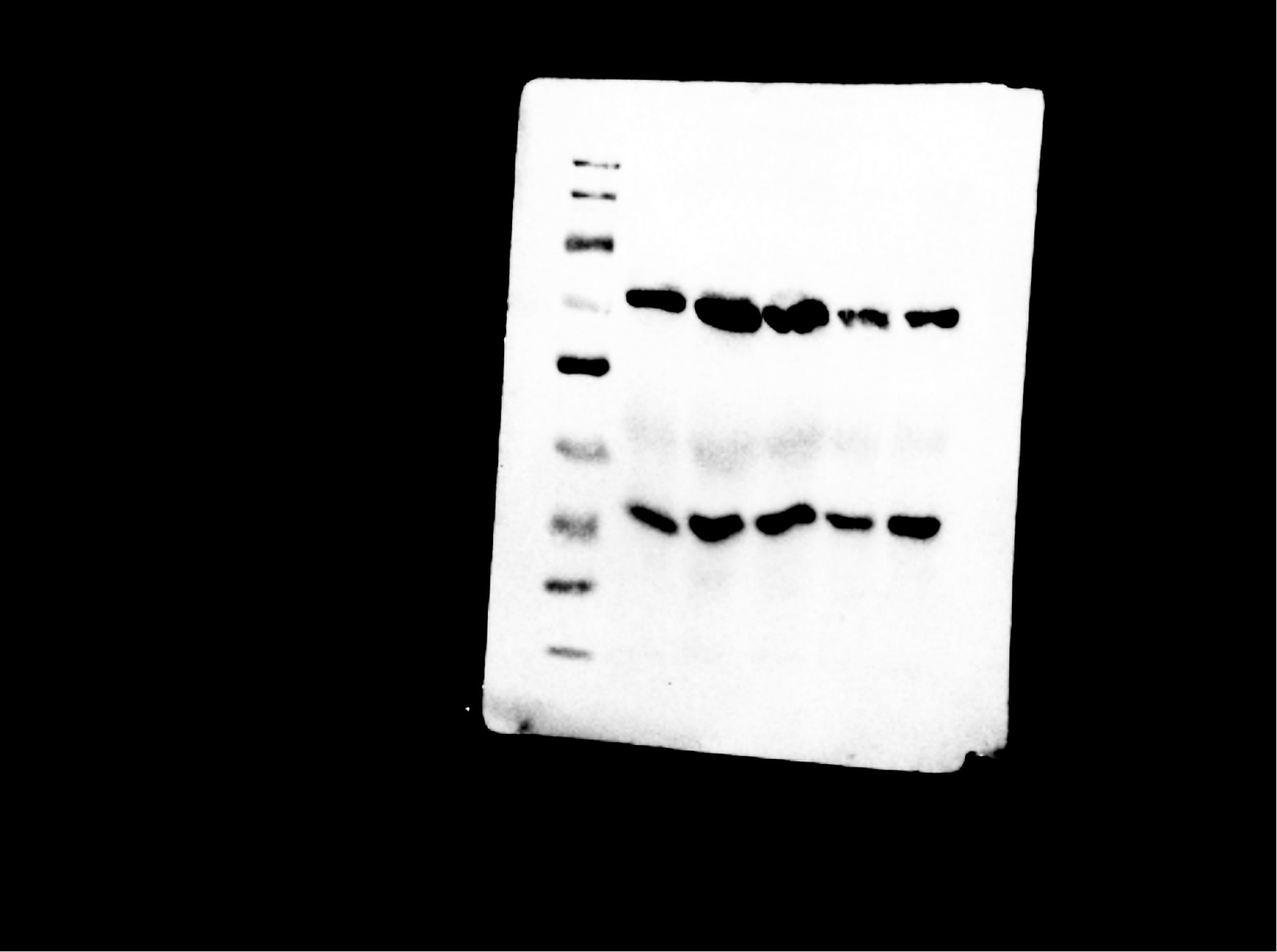


Figure 5B-3X


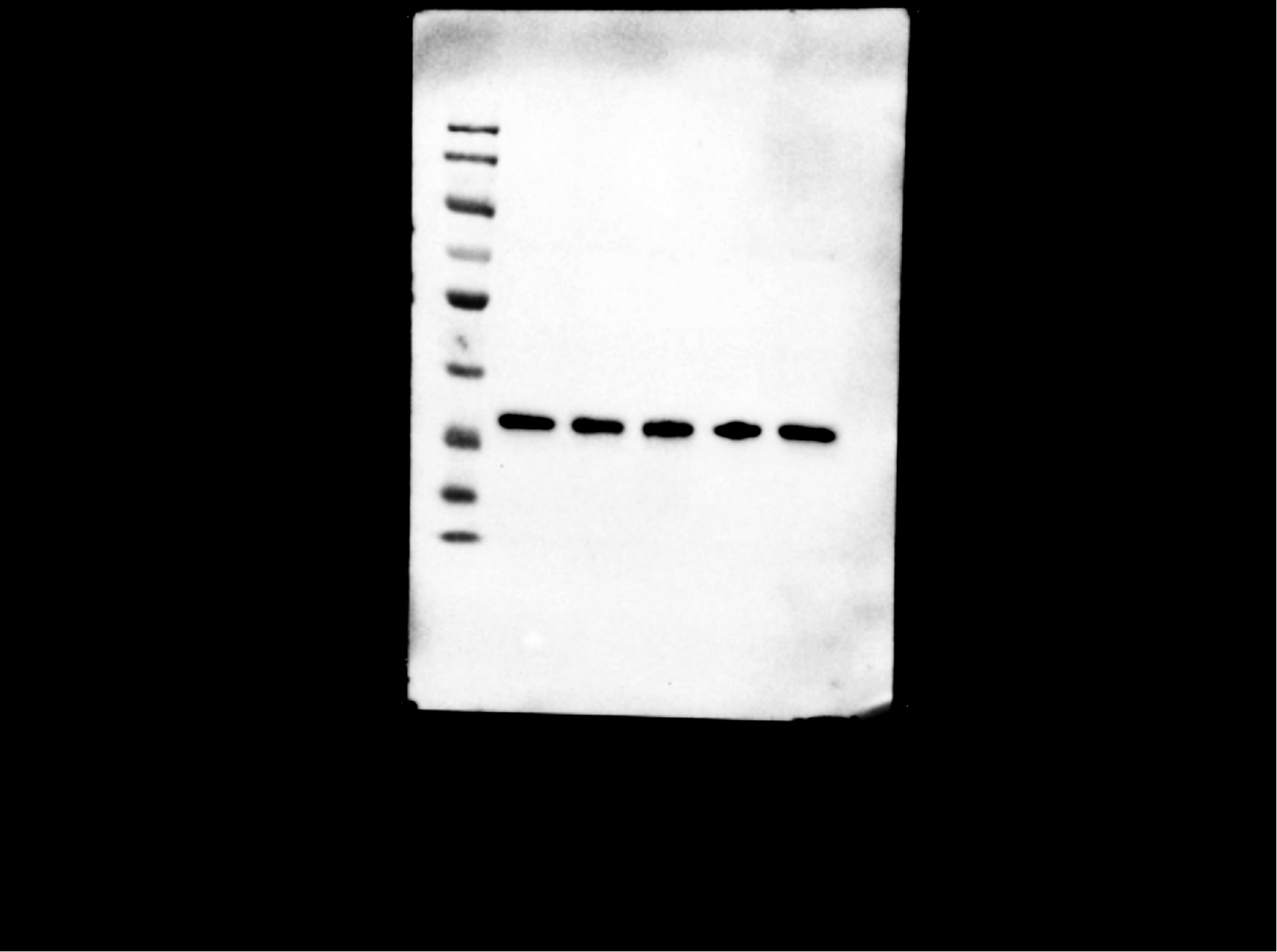


Figure 5B-4


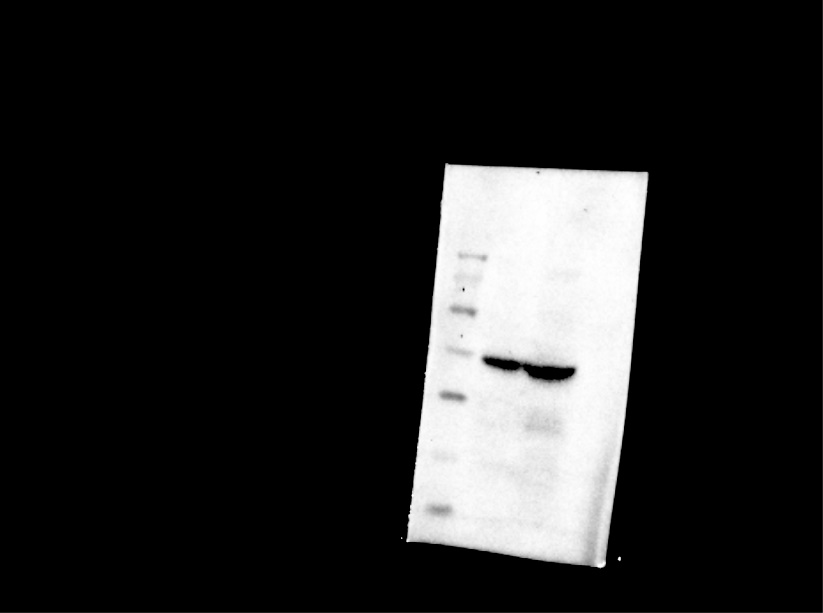


Figure 5I-1-1


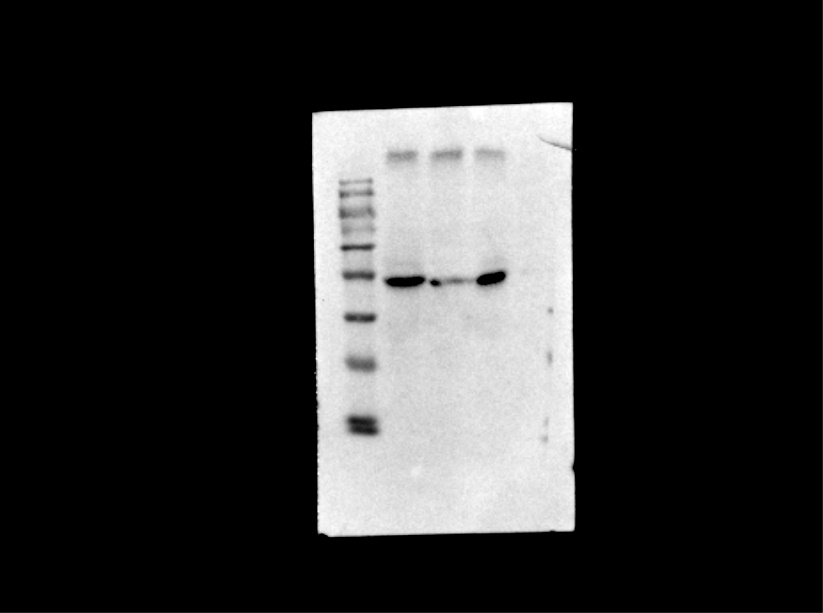


Figure 5I-1-2


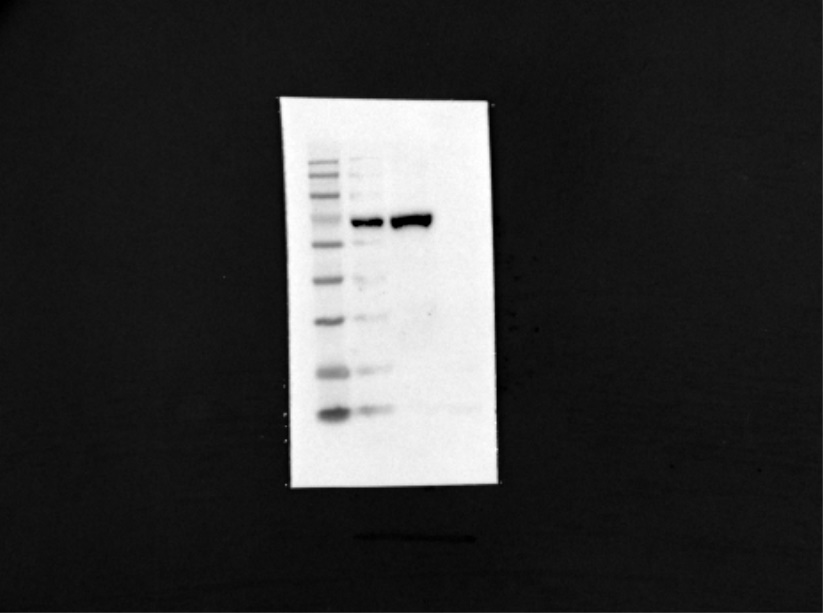


Figure 5I-2-1


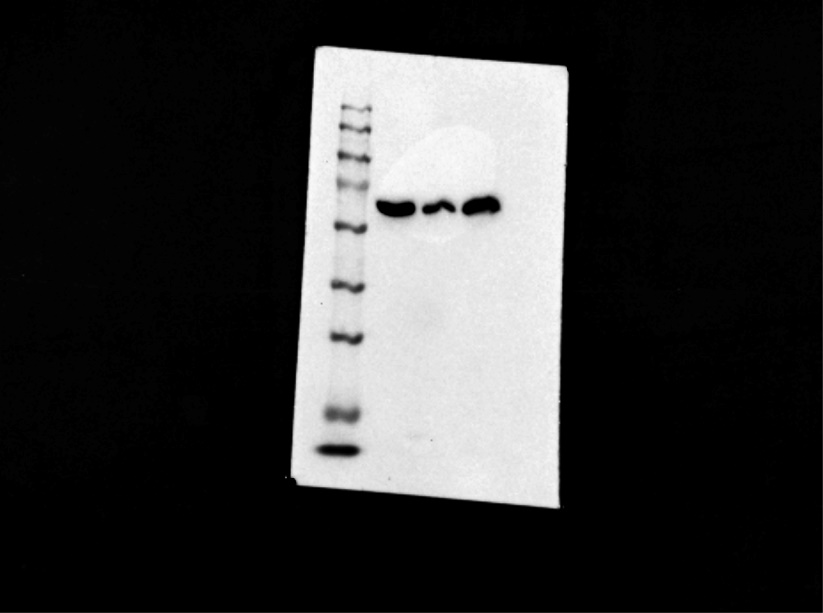


Figure 5I-2-2


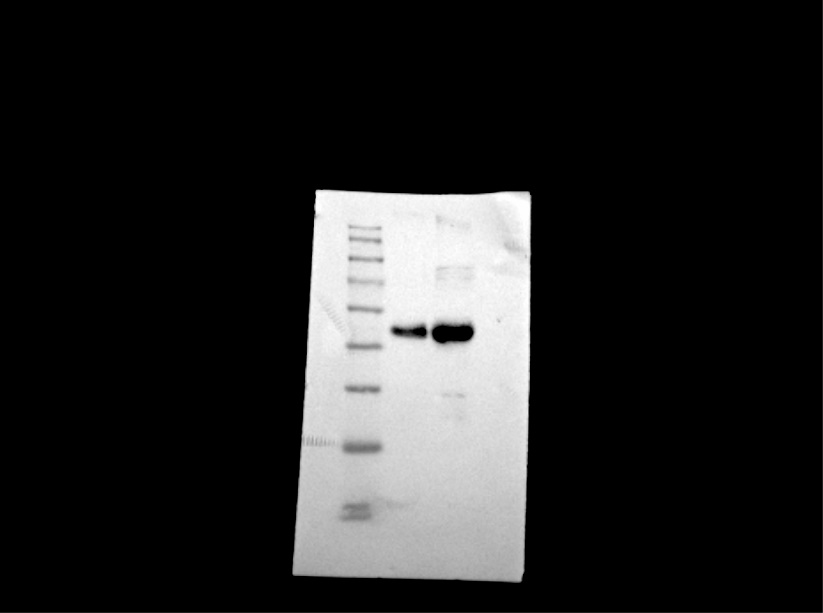


Figure 5I-3-1


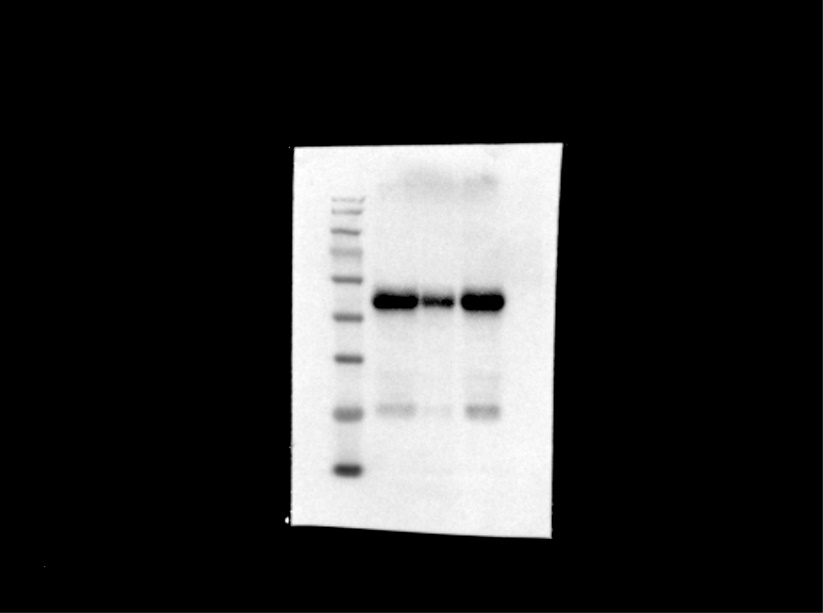


Figure 5I-3-2


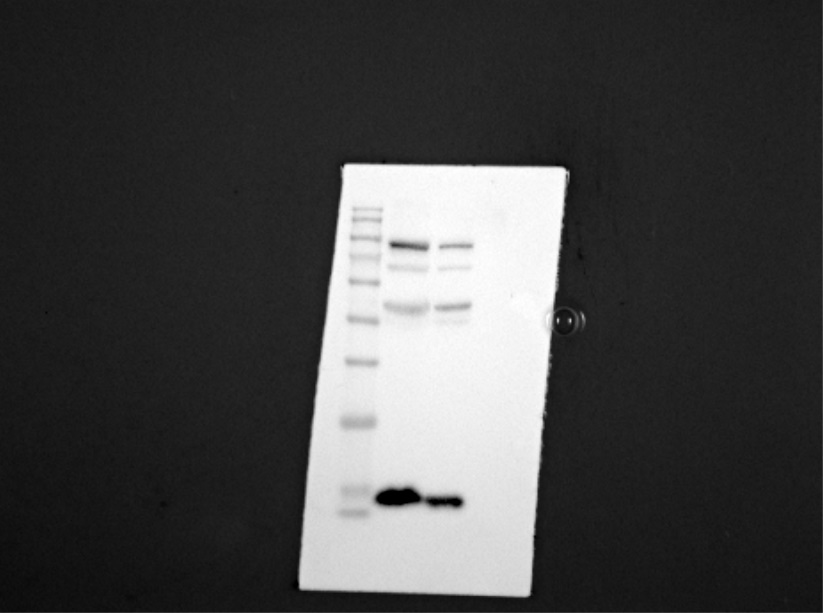


Figure 5I-4-1


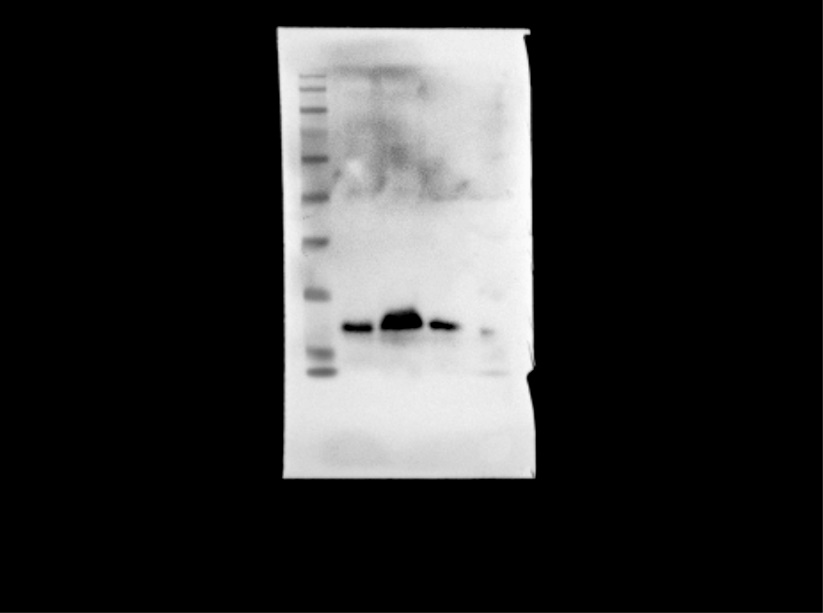


Figure 5I-4-2


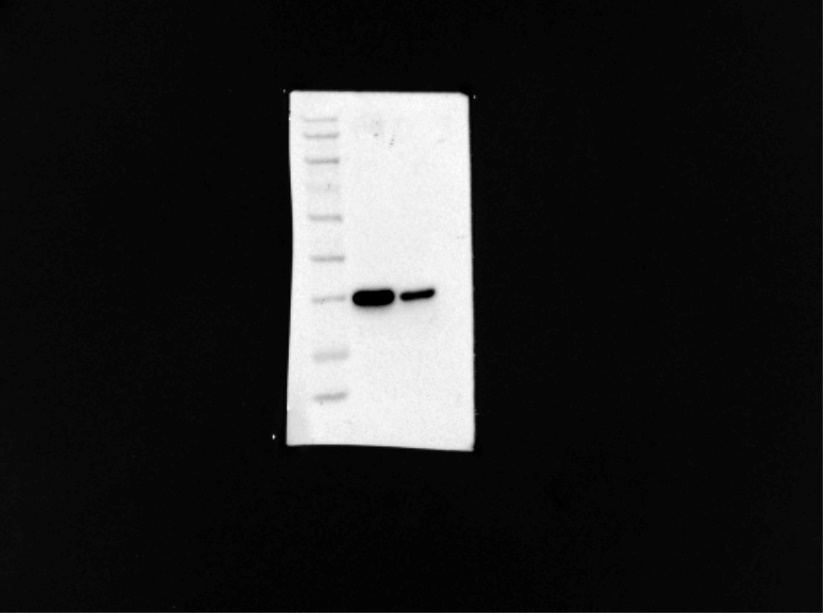


Figure 5I-5-1


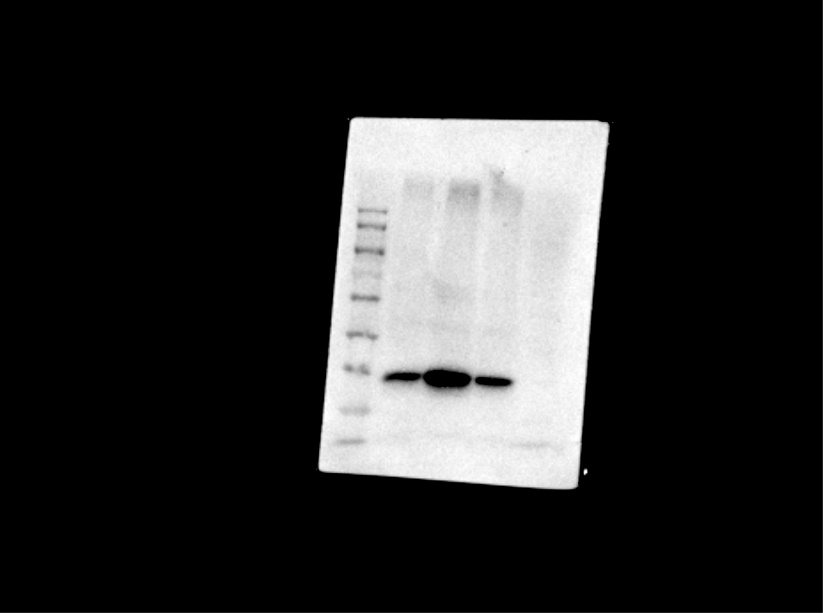


Figure 5I-5-2


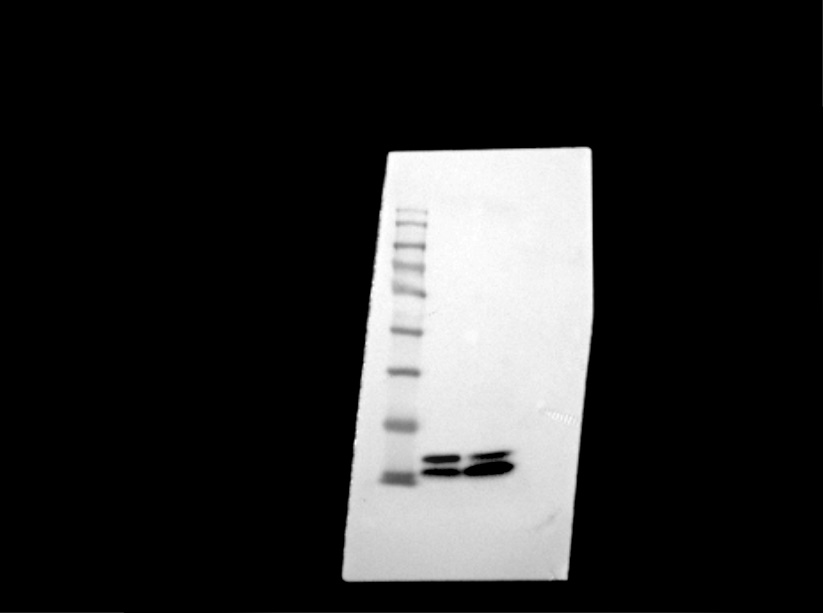


Figure 5I-6-1


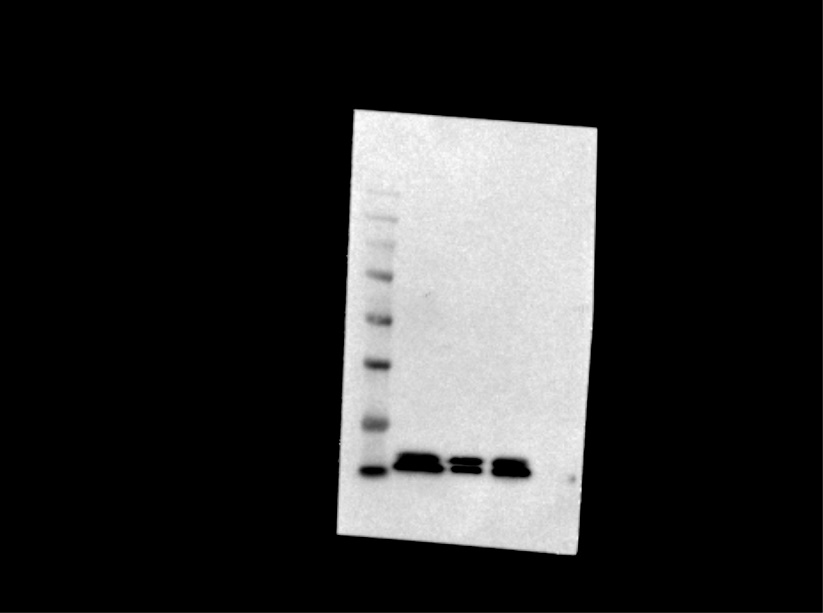


Figure 5I-6-2


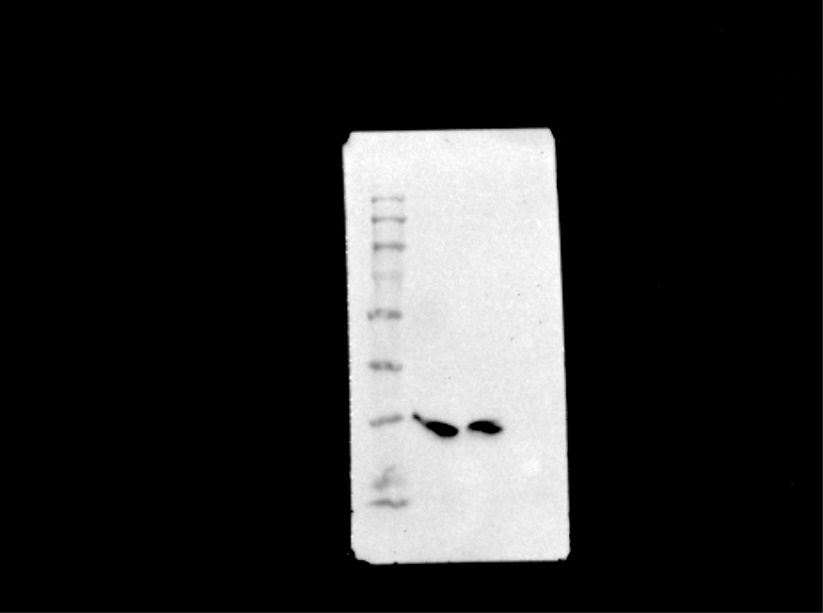


Figure 5I-7 (1)


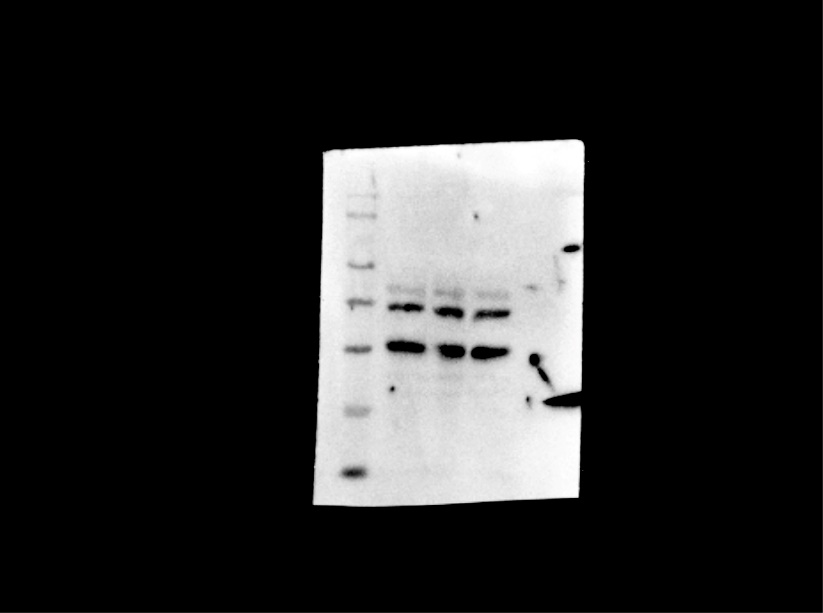


Figure 5I-7 (2)


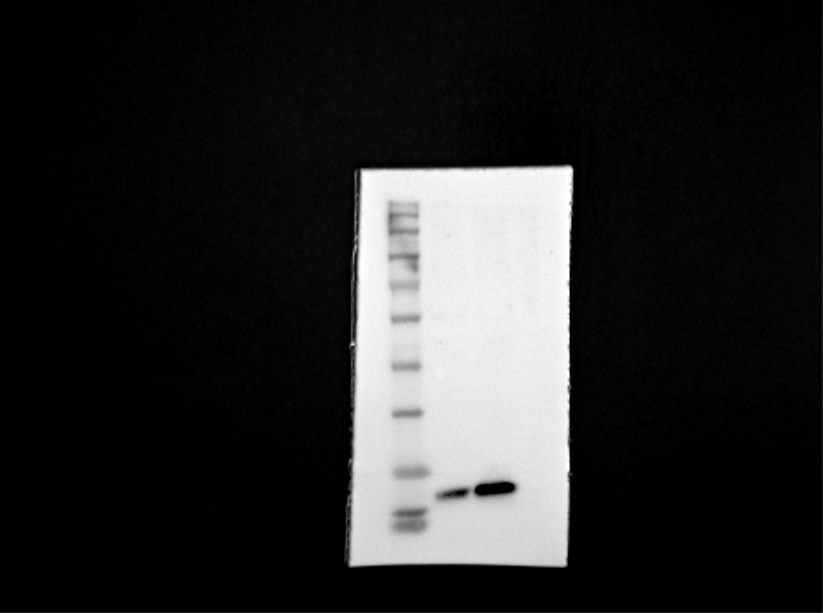


Figure 5I-8-1


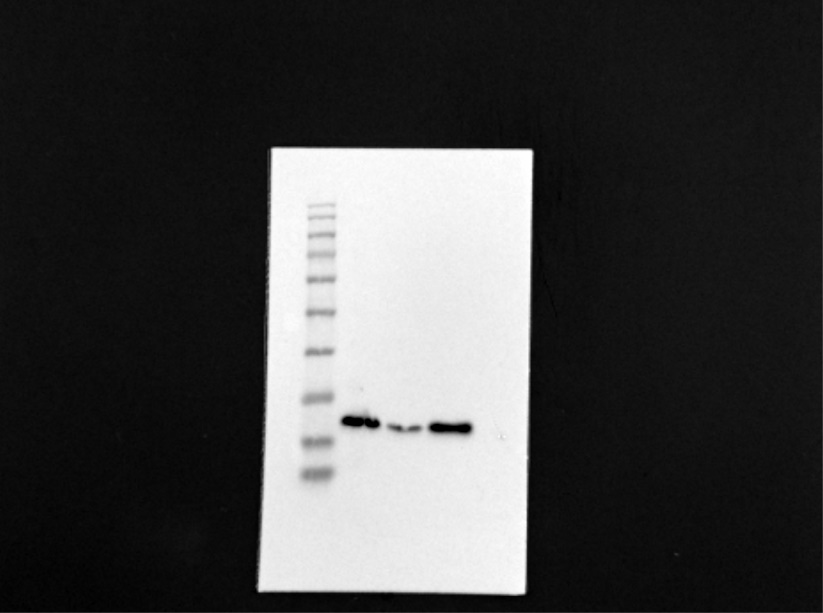


Figure 5I-8-2


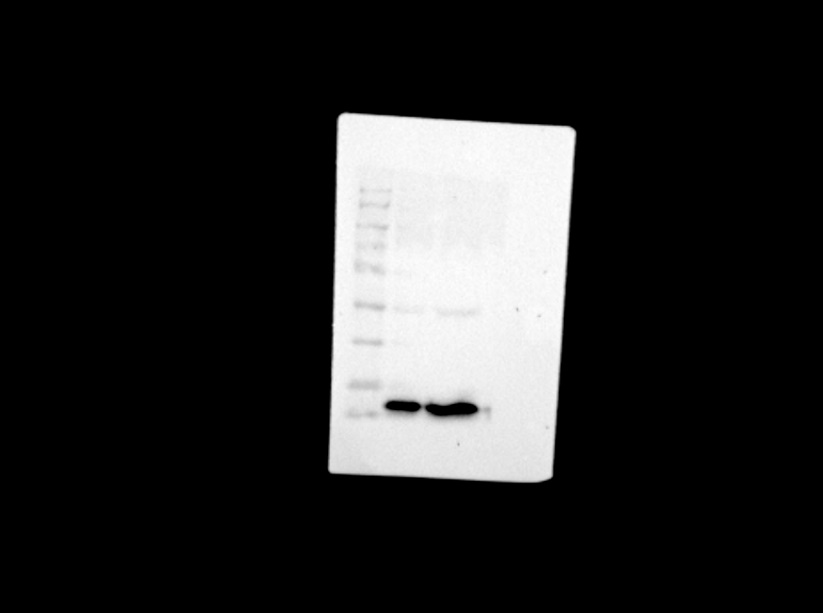


Figure 5I-9-1


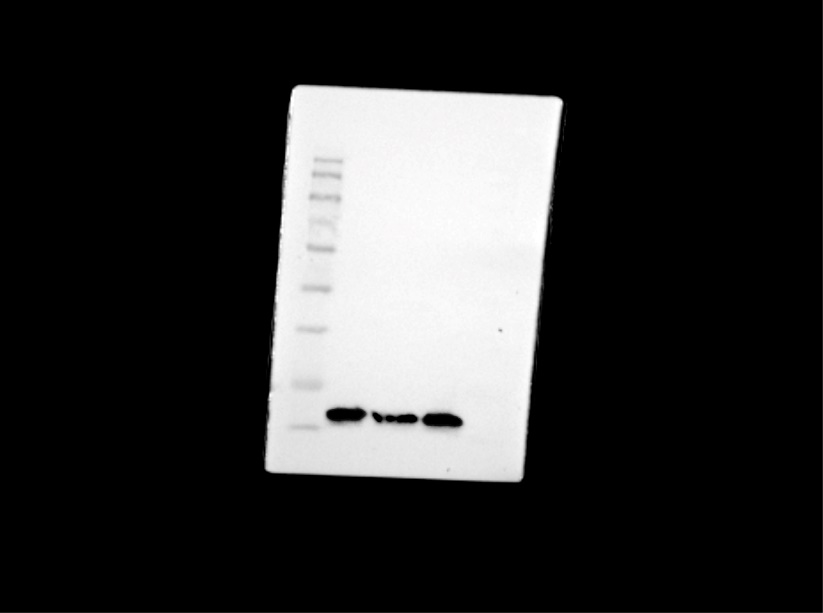


Figure 5I-9-2


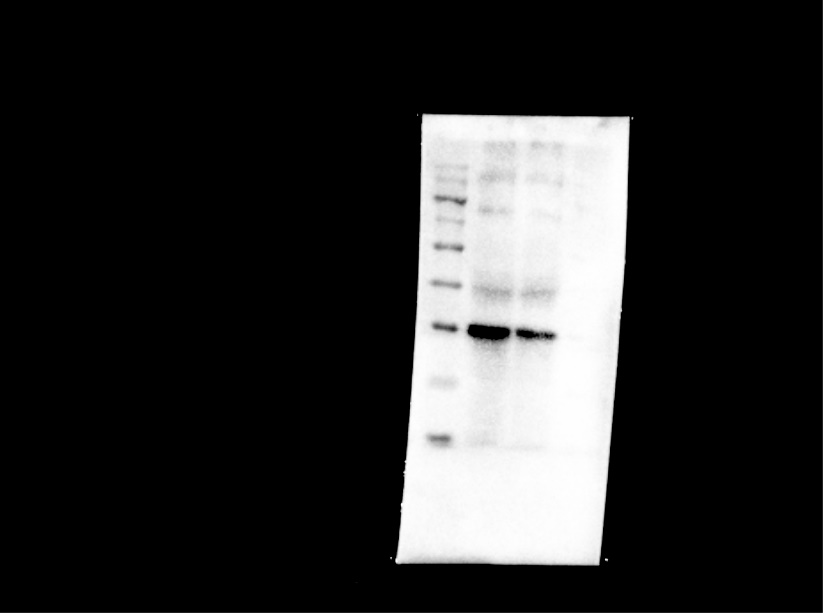


Figure 5I-10 (1)


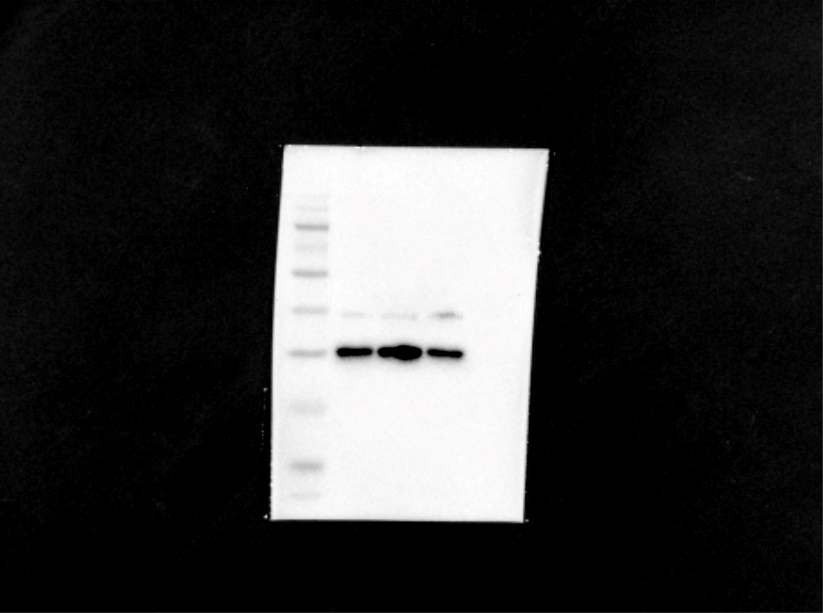


Figure 5I-10 (2)


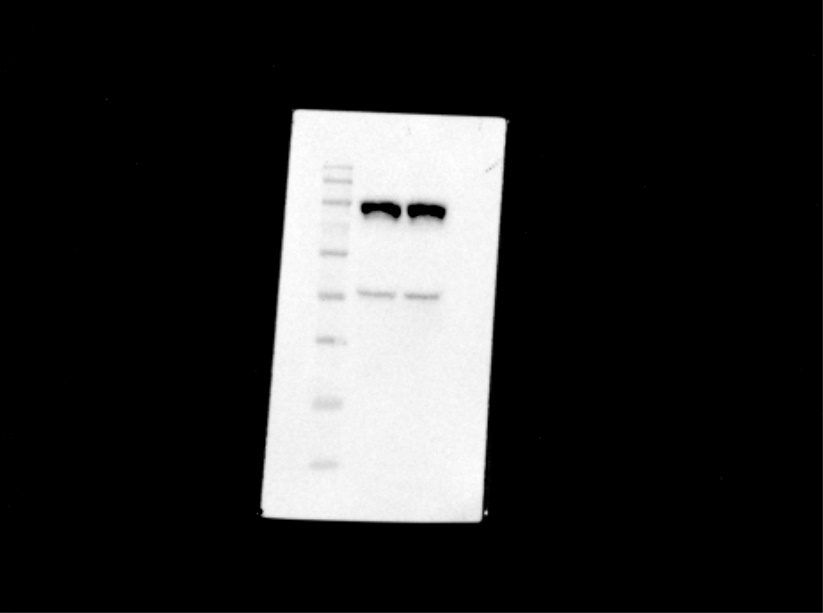


Figure 5I-11-1


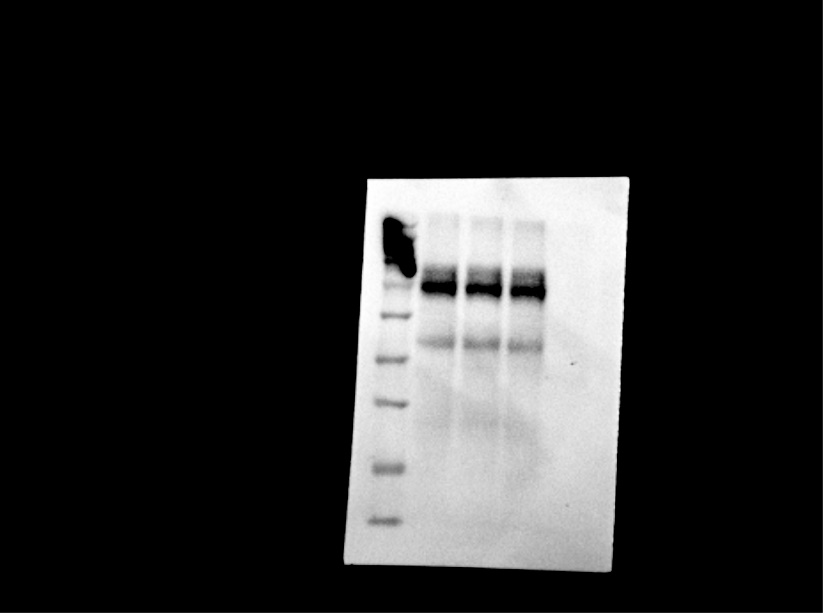


Figure 5I-11-2


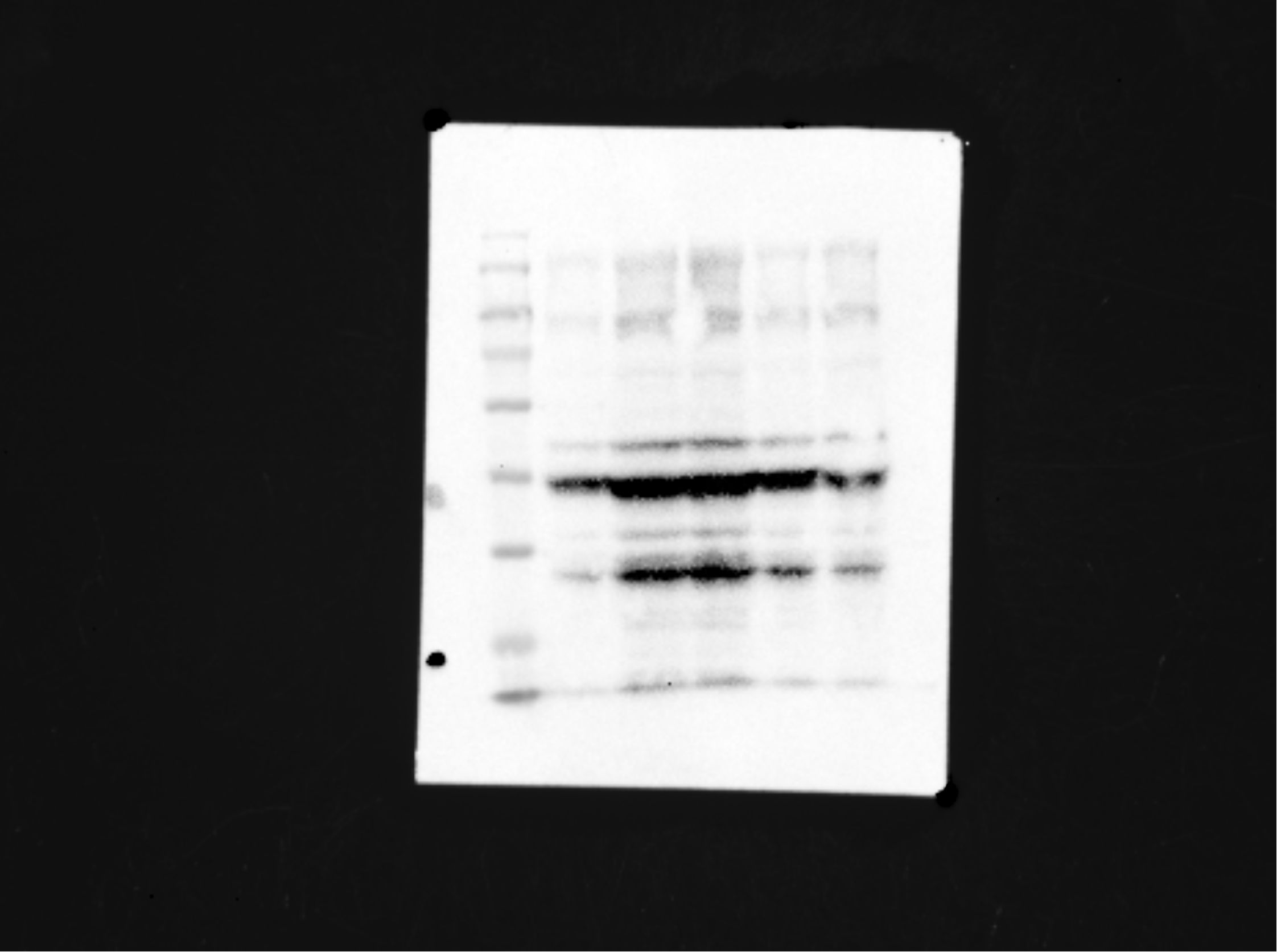


Figure 6A-1


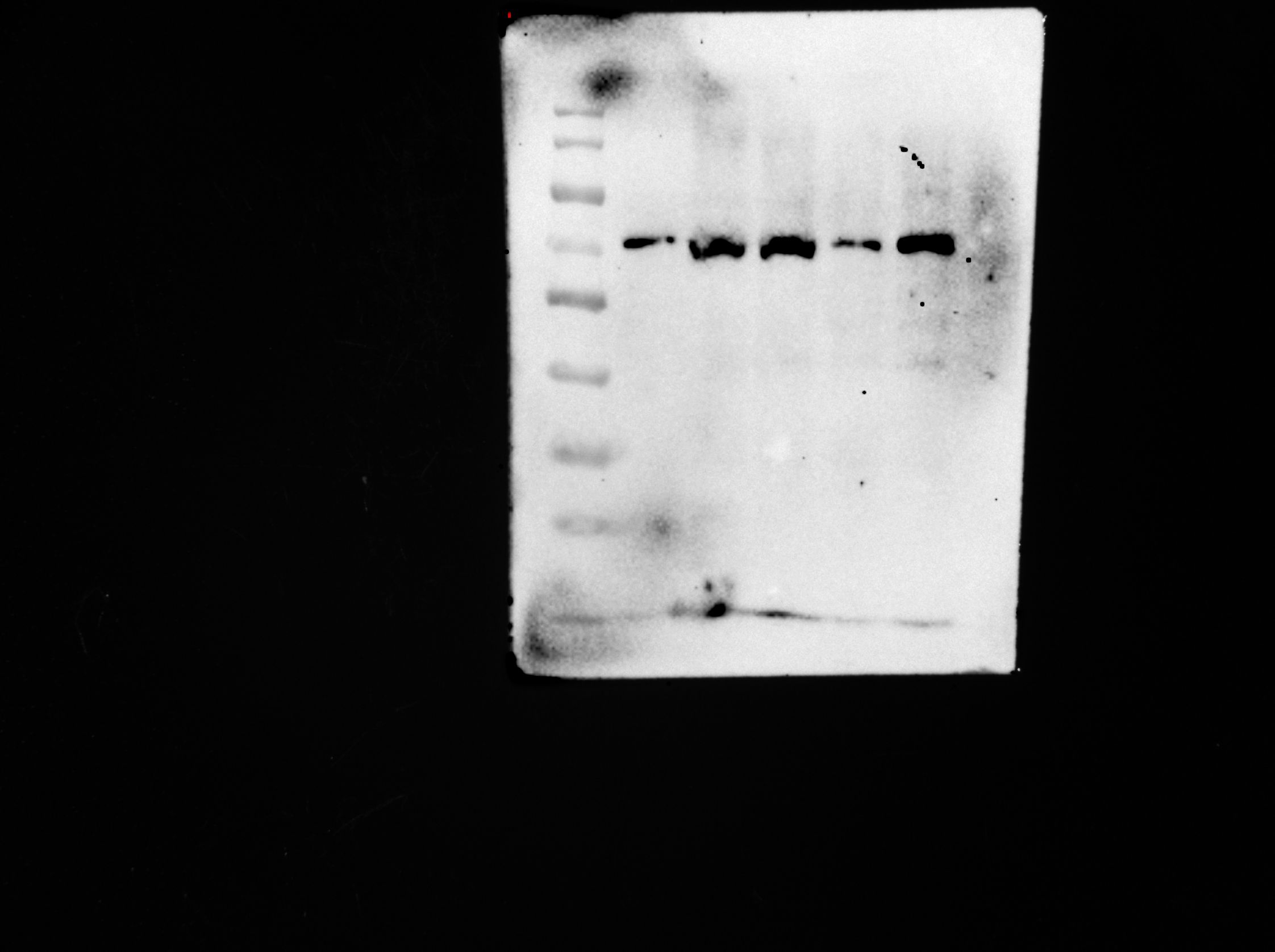


Figure 6A-2


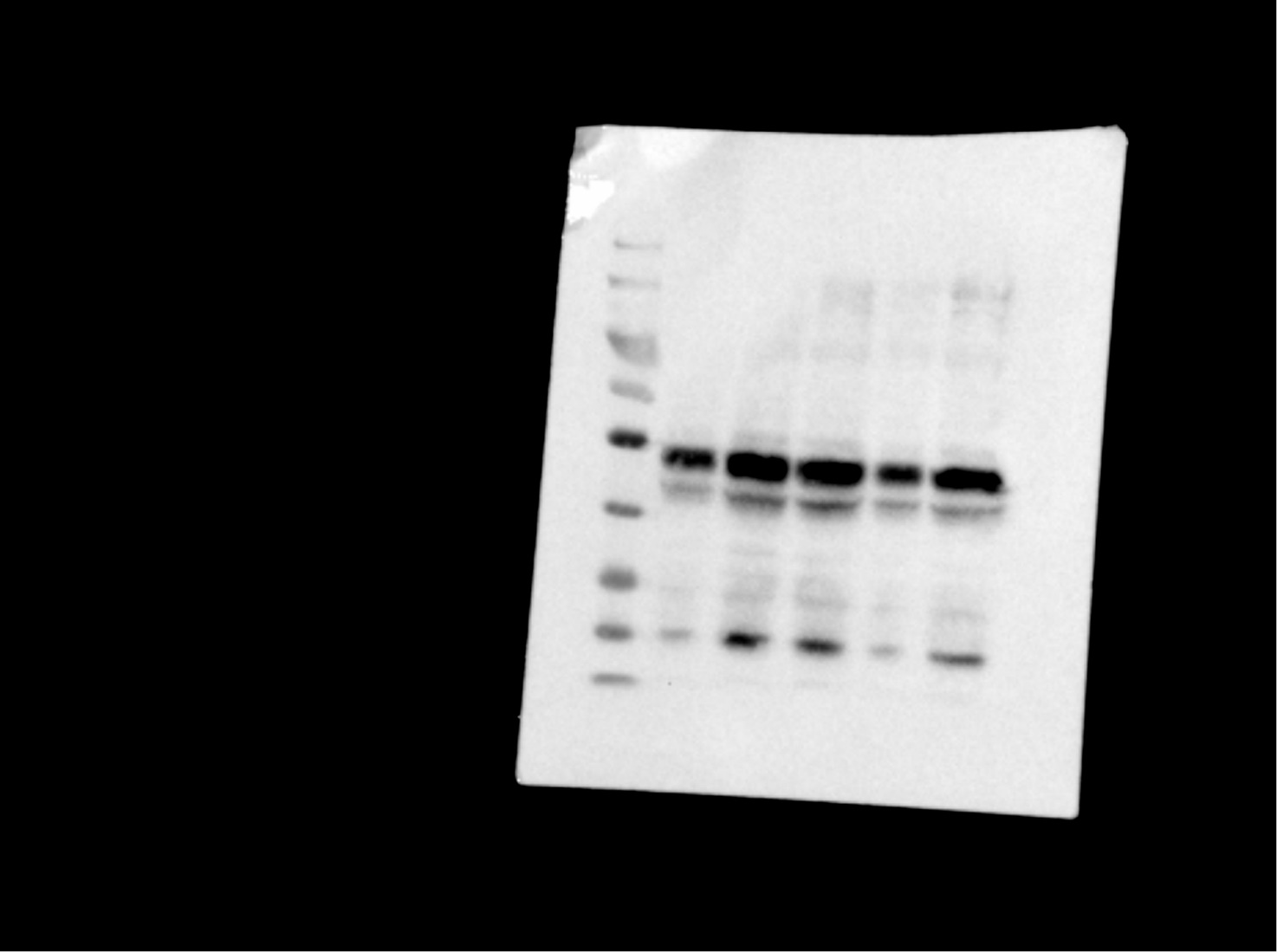


Figure 6A-3S


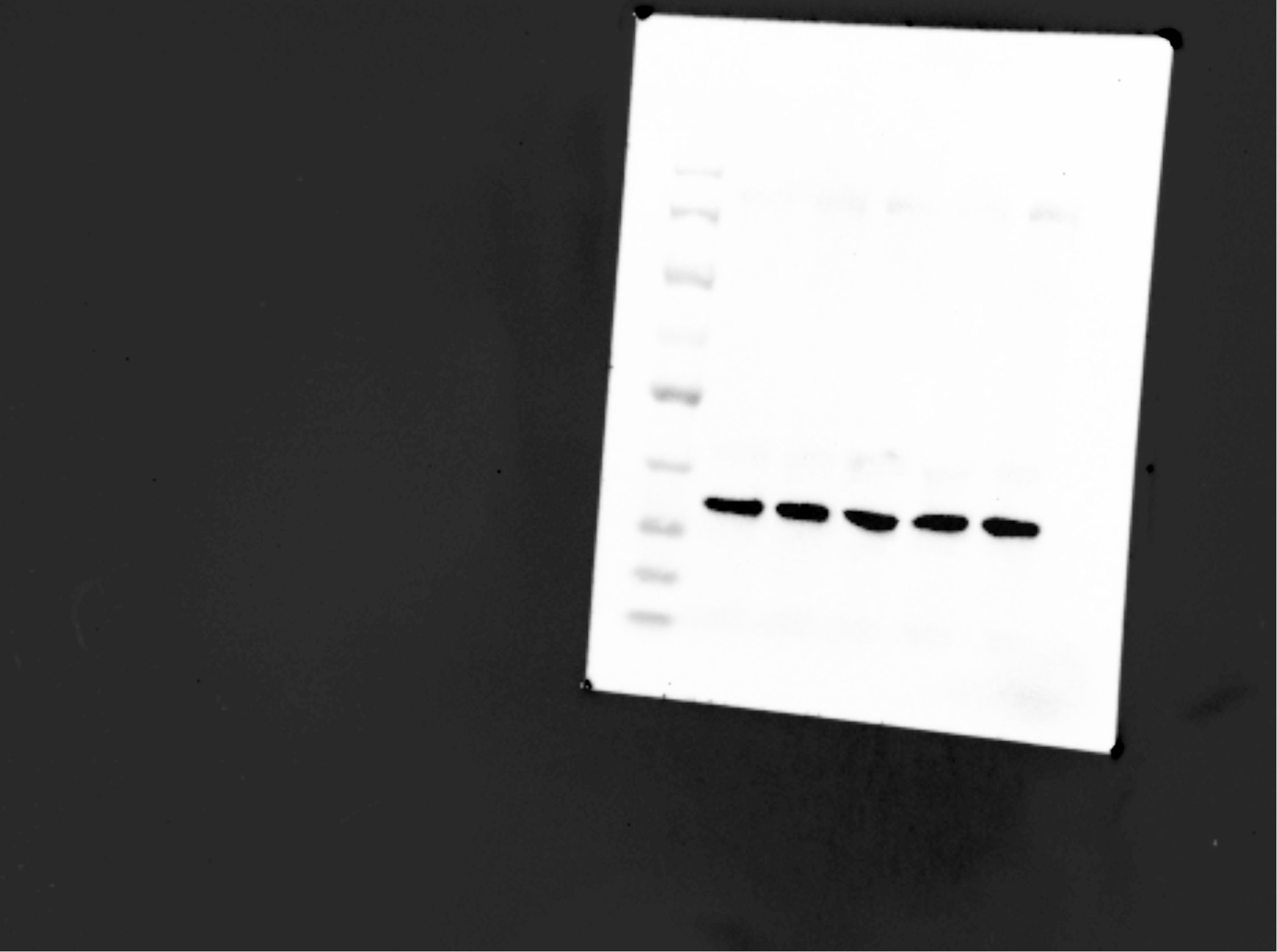


Figure 6A-4


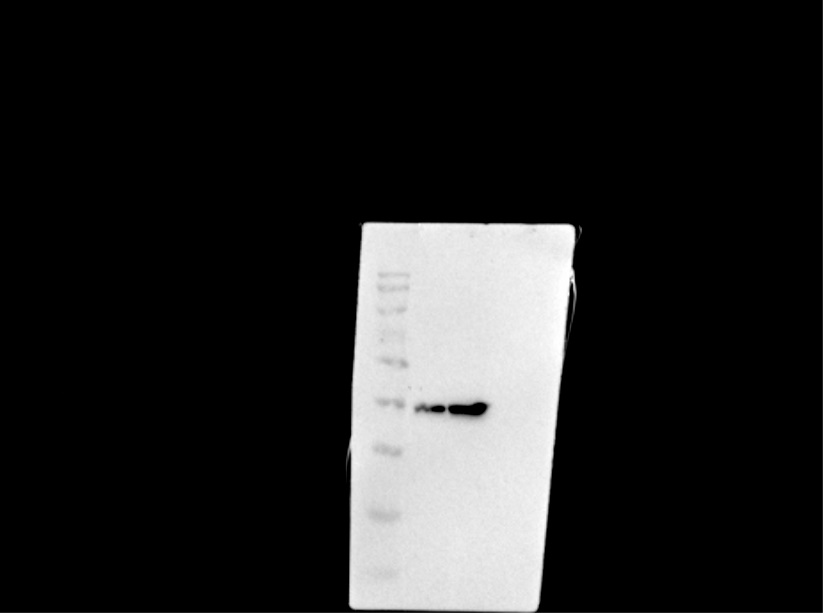


Figure 6I-1 (1)


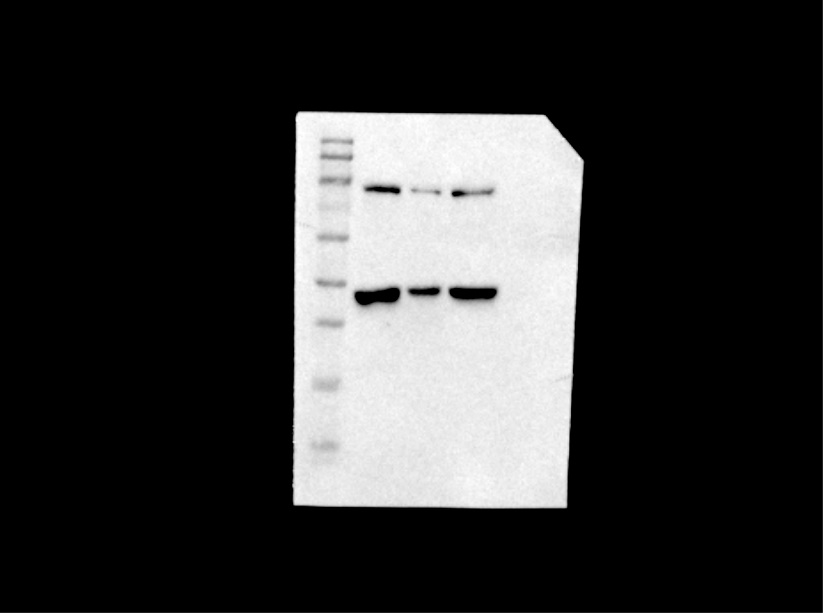


Figure 6I-1 (2)


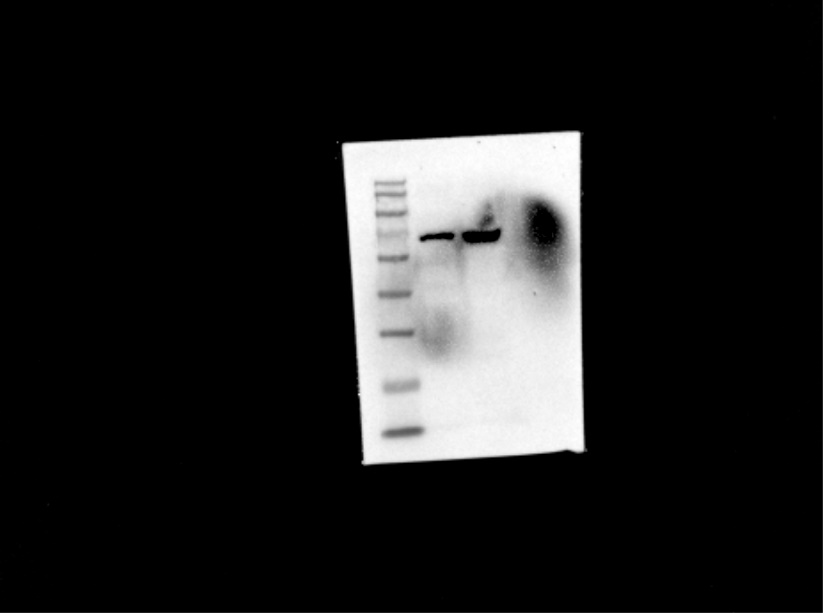


Figure 6I-2-1


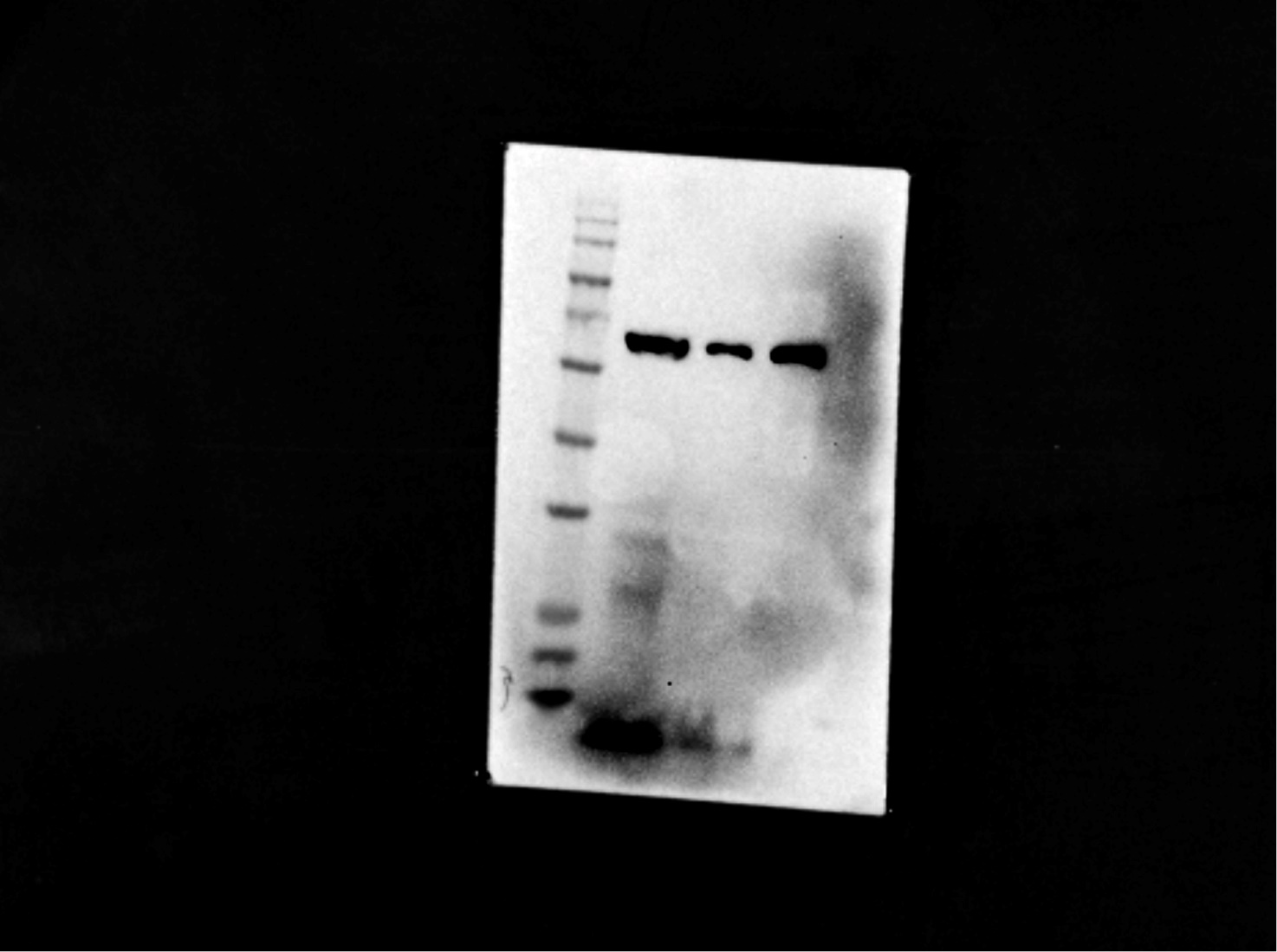


Figure 6I-2-2


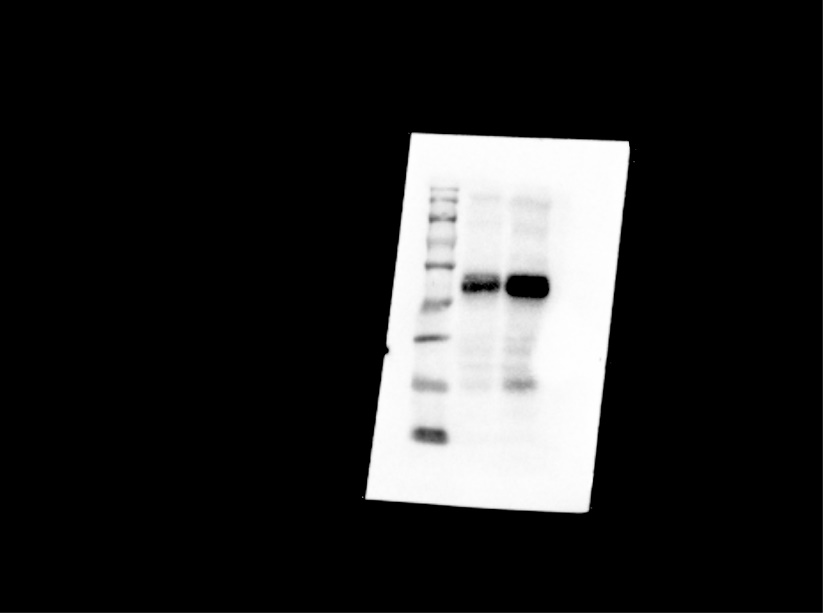


Figure 6I-3 (1)


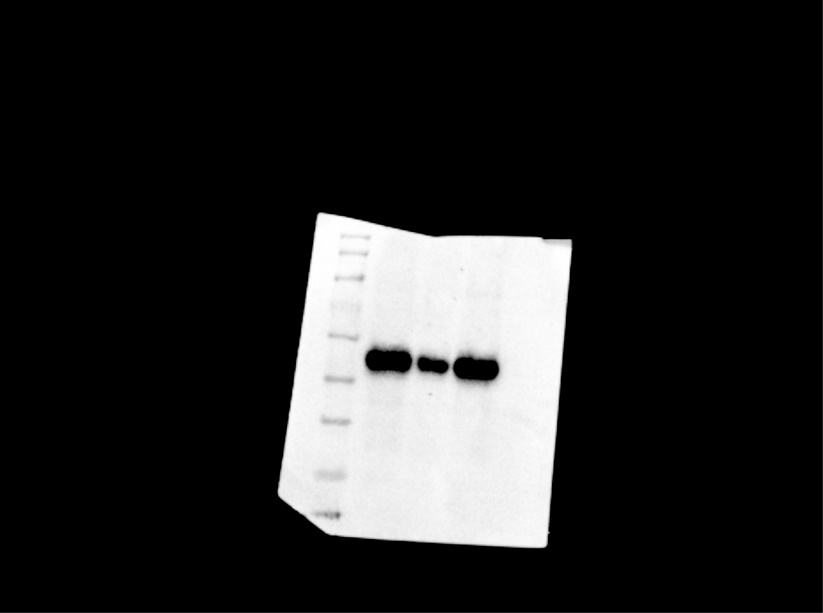


Figure 6I-3 (2)


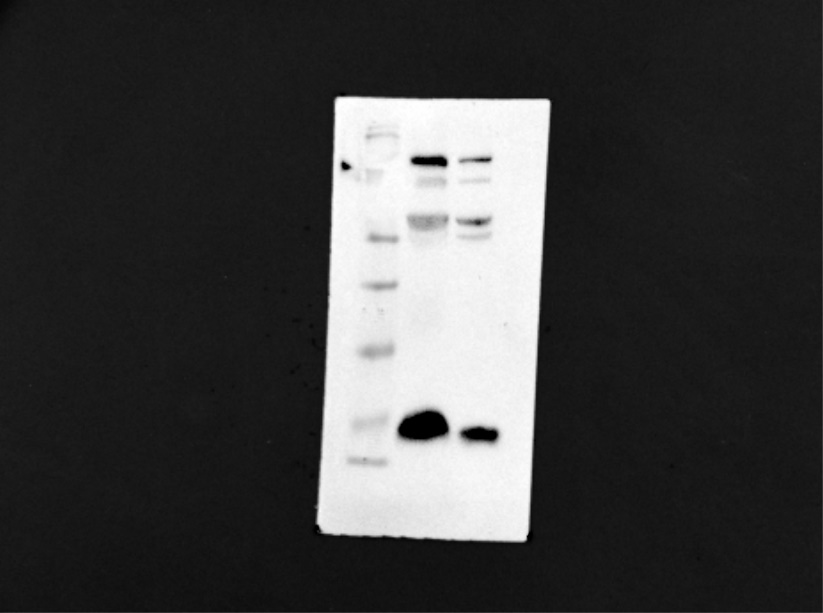


Figure 6I-4 (1)


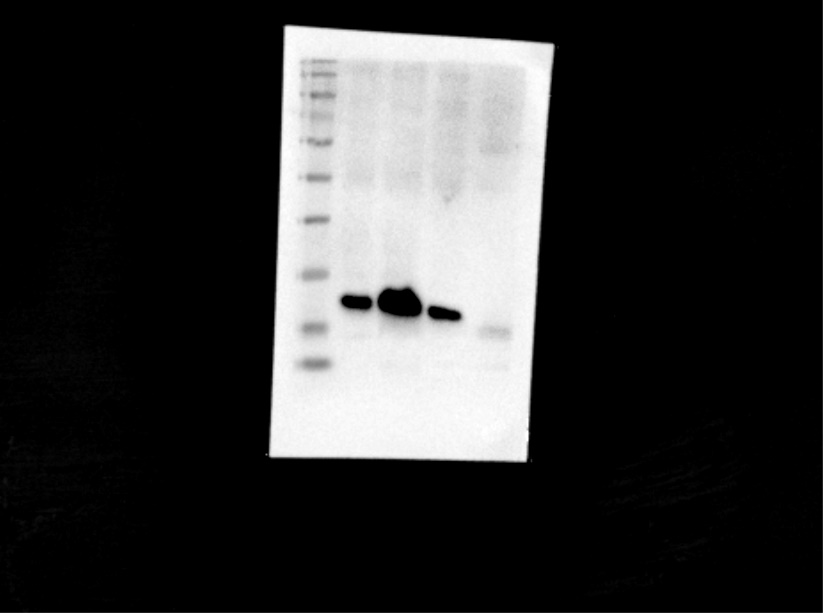


Figure 6I-4 (2)


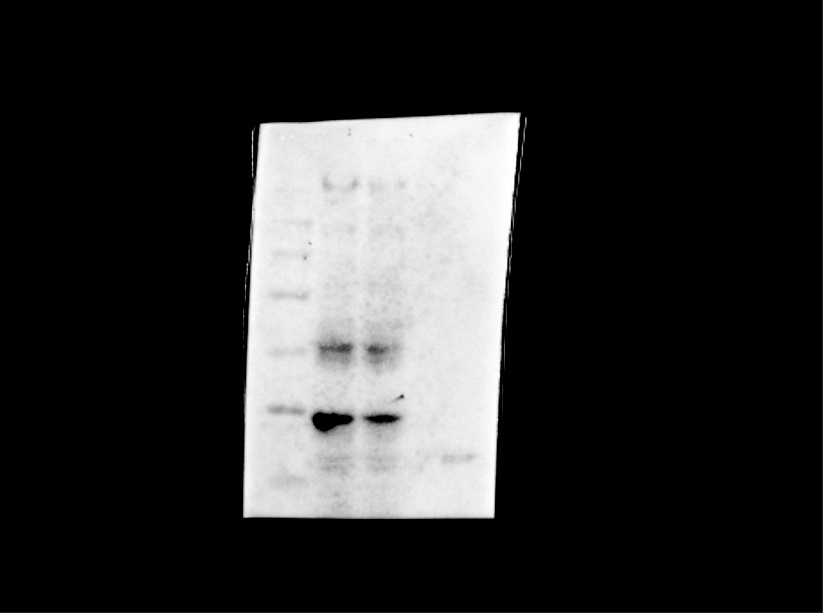


Figure 6I-5 (1)


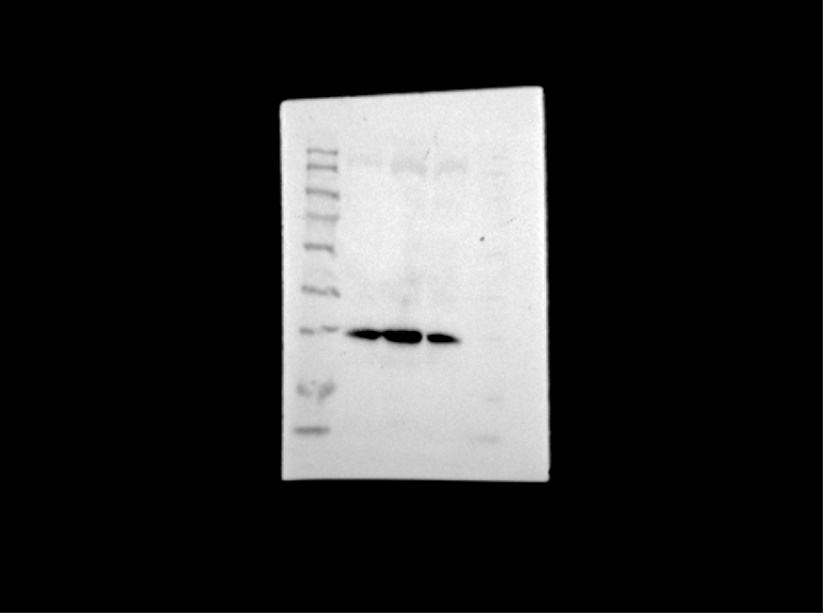


Figure 6I-5 (2)


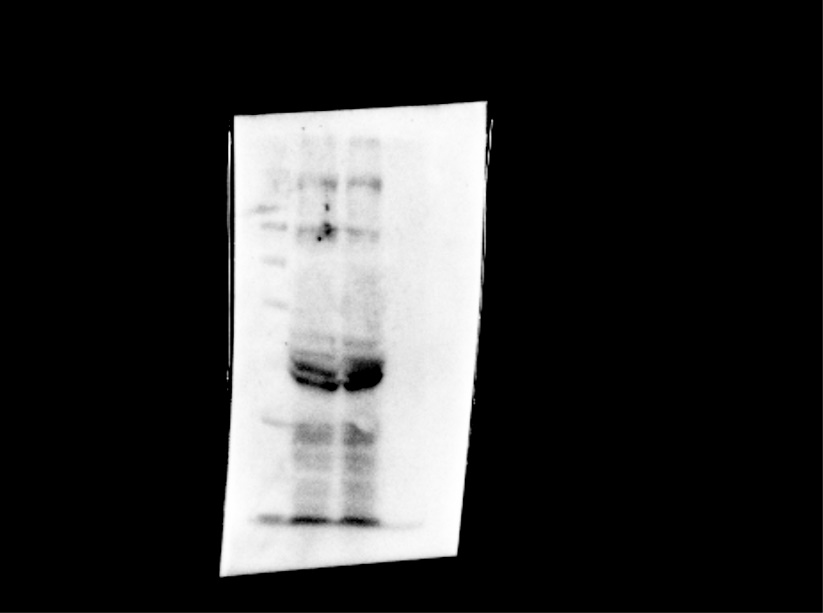


Figure 6I-6 (1)


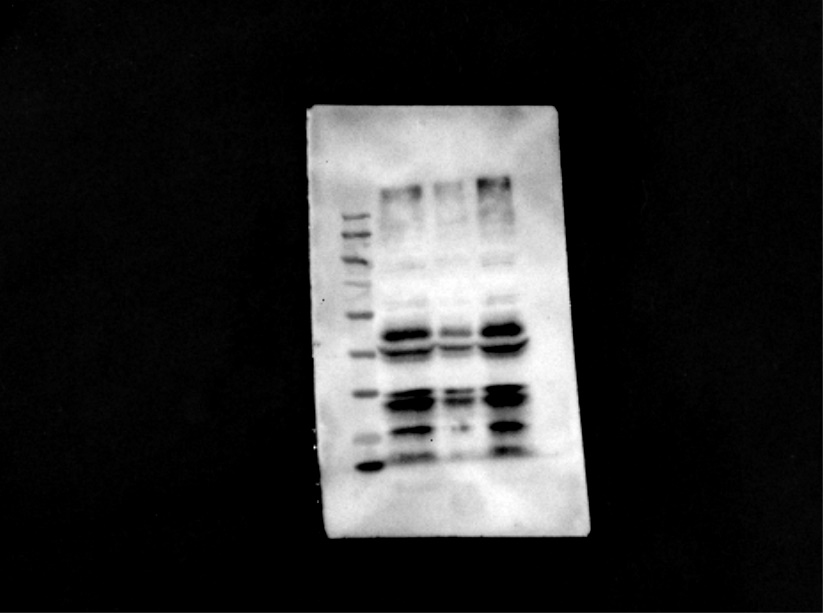


Figure 6I-6 (2)


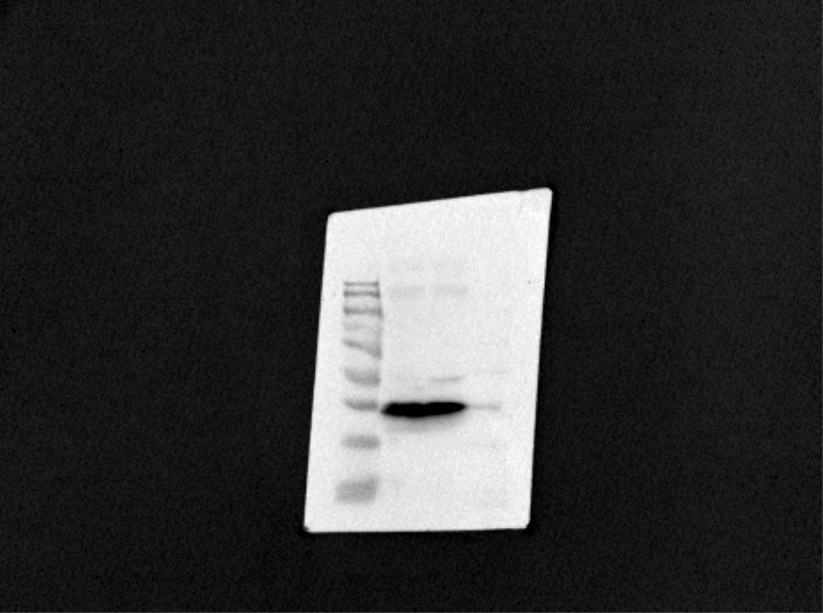


Figure 6I-7 (1)


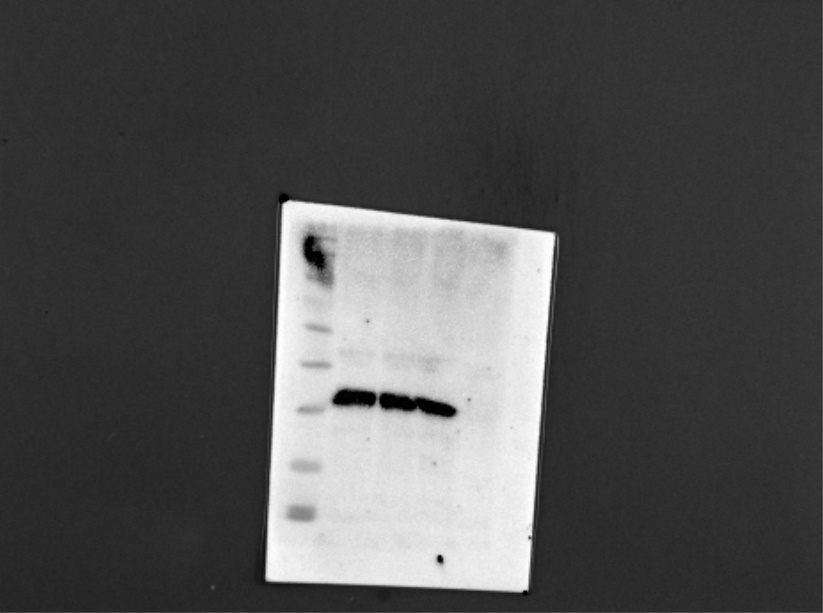


Figure 6I-7 (2)


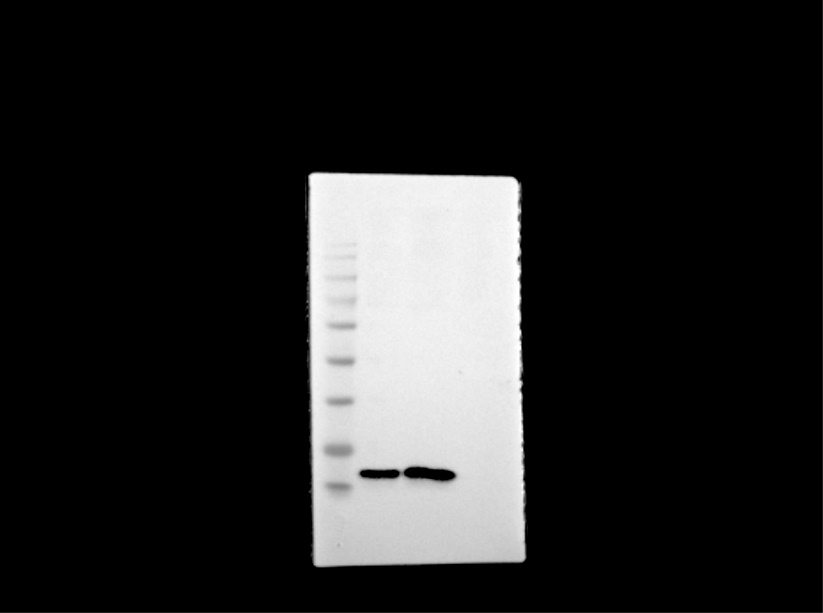


Figure 6I-8-1


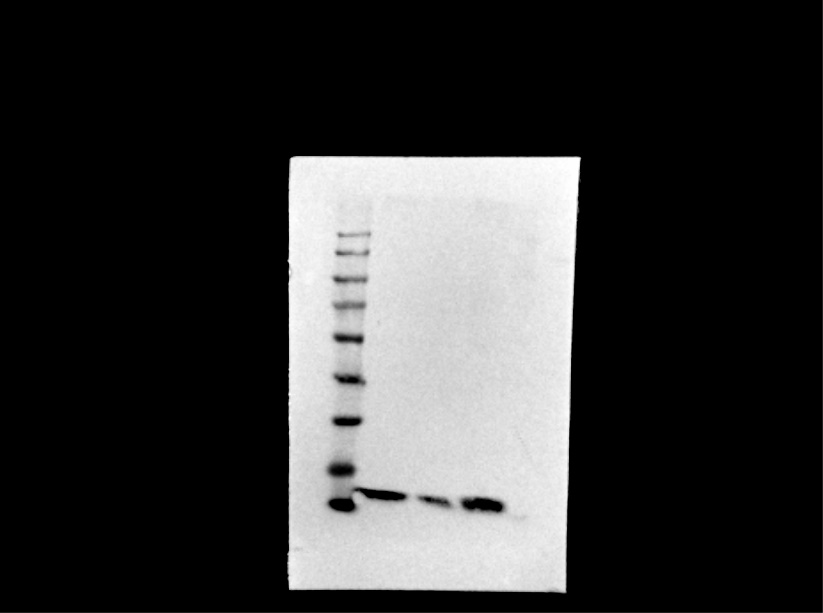


Figure 6I-8-2


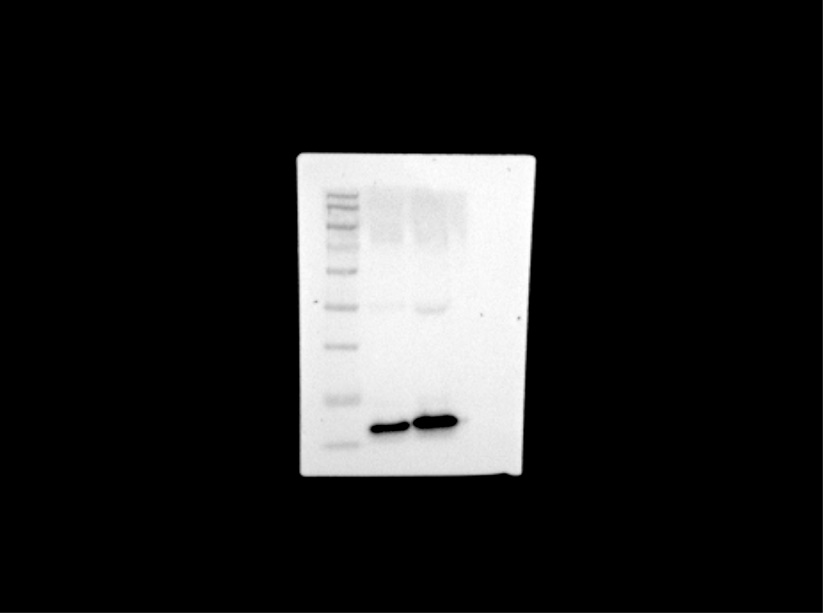


Figure 6I-9-1


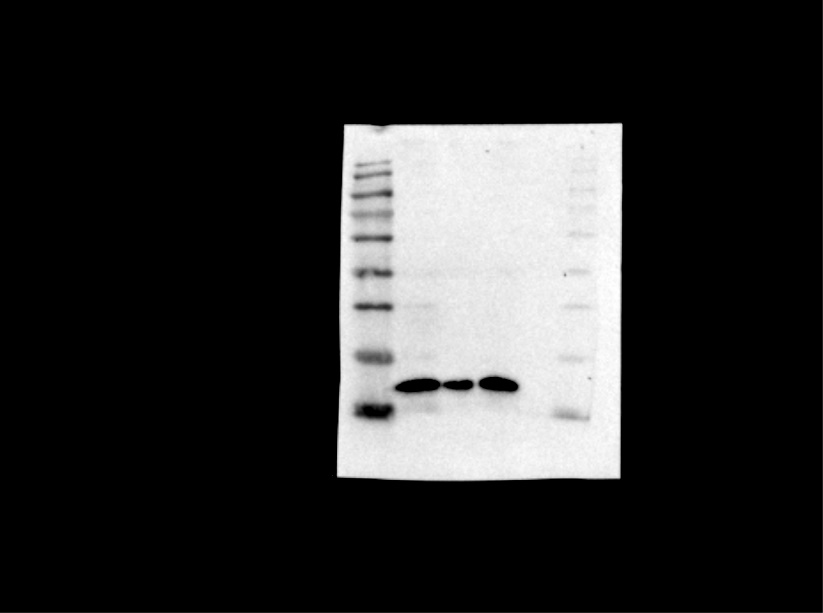


Figure 6I-9-2


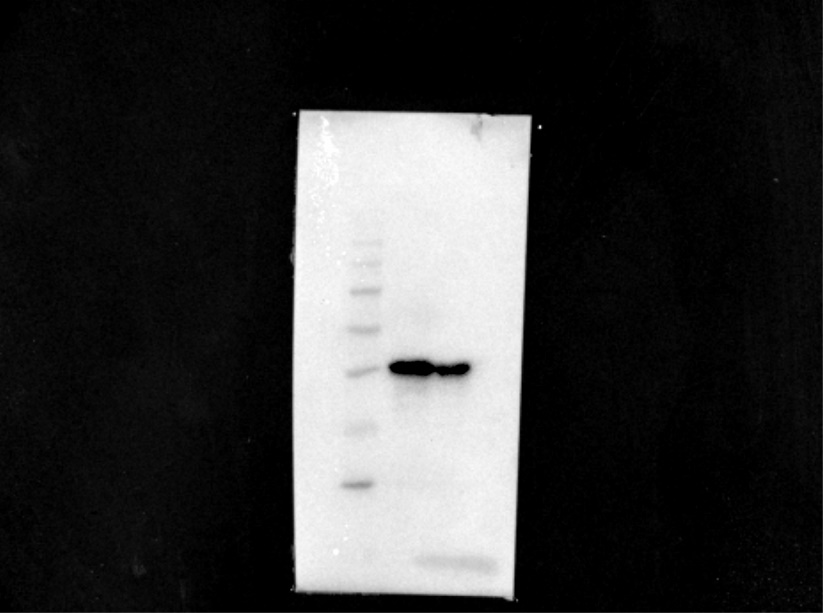


Figure 6I-10 (1)


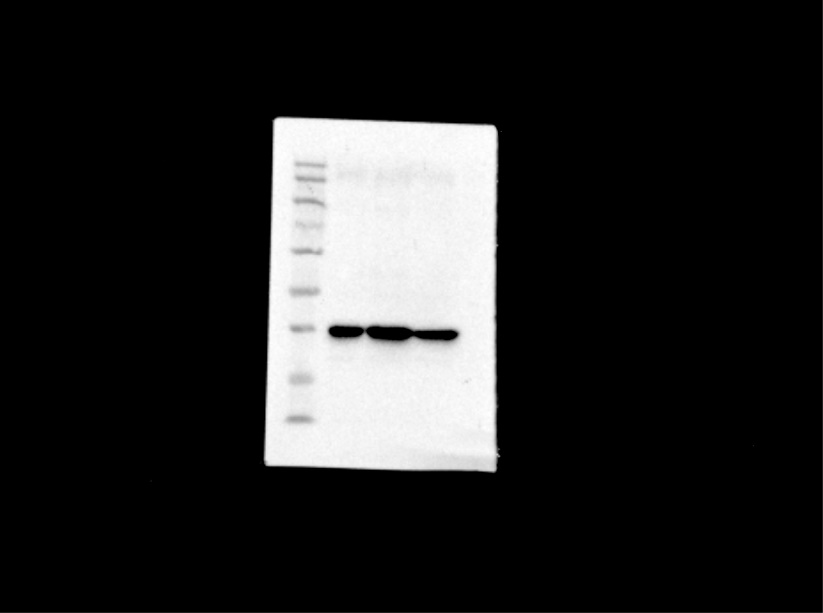


Figure 6I-10 (2)


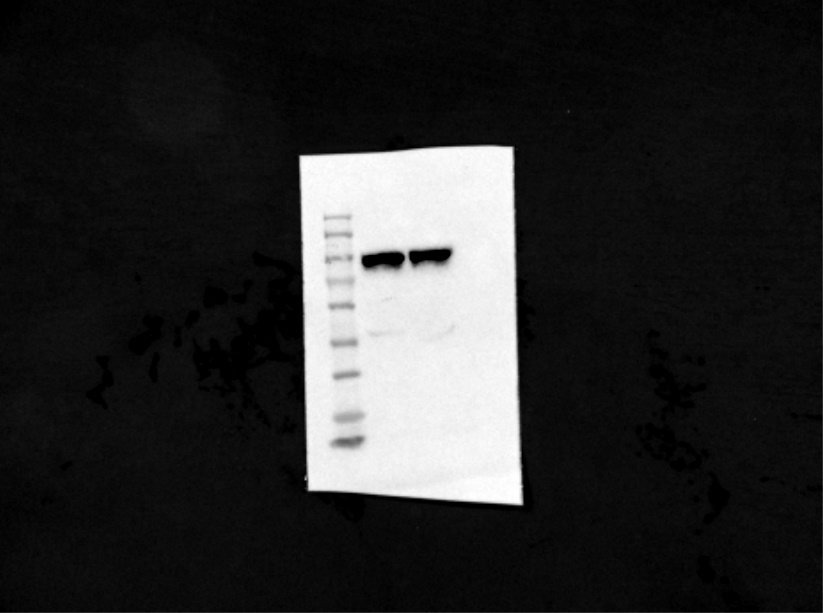


Figure 6I-11 (1)


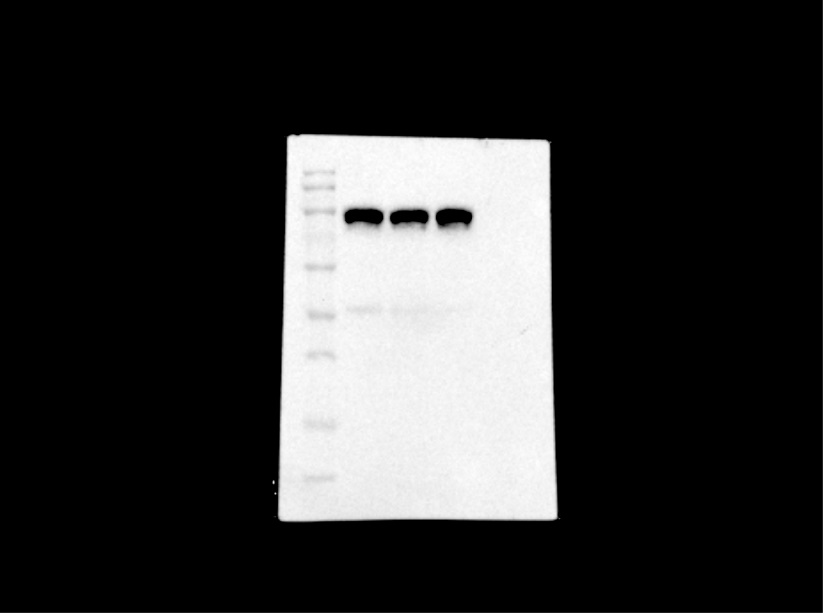


Figure 6I-11 (2)
